# The role amenities play in spatial sorting of migrants and their impact on welfare: Evidence from China

**DOI:** 10.1371/journal.pone.0281669

**Published:** 2023-02-16

**Authors:** Yunda Zhang

**Affiliations:** School of Economics, Zhejiang University, Hangzhou, Zhejiang, China; Tohoku University, JAPAN

## Abstract

From 2005 to 2015, China’s high-skilled labor was increasingly concentrated in cities with high wages and high rents, while a narrowing of the wage gap between high- and low-skilled labor showed an opposite trend to an increase in geographic sorting. In this research, I estimated a spatial equilibrium structural model to identify the causes of this phenomenon and its impact on welfare. Changes in local labor demand essentially led to an increase in skill sorting, and changes in urban amenities further contributed to this trend. An agglomeration of high-skilled labor raised local productivity, increased wages for all workers, reduced the real wage gap, and widened the welfare gap between workers with different skills. In contrast to the welfare effects of changes in the wage gap driven by exogenous productivity changes, changes in urban wages, rents, and amenities increased welfare inequality between high- and low-skilled workers, but this is mainly because the utility of low-skilled workers from urban amenities is constrained by migration costs; if migration costs caused by China’s household registration policy were eliminated, changes in urban wages, rents, and amenities would reduce welfare inequality between high- and low-skilled workers to a greater extent than a reduction in the real wage gap between these two groups.

## 1. Introduction

With the wave of college enrollment expansion in China since 1999, the education level of domestic workers has increased. At the same time, a large number of workers have left their household registration areas to work and live elsewhere. From 2005 to 2015, the proportion of migrants nearly tripled, and the proportion of college-educated workers among migrants has grown even faster than the proportion of college-educated workers nationwide, with increasingly more migrants (especially those with a higher education) choosing to live in large cities.

Wages and rents have risen in larger cities relative to smaller ones, while the wage gap between high- and low-skilled labor has narrowed over time. These facts raise some questions: What factors contribute to the growing trend of spatial sorting of the workforce? What are the agglomeration and dispersion forces, respectively? Does a narrowing of the wage gap between high- and low-skilled workers reflect similar changes in the welfare gap between these two groups?

Once workers choose to live in a city with high housing costs, the local price level may offset some of the consumption utility derived from high wages, resulting in reduced welfare for workers; alternatively, cities with high local prices may provide desirable urban amenities for workers as compensation for high rents, thereby increasing the workforce’s welfare. The impact of growing trends in spatial sorting on welfare depends on key factors that drive high- and low-skilled workers to make different choices regarding the cities in which they live.

This paper focuses on the determinants of the choice of different cities by high- and low-skilled workers under sorting trend and the welfare impacts of these choices. By estimating a spatial equilibrium structural model of local labor demand, housing supply, labor supply, and amenities supply, this paper illustrates that changes in the relative demand for high- and low-skilled labor caused by changes in local productivity are the drivers that underpin the differences in high- and low-skilled labor’s migration patterns.

While local wage changes can be (and often are) the initial cause of migration, I discovered that cities that attract a disproportionate amount of high-skilled labor will endogenously become more desirable places to live and more productive for all workers living in them. A combination of desirable wages and amenities makes high-skilled workers willing to pay high housing costs to live in these cities. While low-skilled workers also find good wages and amenities desirable, they are unwilling to pay such high living costs, and they have more difficulty accessing adequate urban amenities. Consequently, after weighing the pros and cons, they may choose a more desirable city.

Overall, this paper finds that, as migration costs limit migrants’ (especially low-skilled workers’) access to local amenities, the welfare effects of changes in local wages, rents, and endogenous amenities lead to increased welfare inequality between high- and low-skilled workers. When migration costs are eliminated and workers get full access to urban resources, the welfare effects of changes in local wages, rents, and endogenous amenities reduce welfare inequality, and the reduction in welfare inequality is greater than the reduction in the real wage gap between high- and low-skilled workers.

This paper builds on Diamond’s (2016) [1] urban spatial equilibrium structure model by adding settings related to migration costs across cities and characterizing utility losses derived from migrants’ limited use of urban amenities due to a lack of local household registration as well as competition with other residents for urban resources. The model adds heterogeneous labor preferences to cities based on the frameworks of Rosen (1979) [2] and Roback (1982) [3]. I used a static discrete choice setup to simulate the labor force’s city choices. This model allows workers with different demographics to weigh the relative value of urban features in different ways, which leads them to make different siting decisions.

In this paper, workers with a college education were defined as high-skilled labor, and workers with a high school or less education were defined as low-skilled labor. There are differences in the local productivity levels of high- and low-skilled labor, and the productivity levels of high- and low-skilled labor are influenced by the skill-mix of the city. Thus, changes in the urban skill-mix affect local wages by changing firms’ labor supply and demand, and by directly affecting labor productivity. Firms in each city use capital and workers as inputs for production. Housing markets differ across cities due to the heterogeneity of housing supply elasticities.

In addition to treating wages and housing costs as endogenous factors, I allowed amenity supply to respond to skill-mix of the city. To measure urban amenities levels as comprehensively as possible, I collected data on seventeen different amenities in seven categories. I used an autoencoder (AE) to combine these seventeen data sources into a single amenity index.

A two-step estimation method was used to estimate workers’ preferences for cities, similar to the setup proposed by McFadden (1973) [4] and the method used by Berry et al. (2004) [5]. First, a conditional logit method was used to determine the average desirability of each city for each type of worker every five years. Then a nonlinear generalized method of moments (GMM) was used to estimate the model, and in this step the estimated utility levels of workers living in each city were used as a dependent variable to estimate how workers trade off wages, rents, and amenities when choosing where to live.

Endogeneity was addressed using local labor demand shocks driven by industrial structure of each city and its interaction term with local housing supply elasticities as instrumental variables. According to the industrial composition of urban employment, differences in productivity changes across industries will have different effects on the demand for high- and low-skilled workers in cities (Bartik, 1991) [6]. Exogenous local productivity changes were measured by interacting differences in composition of employment across industries with changes in industry average wages for high- and low-skilled labor, respectively. Following the literature (Saiz, 2010; Gyourko et al., 2008) [7, 8], I set the elasticity of the urban housing supply to vary according to the geographic constraints of developable land around urban centers and land use regulations. The elasticity of a city’s housing supply impacts equilibrium wages, rents, and population.

This paper is related to the literature in several directions. Most closely related is the literature that studies how local wages, rents and employment respond to local labor demand shocks (Saks, 2008; Moretti, 2011; Albouy and Stuart, 2020; Notowidigdo, 2020; Monras, 2020; Piyapromdee, 2021) [9–14]. The literature on traditional topics generally only allows for local labor demand shocks to impact labor migration via changes in wages and rents (Saks, 2008; Moretti,2011; Monras, 2020) [9, 10, 13]. Recent literature has begun to focus on the role of changes in urban amenities, apart from wages and rents, in the impact of local labor demand shocks on labor migration(Albouy and Stuart, 2020; Notowidigdo, 2020; Piyapromdee, 2021) [11, 12, 14].

Taken together, the literature focusing on the role of urban amenities in the process of labor demand shocks affecting labor migration generally concludes that the level of urban amenities is a positive driving force for labor agglomeration. On this basis, the findings of this paper further suggest that endogenous local amenity changes are an important mechanism that drives labor migration in response to local labor demand shocks.

A growing body of literature has studied how amenities vary with the composition of an area’s residents. (Bayer et al., 2007; Brueckner and Rosenthal, 2009; McKinnish et al., 2010; Guerrieri et al., 2013; Handbury, 2021) [15–19]. Handbury (2021) [19] provided direct evidence that the products and prices offered in the local market are related to the tastes of different income groups. A large amount of urban economics literature argues that these tastes help explain observed spatial disparities in income and skills across cities (Glaeser et al., 2001; Couture and Handbury, 2020) [20, 21]: High-skilled and high-income workers tend to make similar decisions about location because they enjoy more utility from locally endogenous amenities than do low-skilled, low-income workers. In this paper, this premise is one of the main forces driving the spatial sorting trend of high- and low-skilled labor. This paper provides empirical support for the theory with the help of a spatial equilibrium model, that changes in skills-biased amenities are the result of reconciling changes in rents and wages with observed changes in the skills composition of a city. A similar procedure is found in Black et al.(2009) [22].

The findings of this paper are also relevant to the literature that studies changes in wage structures and inequality within and between local labor markets (Moretti, 2013; Autor and Dorn, 2013; Autor et al., 2013; Baum-Snow and Pavan, 2012; Piyapromdee, 2021; Baum-Snow et al., 2018) [14, 23–27]. Of the above literature, the most relevant to this paper is Moretti (2013) [23], who is the first to illustrate the importance of considering the different location choices of high- and low-skilled labor when measuring changes in real wage and welfare inequality.

Another thread of the literature, specifically related to the labor demand estimates in this paper, studies the impact of the relative supply of high- and low-skilled labor on their wages (Card, 2009; Dustmann et al., 2013; Lewis, 2011; Dustmann and Glitz, 2015; Llull, 2018; Foged and Peri, 2016) [28–33]. A strand of literature represented by Card (2009) [28] focused on wage and welfare inequality between high- and low-skilled labor. This paper follows the identification strategy proposed by Diamond (2016) [1], which differs from the traditional hedonic method of estimating labor demand at the city level and takes into account endogenous productivity changes.

The labor supply model and estimation take advantage of the discrete choice method developed in the empirical literature on industrial organizations (McFadden, 1973; Nevo, 2001; Fan, 2013; Busso et al., 2013; Berry and Haile, 2014) [4, 34–37]. This method has also been used in much of the regional economics literature (Bayer et al., 2007; Bayer et al., 2009; Kennan and Walker, 2011) [15, 38, 39]. However, the models of Bayer et al. (2009) [38] and Kennan and Walker (2011) [39] do not allow local wages and rents to be related to local amenities. In this paper, I used this method to estimate the determinants of urban labor supply.

This paper is organized as follows. Section 2 discusses the data and variables, Section 3 presents the stylized facts, Section 4 builds the model, Section 5 discusses the model’s estimation techniques, Section 6 presents the parameter estimation, Section 7 discusses the estimation of urban amenities and productivity, Section 8 analyzes the impact of registered population on location choice. Section 9 analyzes the determinants of urban high-skilled labor employment ratio changes, Section 10 presents potential implications for welfare, and Section 11 concludes.

## 2. Data and variables

### 2.1. Data sources

This research used 2010 census data as well as 2005 and 2015 mini-census data to calculate the migration flow of labor that has different skill levels, housing rents, and other related items across cities. The 2000 census data were also used in the stylized facts to accurately capture the trends of some indicators. I also used the China Urban Statistical Yearbook, the China Urban Construction Statistical Yearbook, the China Regional Economic Statistical Yearbook, the China County (City) Social and Economic Statistical Yearbook, the China County Statistical Yearbook, and the statistical yearbook of each city and province to obtain comprehensive city-level data. Also, data from the China Migrants Dynamic Survey (CMDS) were used for some indicators.

The target population of this paper was migrants in China. According to the CMDS definition of migrants, migrants are those who have lived in an inflow area for more than one month, whose household registration is not registered in the local district, and are over 15 years old. In this paper, samples were selected from the dataset according to this definition.

The scope of a city in the manuscript is an entire city at the prefecture level. There are two reasons for not using municipal districts (*shixiaqu* in Chinese) as the scope criteria. On the one hand, the official census microdata provided by the National Bureau of Statistics is desensitized, and it provides 4-digit address codes that are accurate down to the prefecture level of a city. If a further subdivision is desired, the dataset provides urban and rural classification codes, which contain three categories, namely “*cheng*”, “*zhen*”, and “*xiang*,” corresponding to urban areas, towns, and villages, respectively. However, the scope of “*cheng*” or the scope of “*cheng*” and “*zhen*” is not the same as the scope of a municipal district (*shixiaqu*) defined in the statistical yearbook. Therefore, it is inappropriate to simply keep the data of “*cheng*” or to keep the data of “*cheng*” and “*zhen*” and use them as the data of municipal districts. On the other hand, if the difference in scope is ignored, directly combining “*cheng*” and “*zhen*” and treating the combined data as data of the municipal district, excluding the data of “*xiang*”, a large number of samples would be discarded (53% in 2005, 51% in 2010 and 42% in 2015), which would lead to a shortage of the number of high-skilled workers in many cities, reducing the number of cities analyzed by nearly half and significantly affecting the accuracy of the empirical results.

When calculating the local good expenditure share, there are different choices regarding the scope of local goods, with some choosing to consider housing as a local good (Davis and Ortalo-Magné, 2011; Wang and Li, 2015) [40, 41]. Others choose to consider both housing and nonhousing commodities as local goods (Albouy, 2008; Lewbel and Pendakur, 2009; Moretti, 2013) [23, 42, 43]. These choices were discussed in the Parameter Estimation section. The required data came from the census and the CMDS. I noted that there were only 106 cities in the 2010 CMDS survey. Therefore, for some parameters that need to be calculated using the 2010 CMDS data, I chose to calculate them by deflating the 2011 CMDS data with an index, such as the wage index.

### 2.2. Imputed city-skill level wages by the weighted method

For the empirical part of this study, I needed the average wages of the labor force with different skills in different cities in 2005, 2010, and 2015. However, wages are not counted in any census other than the 2005 census. Therefore, I could not directly calculate the average wage of the labor force with different skills in each city based on census data. To solve this problem, I used the weighted method of Fang and Huang (2022) [44] to calculate the average wages of different skills across cities by weighting the wages of different industries provided by the statistical yearbook with the number of high- and low-skilled workers in each industry provided by census data. In each city’s statistical yearbook, the average wages of workers employed in different industries are counted; in the census data, information about the education and industry of the labor force is provided. Therefore, I could first obtain an individual worker’s wage based on the average wage at the industry-city level where the worker was employed, and then calculate the city-level average wage by skill according to Eqs (1) and (2):

$$w_{jt}^{H}=\frac{1}{H_{jt}}\underset{ind}{\sum }w_{ind,jt}\cdot H_{ind,jt}$$ (1)

$$w_{jt}^{L}=\frac{1}{L_{jt}}\underset{ind}{\sum }w_{ind,jt}\cdot L_{ind,jt}$$ (2)

where *w*_*ind*,*jt*_ is the average wage of workers engaged in industry *ind* in city *j* in year *t*. *H*_*ind*,*jt*_ is the number of high-skilled workers working in industry *ind* in city *j* in year *t*. *H*_*jt*_ is the number of high-skilled workers working in city *j* in year *t*. *$w_{jt}^{H}$* is the average wage of high-skilled workers in city *j* in year *t*. Eq (2) focuses on low-skilled workers, and the specific meaning of each item is similar.

Although wage information is provided in the 2005 census data, in some cities, there is a gap between the wages of high-skilled/low-skilled labor obtained directly from the census data and the wages of high-skilled/low-skilled labor obtained using the weighted method. I used the average wages of employed workers provided by the China Urban Statistical Yearbook as the standard and used the wages calculated by the weighted method after a comprehensive comparison.

However, this method has shortcomings. Since census data is obtained by systematic sampling from raw data, when it is subdivided into *j* city *ind* industry to count the number of workers with different skills, the wage calculations in some cities were abnormal due to limited samples or biased sampling; for example, the average wage of high-skilled workers is lower than the average wage of low-skilled workers, the average wage of high-skilled workers is lower than the average wage of all workers in a city, the average wage of low-skilled workers is higher than the average wage of all workers in a city, and the average wage of high/low-skilled workers in the early years is higher than the average wage of high/low-skilled workers in the later years. Also, data on the average wages of subindustries in some cities are missing for some reason, so the average wage of high/low-skilled workers cannot be calculated. I regarded them as abnormal calculation results. After summarizing, the percentage of anomalous calculations of workers’ average wage by skill is 12%, 15%, and 6% in 2005, 2010, and 2015, respectively. I used Variational Autoencoder (VAE) to recover these outliers.

### 2.3. Recovering city-skill level wages using a VAE

A VAE is a deep generative model that was first proposed by Kingma and Welling (2013) [45]. It is a generative network structure based on a Gaussian mixed model that uses variational Bayesian inference (Goodfellow et al., 2016) [46]. In the fields of economics and finance, due to its powerful data generation capability, VAE is widely used for data synthesis (Koenecke and Varian, 2020) [47], time series forecasting (Jin et al., 2022) [48], big data processing (Sarduie et al., 2020) [49], risk management and control (Arian et al., 2020) [50], stock index tracking (Zhang et al., 2020) [51], education quality improvement (Wang et al., 2021) [52], etc.

Unlike a traditional autoencoder (AE) that describes a latent space by points, a VAE describes the observation of a latent space in the form of a probability distribution. Regularized encoding distribution ensures that it has good characteristics in the latent space, making data generation possible (Blei et al., 2017) [53]. Data processing using a VAE can be divided into four steps: first, the input is encoded as a distribution over a latent space; second, a point in the latent space is sampled from this distribution; third, the sampled point is decoded, and the reconstruction error is calculated; and finally, the reconstruction error is back-propagated through the network (Rezende et al., 2014) [54].

**Fig 1:**
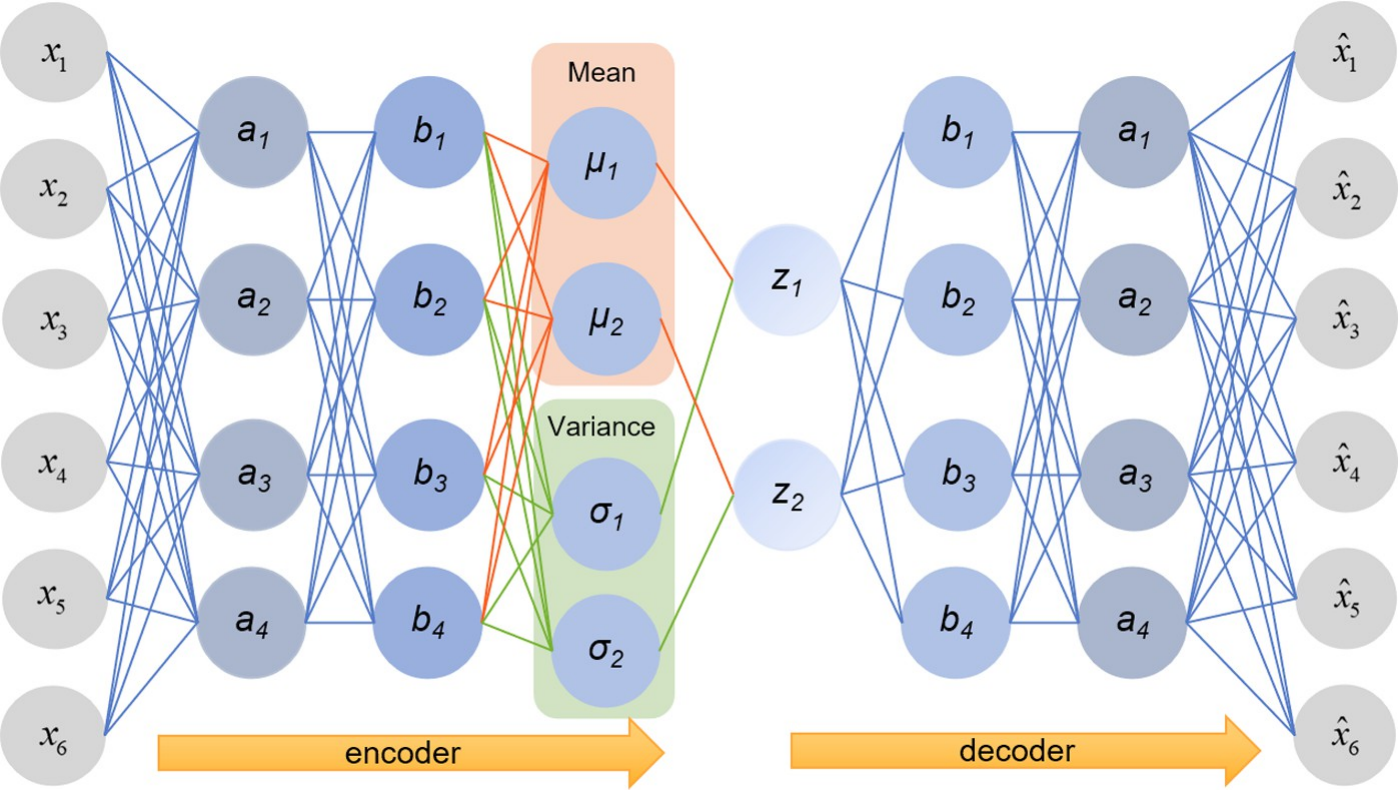
VAE structure.

In practice, the process of generating data can be summarized as follows:

1. Input a data point *x*_*i*_ to the encoder and obtain the parameters of the approximate posterior distribution *q*_*ϕ*_(*z*|*x*_*i*_) obeyed by latent variable *z* through the neural network. It is generally assumed that the posterior distribution obeys a Gaussian distribution, so let the encoder output the parameters *μ*_*i*_ and *σ*_*i*_ (in practice the variance output *log*(*σ*^2^)) of the Gaussian distribution obeyed by *q*_*ϕ*_(*z*|*x*_*i*_).
2. With parameters *μ*_*i*_ and *σ*_*i*_, add the random variable *ε*_*i*_ ~ *N*(0,1) and draw a *z*_*i*_ from the corresponding Gaussian distribution, which represents a class of samples similar to *x*_*i*_.
3. Input a *z*_*i*_ to the decoder, use the decoder to fit the likelihood distribution *P*_*θ*_(*z*|*x*_*i*_), and let the decoder output parameters *μ*_*i*_^*’*^ and *σ*_*i*_^*’*^ of the Gaussian distribution obeyed by *P*_*θ*_(*z*|*x*_*i*_).
4. After obtaining the parameters of the *P*_*θ*_(*z*|*x*_*i*_) distribution, a sample from this distribution is used to generate possible data points $\hat{x_{i}}$. Fig 1 shows the basic structure of a VAE.

According to the central limit theorem, the average wage distribution of type-z workers in year *t* across all cities approximately obeyed a Gaussian distribution, where *z* ∈ {*H*, *L*}; the employed workers’ average wage distribution in year *t* across all cities also approximately obeyed a Gaussian distribution. After normalizing the two, they both approximately obeyed the standard Gaussian distribution. Then, I could make an a priori hypothesis. I assumed that for city *j* in year *t*, the quantile of z-type workers’ average wage in the standardized distribution of the average wage of z-type workers’ average wage across all cities is the same as the quantile of the average wage of the employed workers in city *j* in the standardized distribution of the average wage of the employed workers across all cities.

In this paper, both the input layer and the output layer are 1-dimensional, and I included nine hidden layers, with 60, 120, 72, 24, 1, 24, 72, 120, and 60 nodes, respectively. I used the *LeakyReLU* function as the activation function. The random seeds were set to control the randomness of the results (Nado et al., 2021; Chung et al., 2021) [55, 56], and the learning rate was set to 0.001. According to Kingma and Ba (2014) [57], I used *AdamOptimizer* as the optimizer.

### 2.4. Main variables

I selected data on seventeen urban amenities that endogenously respond to the urban high-skill employment ratio. Wages, rents, land use regulations, land unavailability, and amenities are the main variables used in this paper. Table 1 reports the summary statistics for the main relevant variables.

**Table 1:** Summary statistics.

	Observations	Mean	Standard Deviation	Min.	Max.
(A) Prices					
Ln Low-skill wage	861	7.73142	0.493232	6.269821	8.968838
Ln High-skill wage	861	8.038189	0.462672	6.538978	9.249397
Ln Rent	861	6.001059	0.531416	4.184351	7.915271
(B) Amenities					
Ln Carbon dioxide emissions per 10,000 residents	861	-1.68698	0.744447	-4.15395	1.30798
Ln Hospital beds per 10,000 residents	861	3.942264	0.463776	1.718912	5.18151
Ln Hospitals per 10,000 residents	861	0.562836	0.865874	-5.50896	3.462497
Ln Pm2.5 inhalation per 10,000 residents per cubic meter	852	-0.91587	0.783385	-3.98344	0.771147
Ln Financial institutions per 10,000 residents	864	1.224548	0.610018	-1.31312	3.198557
Ln Taxis per 10,000 residents	861	2.703503	0.794993	0.273243	4.739108
Ln Class road mileage per 10,000 residents	861	4.234676	0.9694	1.306661	6.708812
Ln Bus (electric vehicles) in operation per 10,000 residents	861	1.656306	0.719231	-1.12257	4.107117
Ln Movie theaters per 10,000 residents	861	-2.49606	1.317778	-10.4773	1.525024
Ln Public transport passengers per 10,000 residents	861	4.223144	1.10853	-1.89624	6.777184
Ln Educational expenses per 10,000 residents	861	7.625763	0.926441	4.000581	10.00511
Ln New enterprise registrations per 10,000 residents	861	6.991617	0.922714	3.718998	9.213785
Ln K12 schools per 10,000 residents	861	-0.64002	0.685505	-3.46964	1.76531
Ln Green land area per 10,000 residents	861	3.267595	0.759917	-0.8857	6.078903
Ln College students per 10,000 residents	843	5.641049	0.977496	2.587565	7.820496
Ln Cultural titles per 10,000 residents	861	-4.49395	1.940603	-12.0655	-0.98432
Ln Employment rate	861	-0.12345	0.137057	-1.38629	-.008722
(C) Measures of housing supply elasticity					
Land use regulation	840	1.927328	0.6518	-0.69712	3.905005
Land unavailability	837	0.105803	0.152023	0.000831	0.704345

Note: K12 refers to the education stage from elementary school (over six years old) to high school (under eighteen years old). In this paper, the sum of the number of general elementary schools and general middle schools was used as an indicator of the number of K12 schools. The statistical scope of financial institutions is all financial institutions that have been approved by the China Banking and Insurance Regulatory Commission to obtain a financial license. The statistical scope of cultural titles includes Chinese historical and cultural cities, Chinese historical and cultural towns, Chinese historical and cultural villages, Chinese historical and cultural districts, China’s World Cultural Heritage List, and National Key Cultural Relics Protection Units. The same scenic spot or location can have multiple titles. Employment rate refers to the share of employed workers aged fifteen to sixty-five years in industries other than agriculture in each city. The land use regulation index measures the intensity of policies and regulations that restrict land use for housing development in each city. Following the spirit of Tao (2011) [58], Fan and Mo (2013) [59], I used the ratio of the average sales price of commercial and residential land to the average sales price of industrial land to measure the intensity of land use regulation. The land unavailability index measures the share of land that is unsuitable for housing development due to wetlands, lakes, rivers, and other internal water bodies and slopes exceeding 25% within 30 km of each urban center. The data required for this indicator were calculated using the 30-meter resolution data of the China Multi-Period Land Use Land Cover Change Remote Sensing Monitoring Dataset (CNLUCC) and the ASTER Global Digital Elevation Model dataset.

## 3. Stylized facts

Based on available data, I measured changes in urban skill composition, migration trend, and sorting trend, from which I drew spatial sorting characteristics. I then measured changes in inequality in nominal wages, rents, and real wages between high- and low-skilled workers. From these observations, I documented four stylized facts and drew inferences.

### 3.1. Fact 1: Increasing share of migrants and high-skilled labor

From here forward, I refer to the decennial census and the mini-census simply as “the census”. Using census data from 2000 to 2015, I calculated the share of urban migrants and the share of high-skilled labor among migrants and residents, respectively, for each of the four survey years. Table 2 shows that, from 2000 to 2015, the share of migrants in each city gradually increased, and the growth rate of each five-year period also increased, which means that the share of migrants in each city grew increasingly faster. Simultaneously, the share of high-skilled labor among migrants and residents both increased, with the share of high-skilled labor among migrants growing faster. Its average annual growth rate was 1.65 times higher than the average annual growth rate of residents’ high-skill share. It is obvious that the migration trend gradually increased from 2000 to 2015 across cities. At the same time, the proportion of workers with a bachelor’s degree or above across the country has increased. Also, the proportion of high-skilled workers who choose to live in cities other than their household registration cities is increasing.

**Table 2:** Share of migrants and high-skilled labor from 2000 to 2015.

	2000	2005	2010	2015
Share of migrants	0.058	0.093	0.129	0.243
Share of high-skilled labor (migrants)	0.014	0.048	0.073	0.122
Share of high-skilled labor (residents)	0.038	0.062	0.079	0.104

Note: All working population aged 15 to 65 across the cities was used as the sample, and the range of industry excludes agriculture. The figures were calculated using samples screened from the census dataset based on the definition of migrants by the CMDS.

### 3.2. Fact 2: The sorting trend is increasing, with larger cities having higher wages, higher rents, and a smaller wage gap between high- and low-skilled labor

#### (1) Sorting

**Fig 2:**
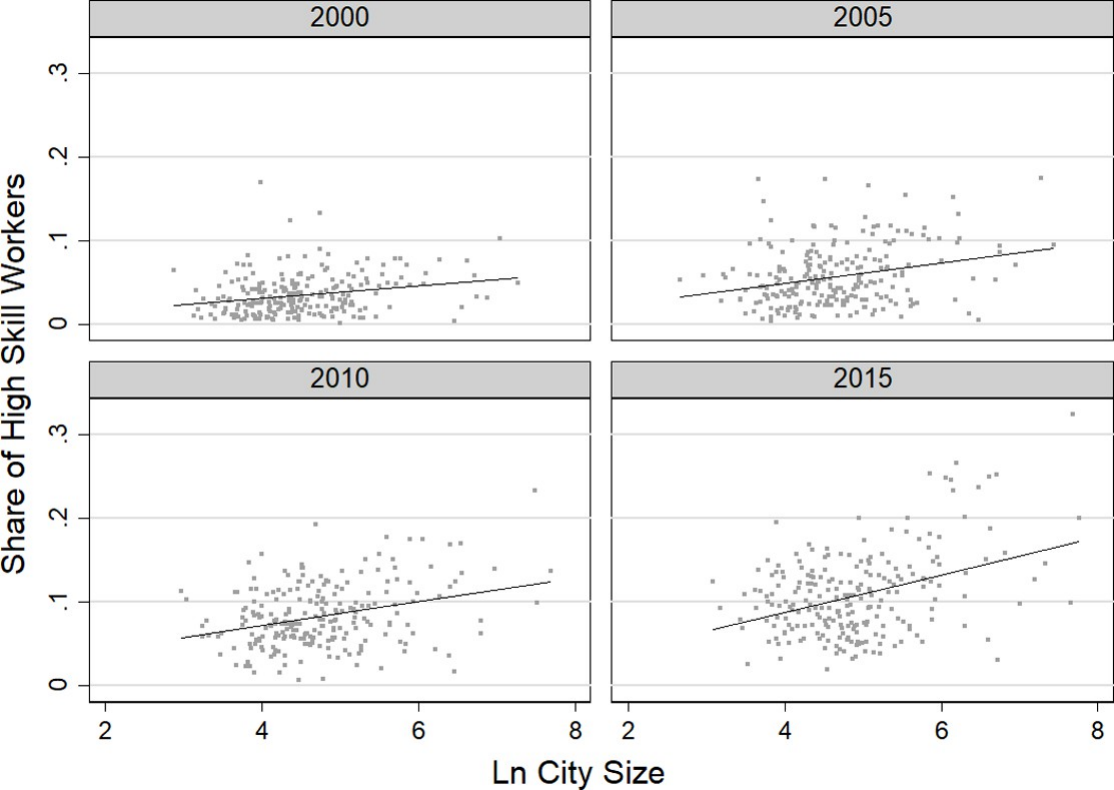
Changes in the spatial sorting trend of migrants from 2000 to 2015.

Fig 2 shows how sorting trend has changed over time. The semi-elasticity in 2015 was 0.0224, indicating that for every 1% increase in city size in that year, the share of high-skilled labor in migrants would increase by 2.24%. From 2000 to 2015, the semi-elasticity of the urban high-skilled labor share with respect to city size nearly tripled, indicating a growing trend of labor sorting across cities.

#### (2) Wage premium

**Fig 3:**
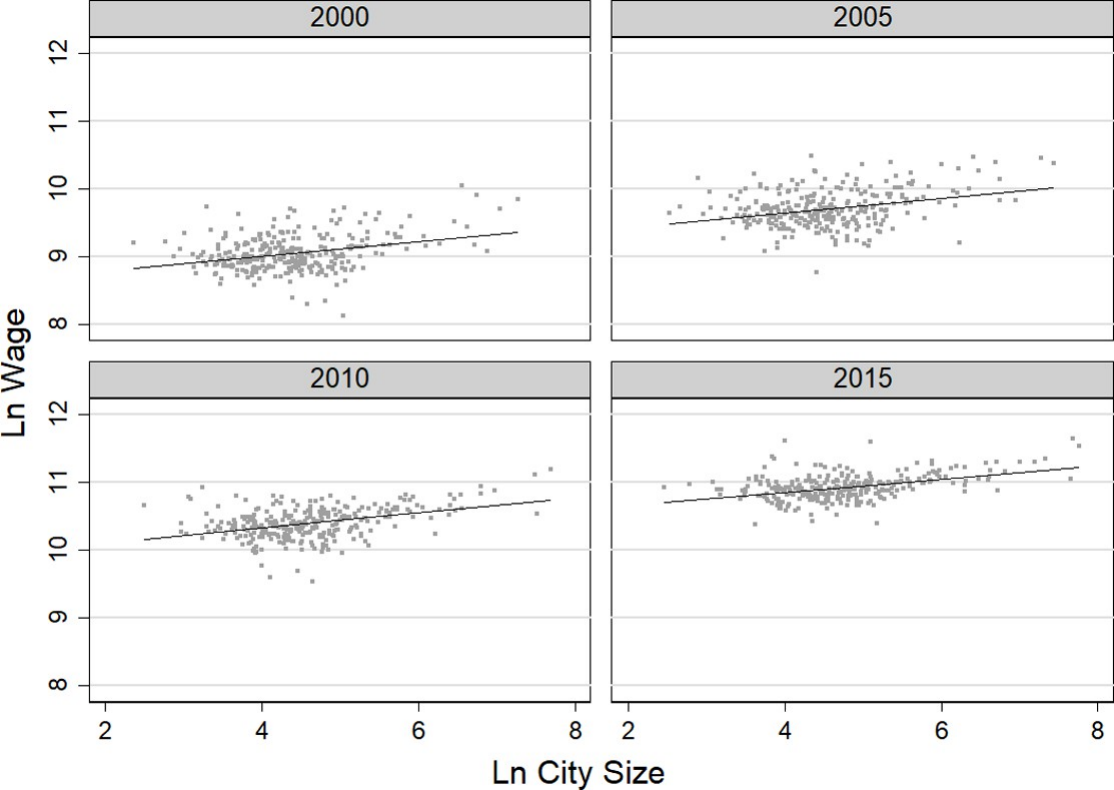
Changes in wage premium of migrants from 2000 to 2015.

Fig 3 shows the urban wage premium, which measures the increase in nominal average wages as city size increases. From 2000 to 2015, the elasticity of the average labor wage with respect to city size remained positive, and the average urban wage premium was 0.123, indicating that the larger a city, the higher the nominal wage; for every 1% increase in city size, the average labor wage increased by about 0.12%.

#### (3) Skill premium

**Fig 4:**
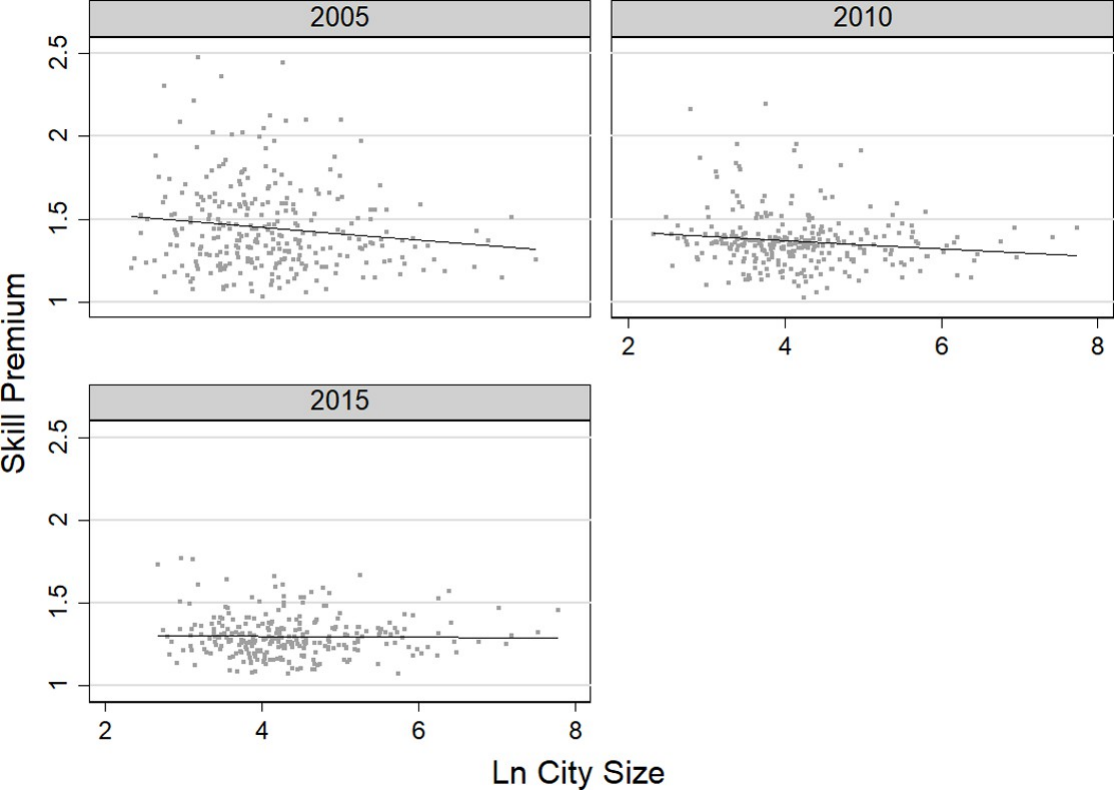
Changes in skill premium of migrants from 2005 to 2015.

Fig 4 shows the urban skill premium, which measures the degree to which the ratio of wages for high-skilled workers compared with low-skilled workers increases as city size increases. Since there were some extreme values in the raw data, I trimmed the data by truncating the extreme values at the top and bottom 0.05% quantiles of the income distribution, respectively. That is to say, nearly 150 extreme values were removed, representing about 1‰ of the total data. The skill premium elasticities were negative for all survey years, indicating that large cities do not imply a greater income gap between high- and low-skilled labor. The skill premium elasticity for 2015 was -0.00168, indicating that for every 1% increase in city size that year, the ratio of wages for high-skilled workers compared with low-skilled workers decreased by about 0.17%.

#### (4) Rent elasticity

**Fig 5:**
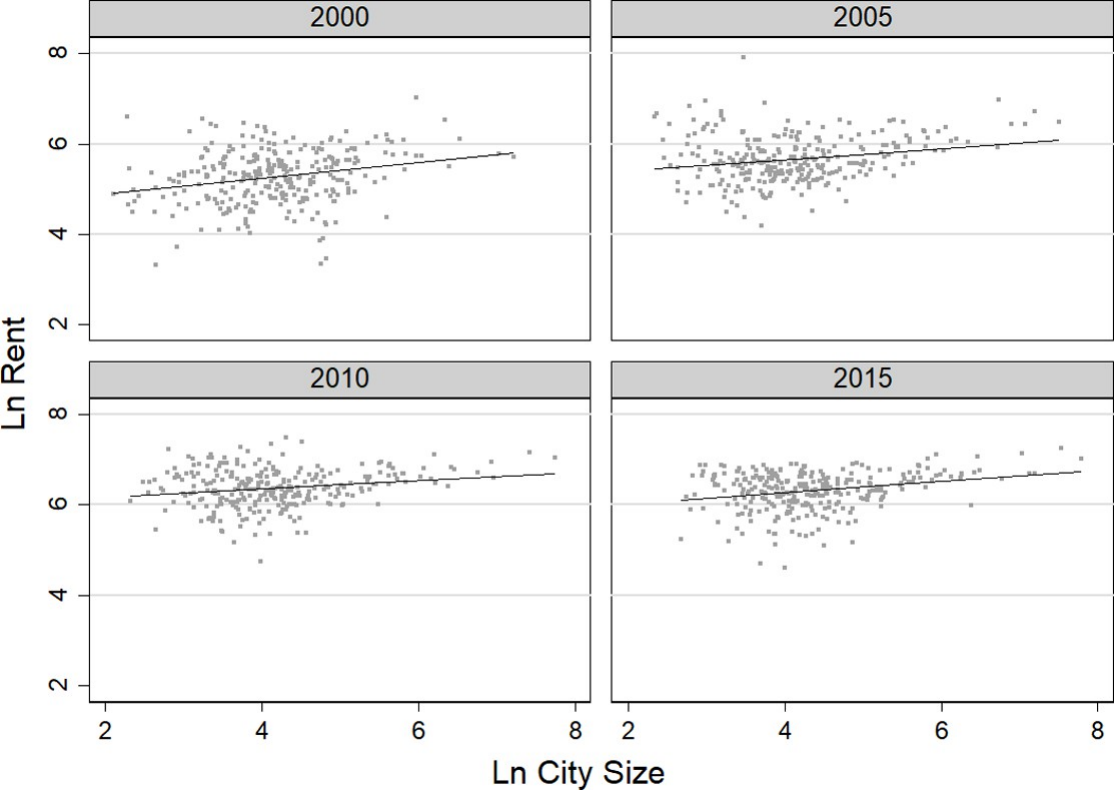
Changes in rent elasticity for migrants from 2000 to 2015.

Fig 5 shows the relationship between rent and city size. The elasticity of rents with respect to city size from 2000 to 2015 was positive, with an average elasticity of 0.127. This shows that the larger the city size, the higher the average rent migrants had to pay. From 2000 to 2015, for every 1% increase in city size, the average rent increased by about 0.13%.

### 3.3. Fact 3: The share of high-skilled labor has increased more in small cities, and the wages have increased more in high-skill cities

**Fig 6:**
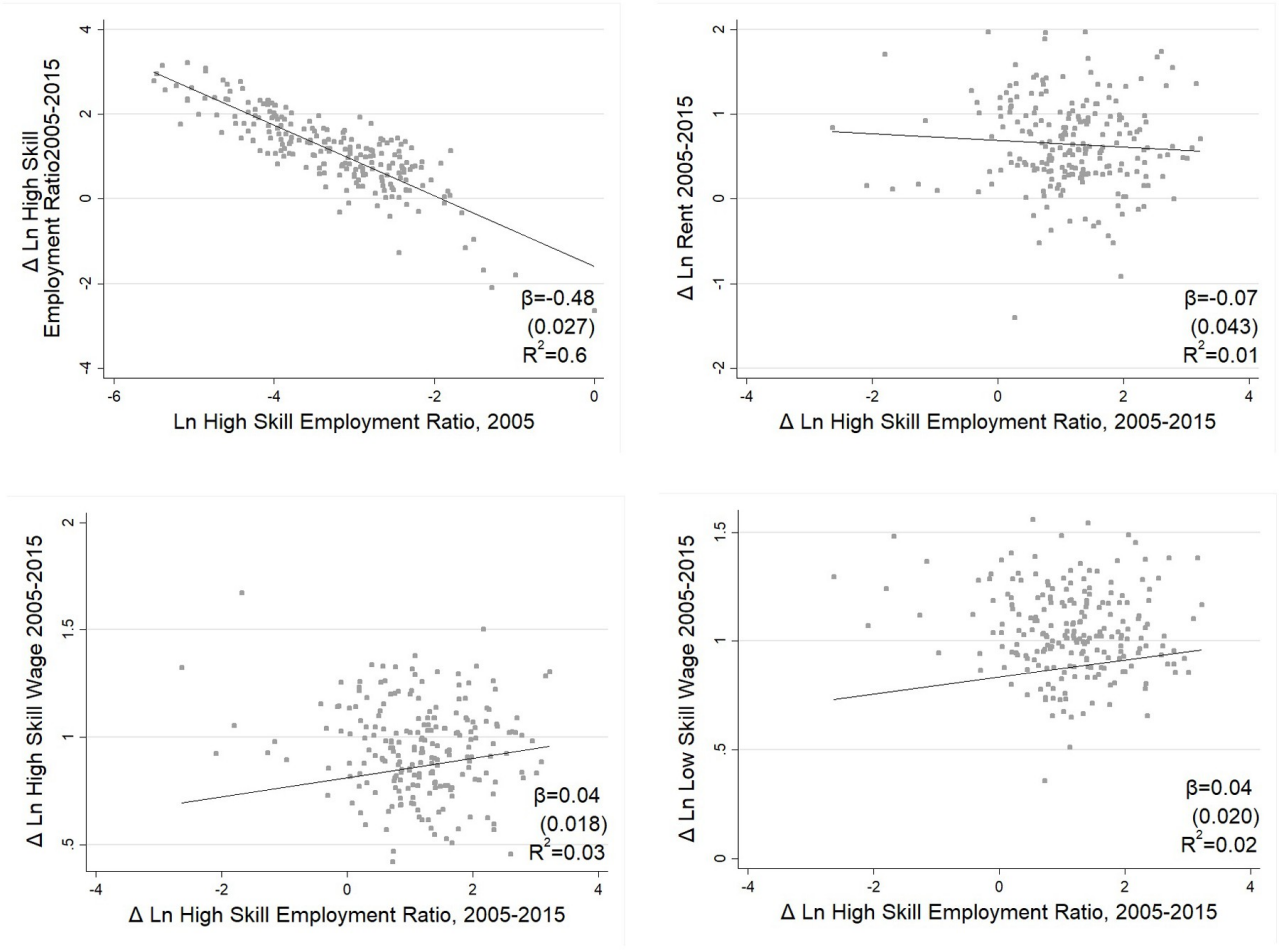
Changes in wages, rents, and high-skill employment ratios from 2005 to 2015.

Moretti (2004), Berry and Glaeser (2005), Shapiro (2006), and Moretti (2012) [60–63] noted that U.S. cities with higher college employment ratios in the base year also experienced larger increases in college employment ratios, a polarization these researches referred to as the “Great Divergence.” For comparison, Fig 6A shows that in China, the change in the high-skill employment ratio in the decade after the base year (2005) was negatively correlated with the high-skill employment ratio in the base year. This implies that the ratio of high-skilled employment in small cities had increased more over the decade, which means there was no “Great Divergence” in China.

Differences in skill mix across cities are strongly correlated with wages and living costs. Fig 6B shows that there is a weak negative correlation between changes in average rents and changes in local high-skill employment ratios over the ten years. Fig 6C and 6D show that there is a positive correlation between the change in high (low) skill wages and the change in high-skill employment ratios with a coefficient of 0.045 (0.039), which means that for every 1% increase in the high-skill employment ratio change, high (low) skill wages would increase by 0.045 (0.039) percentage points. From the stylized facts above, one might ask: Why do wages increase more for all workers in high-skill cities? What is the mechanism? The empirical part of this paper uses structural and reduce-form equations to provide insight into the relationship between high- and low-skilled labor and within high-skilled/low-skilled labor to answer these questions.

### 3.4. Fact 4: The nominal wage gap between high- and low-skilled migrants has narrowed, the rent gap has widened, and the real wage gap has narrowed

**Fig 7:**
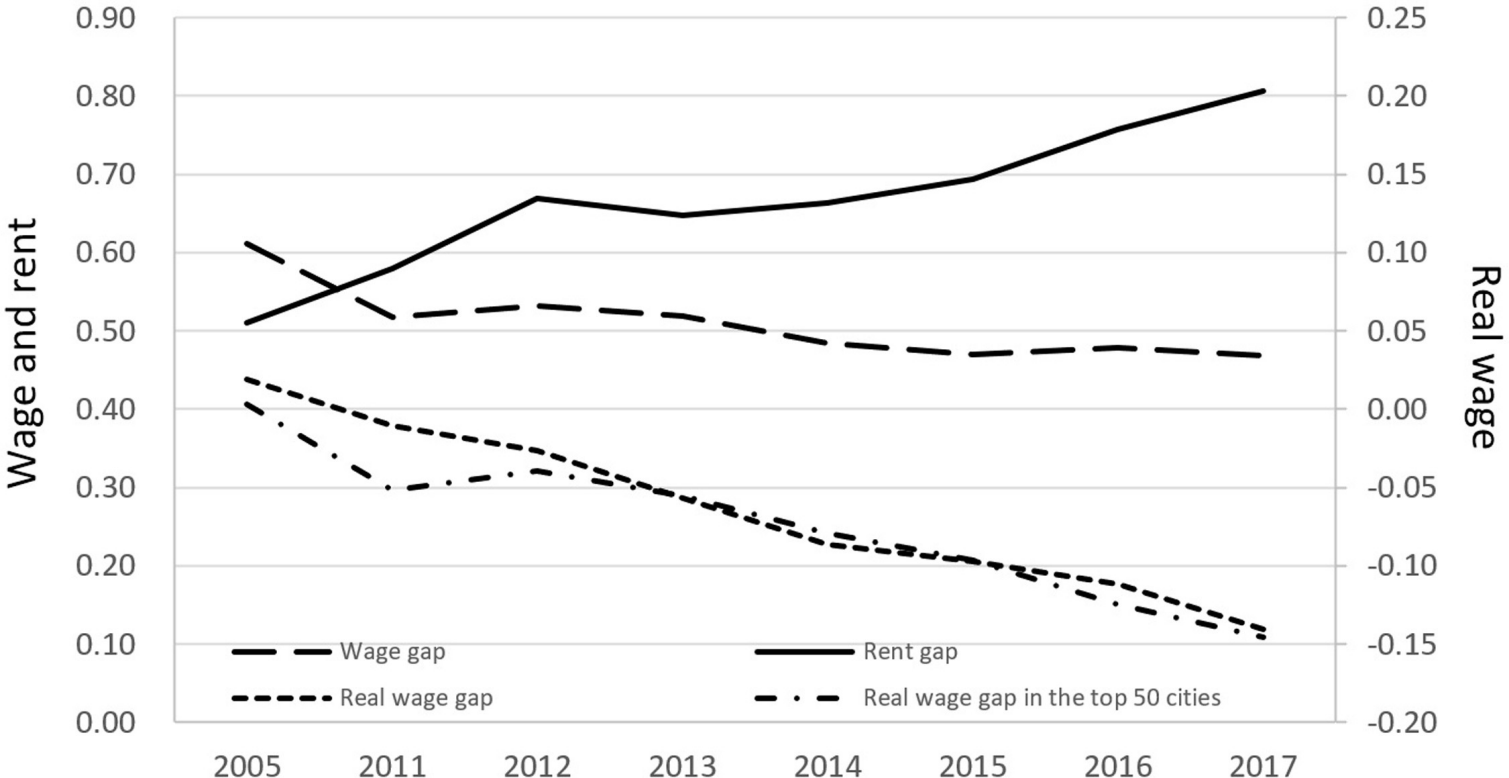
Changes in wages, rents, and real wages of migrants from 2005 to 2015.

In this paper, I used the approach of Mincer (1974) [64] to measure changes in inequality of wages, rents, and real wages caused by the spatial sorting of workers across cities via the elasticities of wages, rents, and real wages with respect to a worker’s duration of education. I used the difference between the elasticity of wages for high-skilled workers and the elasticity of wages for low-skilled workers to represent the wage gap, the difference between the elasticity of rents for high-skilled workers and the elasticity of rents for low-skilled workers to represent the rent gap, and the difference between the elasticity of real wages for high-skilled workers and the elasticity of real wages for low-skilled workers to represent the real wage gap. Fig 7 shows that the wage gap has slowly declined since 2005, and has remained around 0.48 since 2014. The rent gap has generally shown an upward trend since 2005, and this trend has increased significantly since 2015. The real wage gap has generally declined since 2005. The results show that from 2005 to 2015, the domestic wage gap gradually decreased, the rent gap gradually increased, and the real wage gap gradually decreased. During this decade, the nominal wage gap narrowed by 0.141 log points, the rent gap widened by 0.183 log points, and the real wage gap narrowed by 0.116 log points. Converted into percentages, the nominal wage gap narrowed by about 23.08%, and the rent gap widened by about 35.81%. If the year is extended to 2017, the changes in these three indicators remain stable. Taking it a step further, I sorted the cities in descending order of size and selected the top fifty large cities to calculate the real wage gap. I found that, except for 2013 and 2014, the real wage gap calculated using the top fifty large cities was smaller than the real wage gap calculated using all cities. It can be inferred that in most years, the real wage gap in large cities is smaller than the real wage gap in small cities. It can be seen that moving to a large city does not necessarily mean that the real wage gap will widen, so there is another factor besides wages and rents that is driving the increasing trend of sorting across cities.

### 3.5. Summary of stylized facts

This section presents four key facts about migration, housing costs, and changes in income inequality. These facts suggest that, as the economy continues to grow, workers are migrating to live in cities other than their household registration locations, and the proportion of high-skilled migrants is increasing. Migration is characterized by an increasing trend in spatial sorting; living in a large city earns higher wages but also requires paying higher rents, and the wage gap between high and low skills is smaller in large cities than in small ones. The proportion of high-skilled workers in small cities has increased much more than in large cities. Wage growth is higher for all skilled labor in high-skill cities, and income inequality measured by the real wage gap between migrants with different skills is narrowing. A question naturally arises: Since moving to a large city does not necessarily mean that the real wage gap will widen, what factor other than wages and rents is driving the growing trend of worker sorting across cities?

Much of the literature on migration, such as Dudwick (2011), Mourmouras and Rangazas (2013), Xia and Lu (2015), and Liu and Wei (2019) [65–68] make a similar point: the availability of amenities is an important factor that drives labor migration. How do urban amenities affect the location choice of the labor force? Do changes in urban amenities alter the spatial sorting patterns of workers? To clearly describe the mechanisms by which urban amenities affect spatial sorting and to quantify the impact of urban amenities in this process, this paper required causal estimates of labor migration elasticities and the specific characteristics of cities. The impact of changes in the number of high- and low-skilled workers on wages, rents, and amenities depends on the elasticity of the local housing supply, local labor demand, and amenity supply into which I delved. Furthermore, using the utility microfoundation of workers’ city choices, migration elasticities can be mapped onto utility functions, and the estimated parameters can be used to quantify the welfare effects of changes in wages, rents, and amenities. To measure how these supply elasticities and demand elasticities interact and ultimately lead to equilibrium outcomes, I used structural models to explore these questions in depth via techniques such as conditional logit estimation, general moment estimation, and counterfactual simulation.

## 4. Urban spatial equilibrium model

A spatial equilibrium model is presented in this section. The setup of the model follows the main idea of Diamond (2016) [1] and adds to it the feature of migration costs. The model assumes that labor preferences, urban productivity, and urban housing supply are heterogeneous. Local productivity and amenities are set to respond endogenously to the skill set of local workers. This section details the following settings: labor demand, housing supply, labor supply, amenity supply, and how these items together determine spatial equilibrium across cities.

### 4.1. Labor demand

In this paper, subscript *j* is used to represent a city, and subscript d is used to represent a firm. Each city *j* has many homogeneous firms in year *t*. These firms use high-skilled labor *H*_*djt*_, low-skilled labor *L*_*djt*_, and capital *K*_*djt*_ to produce homogeneous tradable goods. The form of the production function is:

$$Y_{djt}=N_{djt}^{\alpha }K_{djt}^{1-\alpha }$$ (3)

$$N_{djt}={\left(\theta_{jt}^{L}L_{djt}^{\rho }+\theta_{jt}^{H}H_{djt}^{\rho }\right)}^{\frac{1}{\rho }}$$ (4)

$$\theta_{jt}^{L}=f_{L}\left(H_{jt},L_{jt}\right)\exp\left(\varepsilon_{jt}^{L}\right)$$ (5)

$$\theta_{jt}^{H}=f_{H}\left(H_{jt},L_{jt}\right)\exp\left(\varepsilon_{jt}^{H}\right)$$ (6)

The total amount of labor *N*_*djt*_ and capital *K*_*djt*_ in the production function are in Cobb-Douglas form. The total amount of labor employed by each firm is denoted as *N*_*djt*_ and consists of high-skilled labor *H*_*djt*_ and low-skilled labor *L*_*djt*_ in the form of imperfect substitution. The elasticity of labor substitution is *1/(1-ρ)*, and the constant parameter *ρ* does not change over time.

The differences in urban production functions are reflected in the heterogeneity of urban productivity. The productivity of high and low skills in each city is measured by $\theta_{jt}^{H}$ and $\theta_{jt}^{L}$, respectively. Eqs (5) and (6) show that local productivity is determined by exogenous and endogenous factors, with the exogenous component being different across cities and determined by $exp\left(\varepsilon_{jt}^{L}\right)$ and $exp\left(\varepsilon_{jt}^{H}\right)$, and the endogenous component being determined by the share of high and low skills in a city. Following the approach of the literature (Moretti, 2011; Carlino and Kerr, 2015) [10, 69], I did not set a specific form of spillover, where the employment of high- and low-skilled labor affects the productivity of high- and low-skilled labor through the functions *f*_*H*_ (*H*_*jt*_, *L*_*jt*_) and *f*_*L*_ (*H*_*jt*_, *L*_*jt*_), respectively.

Assume that there are a large number of firms and that there are no barriers to entry into the market, so the labor market is perfectly competitive, and the wage paid by firms to hire labor is equal to the marginal product of labor. Assume that the capital market is frictionless, the supply of capital is completely elastic, and the price of capital is the same across cities, denoted as *k*_*t*_. The demand for labor and capital by each firm can be written as follows:

$$W_{jt}^{H}=\alpha N_{djt}^{\alpha -\rho }K_{djt}^{1-\alpha }H_{djt}^{\rho -1}f_{H}\left(H_{jt},L_{jt}\right)exp\left(\varepsilon_{jt}^{H}\right)$$ (7)

$$W_{jt}^{L}=\alpha N_{djt}^{\alpha -\rho }K_{djt}^{1-\alpha }L_{djt}^{\rho -1}f_{L}\left(H_{jt},L_{jt}\right)\exp\left(\varepsilon_{jt}^{L}\right)$$ (8)

$$\kappa_{t}=N_{djt}^{\alpha }K_{djt}^{-\alpha }\left(1-\alpha \right)$$ (9)

The production function of firms has constant returns to scale and uses the same production technology, so the firm-level labor demand can be directly translated into the city-level aggregate labor demand. Substituting the equilibrium capital level, the logarithm of labor demand at the city level can be written as follows:

$$w_{jt}^{H}=c_{t}+\left(1-\rho \right)lnN_{jt}+\left(\rho -1\right)lnH_{jt}+ln\left(f_{H}\left(H_{jt},L_{jt}\right)\right)+\varepsilon_{jt}^{H}$$ (10)

$$w_{jt}^{L}=c_{t}+\left(1-\rho \right)lnN_{jt}+\left(\rho -1\right)lnL_{jt}+ln\left(f_{L}\left(H_{jt},L_{jt}\right)\right)+\varepsilon_{jt}^{L}$$ (11)

$$N_{jt}={\left(exp\left(\varepsilon_{jt}^{H}\right)f_{H}\left(H_{jt},L_{jt}\right)H_{jt}^{\rho }+exp\left(\varepsilon_{jt}^{L}\right)f_{L}\left(H_{jt},L_{jt}\right)L_{jt}^{\rho }\right)}^{\frac{1}{\rho }}$$ (12)

$$c_{t}=ln\left(\alpha {\left(\frac{1-\alpha }{\kappa_{t}}\right)}^{\frac{1-\alpha }{\alpha }}\right)$$ (13)

The above equations show that labor supply affects wages via imperfect substitution of high- and low-skilled labor within firms (controlled by *ρ*) and changes in urban productivity (controlled by *f*_*H*_ (*H*_*jt*_, *L*_*jt*_), *f*_*L*_ (*H*_*jt*_, *L*_*jt*_)). In estimating these equations, the only way to distinguish between the effects of endogenous productivity and imperfect labor substitution on wages is to parameterize *f*_*H*_ (*H*_*jt*_, *L*_*jt*_), *f*_*L*_ (*H*_*jt*_, *L*_*jt*_) through a strong assumption. Instead of imposing parametric constraints, the labor demand equation can be written as an unknown function of employment level (*H*_*jt*_, *L*_*jt*_) and exogenous productivity $\left(\varepsilon_{jt}^{H},\varepsilon_{jt}^{L}\right)$:

$$w_{jt}^{H}=g_{H}\left(H_{jt},L_{jt}\right)+\varepsilon_{jt}^{H}$$ (14)

$$w_{jt}^{L}=g_{L}\left(H_{jt},L_{jt}\right)+\varepsilon_{jt}^{L}$$ (15)

where *g*_*H*_(*H*_*jt*_, *L*_*jt*_) and *g*_*L*_(*H*_*jt*_, *L*_*jt*_) represent the combined effects of imperfect labor substitution and endogenous productivity. Using log-linearized total labor demand to estimate these functions, the equations can be rewritten as follows:

$$w_{jt}^{H}=\gamma_{HH}lnH_{jt}+\gamma_{HL}lnL_{jt}+\varepsilon_{jt}^{H}$$ (16)

$$w_{jt}^{L}=\gamma_{LH}lnH_{jt}+\gamma_{LL}lnL_{jt}+\varepsilon_{jt}^{L}$$ (17)

### 4.2. Urban labor supply

Let the subscript *i* denote workers in each household, and assume that these workers choose to live in a city that offers them the most attractive wages, local good prices, and amenities. The wage of high-skilled labor differs from that of low-skilled labor in each city. The wage earned by a worker with education level *edu* who resides in city *j* in year *t* and who inelastically provides one unit of labor is recorded as $W_{jt}^{edu}$.

The worker consumes the local good *M* and the national tradable good *O*, the price of the local good is denoted as *R*_*jt*_, and the price of the tradable good is denoted as *P*_*t*_. Also, the worker derives utility from the city’s amenity *A*_*jt*_. The worker has a Cobb-Douglas preference for local and tradable goods, and he or she maximizes his or her utility subject to budget constraints:

$$\underset{M,O}{max}ln\left(M^{\zeta }\right)+ln\left(O^{1-\zeta }\right)+s_{i}\left(A_{jt}\right)$$ (18)

$$s.t.P_{t}O+R_{jt}M\leq W_{jt}^{edu}$$ (19)

The relative preference of workers for local and tradable goods is controlled by *ζ*, where 0 ≤ *ζ*_*i*_ ≤ 1. The optimal utility function of a worker can be represented by the indirect utility function of living in city *j*. If a worker resides in city *j* in year *t*, his utility *V*_*ijt*_ is:

$$V_{ijt}=ln\left(\frac{W_{jt}^{edu}}{P_{t}}\right)-\zeta ln\left(\frac{R_{jt}}{P_{t}}\right)+s_{i}\left(A_{jt}\right)$$ (20)

$$=w_{jt}^{edu}-\zeta r_{jt}+s_{i}\left(A_{jt}\right)$$ (21)

The prices of tradable goods were measured in 2015 prices using the CPI index. From the worker’s optimal utility function, his or her local good demand *HD*_*ijt*_ can be deduced as follows:

$$HD_{ijt}=\frac{\zeta W_{jt}^{edu}}{R_{jt}}$$ (22)

Workers’ preferences for local non-market amenities are heterogeneous. This paper follows Diamond’s (2016) [1] definition of amenity, which refers to all characteristics that can affect the attractiveness of a city, other than local wages and prices. This includes local social security programs, urban infrastructure, public services, and some natural conditions, such as rainfall. In this paper, the vector $x_{jt}^{A}$ was used to represent the exogenous amenities of city *j* in year *t*. It did not respond to the endogenous variables in the model. Workers made a single-index evaluation value *a*_*jt*_ for the urban amenity bundle. The key feature of *a*_*jt*_ is that it responds endogenously to the share of high- and low-skilled labor in a city.

Function *s*_*i*_(*A*_*jt*_) maps the urban amenity vector *A*_*jt*_ to the utility value of a worker. The estimated value of amenity *A*_*jt*_ for worker *i* is:

$$s_{i}\left(A_{jt}\right)=a_{jt}\beta_{i}^{a}+x_{jt}^{A}\beta_{i}^{x}+\beta_{i}^{prov}x_{j}^{prov}+\beta_{i}^{region}x_{j}^{region}+\sigma_{i}\varepsilon_{ijt}$$ (23)

$$\beta_{i}^{x}=\beta^{x}z_{i}$$ (24)

$$\beta_{i}^{a}=\beta^{a}z_{i}$$ (25)

$$\beta_{i}^{prov}=prov_{i}\beta^{prov}z_{i}$$ (26)

$$\beta_{i}^{region}=reg_{i}\beta^{region}z_{i}$$ (27)

$$\sigma_{i}=\beta^{\sigma }z_{i}$$ (28)

$\beta_{i}^{prov}$ and $\beta_{i}^{region}$ measure the utility value of worker *i* living in a city in the province to which his or her household registration belongs and a city in the region to which his or her household registration belongs, respectively.

According to the “National Standard Citizen ID Card Number of the People’s Republic of China (GB11643-1999)” and the “Administrative Region Code of the People’s Republic of China (GB/T 2260–2007)”, the regions to which cities belong are classified according to the first two regional codes of the national ID number. Taiwan, Hong Kong, and Macau are not included, and the specific correspondence is as follows: North (1): Beijing (11), Tianjin (12), Hebei (13), Shanxi (14), and Inner Mongolia (15); Northeast (2): Liaoning (21), Jilin (22), and Heilongjiang (23); East (3): Shanghai (31), Jiangsu (32), Zhejiang (33), Anhui (34), Fujian (35), Jiangxi (36), and Shandong (37); Central (4): Henan (41), Hubei (42), and Hunan (43); Southwest (5): Sichuan (51), Guizhou (52), Yunnan (53), Tibet (54), and Chongqing (50); Northwest (6): Shaanxi (61), Gansu (62), Qinghai (63), Ningxia (64), and Xinjiang (65); South (7): Guangdong (44), Guangxi (45), and Hainan (46).

The marginal utilities of worker *i* for exogenous amenities $\beta_{i}^{x}$, endogenous amenities $\beta_{i}^{a}$, and household registration amenities $\left(\beta_{i}^{prov},\beta_{i}^{region}\right)$ are all functions of worker’s demographic grouping *z*_*i*_. *z*_*i*_ is a 2 × 1 dummy variable vector, which represents the skill level of the labor and whether the labor is a cross-provincial migrant. The coefficients (*β*^*x*^, *β*^*a*^, *β*^*prov*^, *β*^*region*^, and *β*^*σ*^) are all 1 × 2 vectors that measure the utility value of urban characteristics for a given demographic group. $x_{j}^{prov}$ is a 1 × 30 binary vector that takes the value of 1 if the city in which worker *i* lives belongs to province k. Similarly, $x_{j}^{region}$ is defined as a 1 × 7 binary vector that takes the value of 1 if the city in which worker *i* lives belongs to region *m*. *prov*_*i*_ is a 30 × 1 binary vector that takes the value of 1 if worker *i*’s household registration belongs to a province. *reg*_*i*_ is a 7 × 1 binary vector that takes the value of 1 if worker *i*’s household registration place belongs to a region. Each worker also has an individual heterogeneous preference for urban amenities, measured by *ε*_*ijt*_. *ε*_*ijt*_ obeys a type-I extreme value distribution. The variance of workers’ heterogeneous preferences for each city varies across demographic groups.

I normalized the utility function by dividing the utility of each worker by *β*^*σ*^ *z*_*i*_. The indirect utility of worker *i* in city *j* is:

$$V_{ijt}=\left(w_{jt}^{edu}-\zeta r_{jt}\right)\beta^{w}z_{i}+a_{jt}\beta_{i}^{a}+x_{jt}^{A}\beta_{i}^{x}+\beta_{i}^{prov}x_{j}^{prov}+\beta_{i}^{region}x_{j}^{region}+\varepsilon_{ijt}$$ (29)

For a given city, differences in preferences across workers of the same demographic group *z* are caused by workers’ household registration provinces and their household registration regions (*prov*_*i*_, *reg*_*i*_) as well as their heterogeneous preferences for the city, *ε*_*ijt*_. I defined the utility component of city *j* that is common to all type-z workers as $\delta_{jt}^{z}$:

$$\delta_{jt}^{z}=\left(w_{jt}^{edu}-\zeta r_{jt}\right)\beta^{w}z+a_{jt}\beta^{a}z+x_{jt}^{A}\beta^{x}z$$ (30)

The indirect utility function can be rewritten as follows:

$$V_{ijt}=\delta_{jt}^{z}+x_{j}^{prov}prov_{i}\beta^{prov}z_{i}+x_{j}^{region}reg_{i}\beta^{region}z_{i}+\varepsilon_{ijt}$$ (31)

This setup is consistent with the conditional logit model (McFadden, 1973) [4]. The difference in the total number of type-z workers across cities represents the difference in the average utility estimates of workers for these cities. The expected total population of city *j* is equal to the probability of each worker living in this city, summed over the entire population. The probability that worker *i* will choose to live in city *j* is:

$$Pr(V_{ijt}>V_{i-jt})=\frac{exp\left(\delta_{jt}^{z_{i}}+x_{j}^{prov}prov_{i}\beta^{prov}z_{i}+x_{j}^{region}reg_{i}\beta^{region}z_{i}\right)}{\sum_{k}^{J}exp\left(\delta_{kt}^{z_{i}}+x_{k}^{prov}prov_{i}\beta^{prov}z_{i}+x_{k}^{region}reg_{i}\beta^{region}z_{i}\right)}$$ (32)

Therefore, the total number of high- and low-skilled workers in city *j* is:

$$H_{jt}=\underset{i\in {\mathbb{C}}_{H_{t}}}{\sum }\frac{exp\left(\delta_{jt}^{z_{i}}+x_{j}^{prov}prov_{i}\beta^{prov}z_{i}+x_{j}^{region}reg_{i}\beta^{region}z_{i}\right)}{\sum_{k}^{J}exp\left(\delta_{kt}^{z_{i}}+x_{k}^{prov}prov_{i}\beta^{prov}z_{i}+x_{k}^{region}reg_{i}\beta^{region}z_{i}\right)}$$ (33)

$$L_{jt}=\underset{i\in {\mathbb{C}}_{L_{t}}}{\sum }\frac{exp\left(\delta_{jt}^{z_{i}}+x_{j}^{prov}prov_{i}\beta^{prov}z_{i}+x_{j}^{region}reg_{i}\beta^{region}z_{i}\right)}{\sum_{k}^{J}exp\left(\delta_{kt}^{z_{i}}+x_{k}^{prov}prov_{i}\beta^{prov}z_{i}+x_{k}^{region}reg_{i}\beta^{region}z_{i}\right)}$$ (34)

where ${\mathbb{C}}_{H_{t}}$ and ${\mathbb{C}}_{L_{t}}$ represent the set of high- and low-skilled workers in the country, respectively.

### 4.3. Housing supply

The local good prices *R*_*jt*_ are determined when the housing market is in equilibrium. The local price level represents the prices of local housing and local composite goods, such as groceries and local services. The price of local composite goods is also affected by local housing prices. The inputs used to build housing include construction materials and land. In each city, a developer is the representative of local landowners. The developer is a price taker and sells homogeneous housing at marginal production costs.


$$P_{jt}^{house}=MC\left(CC_{jt},LC_{jt}\right)$$ (35)

Local construction costs *CC*_*jt*_ and local land costs *LC*_*jt*_ are mapped to the marginal cost of building a house by the function *MC*(*CC*_*jt*_, *LC*_*jt*_). There is no uncertainty, and the price is equal to the present value of rent in steady-state equilibrium. Local rents can be written as follows:

$$R_{jt}=\iota_{t}\times MC\left(CC_{jt},LC_{jt}\right)$$ (36)

where *ι*_*t*_ is the interest rate. Houses are owned by absentee landowners, who rent them to local residents. Land cost *LC*_*jt*_ is a function of the aggregate demand for local goods. Eq (22) shows that households increase their demand for local goods when wages rise or local good prices fall. A large number of migrants also increase the demand for housing. Parameterizing the logarithmic housing supply equation, I can obtain:

$$r_{jt}=\ln\left(R_{jt}\right)=\ln\left(\iota_{t}\right)+\ln\left(CC_{jt}\right)+\gamma_{j}\ln\left(HD_{jt}\right)$$ (37)

$$\gamma_{j}=\gamma +\gamma^{geo}exp\left(x_{j}^{geo}\right)+\gamma^{regulation}exp\left(x_{j}^{regulation}\right)$$ (38)

$$HD_{jt}=L_{jt}\frac{\zeta W_{jt}^{L}}{R_{jt}}+H_{jt}\frac{\zeta W_{jt}^{H}}{R_{jt}}$$ (39)

where *HD*_*jt*_ is the total demand for local goods in city *j* in year *t*. The elasticity of rent with respect to local good demand varies across cities, measured by $\gamma_{j}.\;x_{j}^{geo}$ measures the share of land within 30 km of each urban center that is undevelopable due to slopes exceeding 25% and inland water bodies such as wetlands, lakes, rivers, etc. *γ*^*geo*^ measures how changes in $exp\left(x_{j}^{geo}\right)$ affect the inverse elasticity of housing supply *γ*_*j*_. Local land use regulations have a similar effect via policies that restrict housing development. Smaller values of the land use regulation indicator imply more permissive policies toward real estate development. *γ*^*regulation*^ measures how changes in $exp\left(x_{j}^{regulation}\right)$ affect the inverse elasticity of housing supply *γ*_*j*_. *γ* measures the elasticity of basic housing supply elasticity when a city has no land use regulatory policies and no geographic constraints that limit housing development.

### 4.4. Amenity supply

In this paper, I used $x_{jt}^{A}$ to denote exogenous amenities and *a*_*jt*_ to denote amenities that respond endogenously to the type of labor that lives in a city. I allowed the endogenous amenity index to be determined by the urban high-skill employment ratio $\frac{H_{jt}}{L_{jt}}$:

$$a_{jt}=\gamma^{a}ln\left(\frac{H_{jt}}{L_{jt}}\right)+\varepsilon_{jt}^{a}$$ (40)

where *γ*^*a*^ is the elasticity of amenity supply and $\varepsilon_{jt}^{a}$ is the exogenous component of the amenity index *a*_*jt*_. All amenities in a city are represented by the vector *A*_*jt*_:

$$A_{jt}=\left(x_{jt}^{A},x_{j}^{prov},x_{j}^{region},a_{jt}\right)$$ (41)

### 4.5. Equilibrium

The equilibrium of the model is determined by wages, rents, amenity levels $\left(w_{t}^{L*},w_{t}^{H*},r_{t}^{*},\frac{H_{jt}^{*}}{L_{jt}^{*}}\right)$, and population $\left(H_{jt}^{*},L_{jt}^{*}\right)$; therefore, high-skilled labor demand equals high-skilled labor supply:

$$H_{jt}^{*}=\underset{i\in {\mathbb{C}}_{H_{t}}}{\sum }\frac{exp\left(\delta_{jt}^{z_{i}}+x_{j}^{prov}prov_{i}\beta^{prov}z_{i}+x_{j}^{region}reg_{i}\beta^{region}z_{i}\right)}{\sum_{k}^{J}exp\left(\delta_{kt}^{z_{i}}+x_{k}^{prov}prov_{i}\beta^{prov}z_{i}+x_{k}^{region}reg_{i}\beta^{region}z_{i}\right)}$$ (42)

$$w_{jt}^{H*}=\gamma_{HH}lnH_{jt}^{*}+\gamma_{HL}lnL_{jt}^{*}+\varepsilon_{jt}^{H}$$ (43)

low-skilled labor demand equals low-skilled labor supply:

$$L_{jt}^{*}=\underset{i\in {\mathbb{C}}_{L_{t}}}{\sum }\frac{exp\left(\delta_{jt}^{z_{i}}+x_{j}^{prov}prov_{i}\beta^{prov}z_{i}+x_{j}^{region}reg_{i}\beta^{region}z_{i}\right)}{\sum_{k}^{J}exp\left(\delta_{kt}^{z_{i}}+x_{k}^{prov}prov_{i}\beta^{prov}z_{i}+x_{k}^{region}reg_{i}\beta^{region}z_{i}\right)}$$ (44)

$$w_{jt}^{L*}=\gamma_{LH}lnH_{jt}^{*}+\gamma_{LL}lnL_{jt}^{*}+\varepsilon_{jt}^{L}$$ (45)

housing demand equals housing supply:

$$r_{jt}^{*}=ln\left(\iota_{t}\right)+ln\left(CC_{jt}\right)+\gamma_{j}ln\left(HD_{jt}^{*}\right)$$ (46)

$$HD_{jt}^{*}=L_{jt}^{*}\frac{\zeta exp\left(w_{jt}^{L*}\right)}{exp\left(r_{jt}^{*}\right)}+H_{jt}^{*}\frac{\zeta exp\left(w_{jt}^{H*}\right)}{exp\left(r_{jt}^{*}\right)}$$ (47)

endogenous amenity demand equals the endogenous amenity supply:

$$a_{jt}^{*}=\gamma^{a}ln\left(\frac{H_{jt}^{*}}{L_{jt}^{*}}\right)+\varepsilon_{jt}^{a}$$ (48)

$$\delta_{jt}^{z}=\left(w_{jt}^{edu*}-\zeta r_{jt}^{*}\right)\beta^{w}z+a_{jt}^{*}\beta^{a}z+x_{jt}^{A}\beta^{x}z$$ (49)

## 5. Model estimation

In this section, I constructed the endogenous amenity index *a*_*jt*_ and established the instrumental variables needed to solve the endogeneity problem.

### 5.1. Endogenous amenity index

A city’s amenity index should ideally capture the full range of amenities that endogenously respond to the city’s skill mix. To measure urban amenities as broadly and comprehensively as possible, I collected data on seventeen different amenities and classified them into seven categories: financial institutions, transportation infrastructure, education quality, job market, cultural heritage, natural environment, and health care.

This paper used AE to extract a single (one-dimensional) amenity index *a*_*jt*_ for each city. Some amenity categories have more data sources. Since dimensionality reduction of high-dimensional data puts more weight on the amenity categories with more data sources, I first created an amenity category index using the data within each category and then used all amenity category indices to create an overall amenity index, as detailed in Table 3.

**Table 3:** Variable description of urban amenities.

Index	Details
Traffic Index	Taxis per 10,000 residents, Bus (electric vehicles) in operation per 10,000 residents, Public transport passengers per 10,000 residents, Class road mileage per 10,000 residents
Education Index	K12 schools per 10,000 residents, College students per 10,000 residents, Educational expenses per 10,000 residents
Employment Index	New enterprise registrations per 10,000 residents, Employment rate
Cultural Index	Movie theaters per 10,000 residents, Cultural titles per 10,000 residents
Environmental Index	Carbon dioxide emissions per 10,000 residents, Pm2.5 inhalation per 10,000 residents per cubic meter, Green land area per 10,000 residents
Medical Index	Hospitals per 10,000 residents, Hospital beds per 10,000 residents
Financial Index	Financial institutions per 10,000 residents

The most commonly used dimensionality reduction method in the literature (Lipscomb and Farmer, 2005; Huang et al., 2015; Diamond, 2016) [1, 70, 71] is principal component analysis (PCA). PCA is a simplified version of an unsupervised learning neural network. Since it uses only a single-layer neural network to learn simple linear variations (Wang and Xia, 1997) [72], its learning capability is very limited. PCA is not ideal for the dimensionality reduction of complex data because, in reality, there are many nonlinear relationships between high-dimensional data features, and linear projection is no longer applicable, requiring the use of some nonlinear dimensionality reduction methods (Schölkopf et al., 1998) [73]. If the single-layer neural network is transformed into a multi-layer neural network, the linear activation function is replaced by a nonlinear activation function, and the irrelevant constraints between the dimensions of the transformed data are removed, then the PCA is converted into an AE with a more powerful learning capability.

### 5.2. Bartik labor demand shock

When the explanatory variables were endogenous, I used the Bartik instrumental variable, which is commonly used in the literature, to solve the coherent estimated coefficients of the explanatory variables. Changes in industry productivity levels within each city are a component of changes in urban productivity (Bartik, 1991) [6]. According to the different industry compositions of high- and low-skilled labor, changes in industry productivity will have different effects on a city’s local high- and low-skill productivity. I measured exogenous local productivity changes through the interaction between cross-sectional differences in industry employment composition and changes in high- and low-skill wages across industries in the country. Accordingly, this paper defines the Bartik shock for high- and low-skilled labor as follows:

$$\Delta B_{jt}^{H}=\underset{ind}{\sum }\left(w_{ind,-j,t}^{H}-w_{ind,-j,2005}^{H}\right)\frac{H_{ind,j,2005}}{H_{j,2005}}$$ (50)

$$\Delta B_{jt}^{L}=\underset{ind}{\sum }\left(w_{ind,-j,t}^{L}-w_{ind,-j,2005}^{L}\right)\frac{L_{ind,j,2005}}{L_{j,2005}}$$ (51)

where $w_{ind,-j,t}^{H}$ and $w_{ind,-j,t}^{L}$ represent the logarithmic average wages of high- and low-skilled labor in industry *ind* in year *t*, respectively, excluding the labor force in city *j*. *H*_*ind*,*j*,2005_ and *L*_*ind*,*j*,2005_ represent the number of high- and low-skilled labor employed in industry *ind* in city *j* in 2005, respectively.

These Bartik labor demand shocks are part of a city’s exogenous productivity changes over time. Specifically, the exogenous high- and low-skill productivity changes in Eqs (16) and (17) can be written as follows:

$$\Delta \varepsilon_{jt}^{H}=\gamma_{BHH}\Delta B_{jt}^{H}+\gamma_{BHL}\Delta B_{jt}^{L}+\Delta {\tilde{\varepsilon }}_{jt}^{H}$$ (52)

$$\Delta \varepsilon_{jt}^{L}=\gamma_{BLH}\Delta B_{jt}^{H}+\gamma_{BLL}\Delta B_{jt}^{L}+\Delta {\tilde{\varepsilon }}_{jt}^{L}$$ (53)

where ($\Delta \varepsilon_{jt}^{H},\Delta \varepsilon_{jt}^{L}$) is the exogenous productivity change of high and low skills in city *j* in year *t* relative to the base year (2005). (*γ*_*BHH*_, *γ*_*BHL*_, *γ*_*BLH*_, *γ*_*BLL*_) are parameters mapped from $\Delta \varepsilon_{jt}^{H}$ and $\Delta \varepsilon_{jt}^{L}$ to $\Delta B_{jt}^{H}$ and $\Delta B_{jt}^{L}.\;\Delta {\tilde{\varepsilon }}_{jt}^{H}$ and $\Delta {\tilde{\varepsilon }}_{jt}^{L}$ are exogenous local productivity changes uncorrelated with Bartik local labor demand shock.

### 5.3. Labor demand

The amount of labor is a function of local productivity and wages. Differentiating a city’s wage from its base year level yields the following:

$$\Delta w_{jt}^{H}=\gamma_{HH}\Delta lnH_{jt}+\gamma_{HL}\Delta lnL_{jt}+\Delta \varepsilon_{jt}^{H}$$ (54)

$$\Delta w_{jt}^{L}=\gamma_{LH}\Delta lnH_{jt}+\gamma_{LL}\Delta lnL_{jt}+\Delta \varepsilon_{jt}^{L}$$ (55)

Substituting Bartik labor demand shock into the labor demand equation, I got:

$$\Delta w_{jt}^{H}=\gamma_{HH}\Delta lnH_{jt}+\gamma_{HL}\Delta lnL_{jt}+\gamma_{BHH}\Delta B_{jt}^{H}+\gamma_{BHL}\Delta B_{jt}^{L}+\Delta {\tilde{\varepsilon }}_{jt}^{H}$$ (56)

$$\Delta w_{jt}^{L}=\gamma_{LH}\Delta lnH_{jt}+\gamma_{LL}\Delta lnL_{jt}+\gamma_{BLH}\Delta B_{jt}^{H}+\gamma_{BLL}\Delta B_{jt}^{L}+\Delta {\tilde{\varepsilon }}_{jt}^{L}$$ (57)

The direct effect of the Bartik shock is to shift the local labor demand curve, directly affecting local wages.

### 5.4. Housing supply

The change in the housing supply curve after 2005 is as follows:

$$\Delta r_{jt}=\Delta ln\left(\iota_{t}\right)+\Delta ln\left(CC_{jt}\right)+(\gamma +\gamma^{geo}exp\left(x_{j}^{geo}\right)+\gamma^{regulation}exp\left(x_{j}^{regulation}\right))\Delta ln\left(HD_{jt}\right)$$ (58)

$$HD_{jt}=L_{jt}\frac{\zeta W_{jt}^{L}}{R_{jt}}+H_{jt}\frac{\zeta W_{jt}^{H}}{R_{jt}}$$ (59)

### 5.5. Labor supply

The indirect utility of labor *i* with demographic grouping *z*_*i*_ in city *j* is:

$$V_{ijt}=\delta_{jt}^{z}+x_{j}^{prov}prov_{i}\beta^{prov}z_{i}+x_{j}^{region}reg_{i}\beta^{region}z_{i}+\varepsilon_{ijt}$$ (60)

$$\delta_{jt}^{z_{i}}=\left(w_{jt}^{edu}-\zeta r_{jt}\right)\beta^{w}z_{i}+a_{jt}\beta^{a}z_{i}+x_{jt}^{A}\beta^{x}z_{i}$$ (61)

I used a two-step estimation method similar like Berry et al. (2004) [5] to estimate labor’s preference for cities. First, I used conditional logit regression to obtain maximum likelihood estimates, in which I estimated the average utility value $\delta_{jt}^{z}$ for each demographic group in each city every five years. The second step was to decompose the average utility values into labor-related assessments of wages, rents, and amenities. Differentiating the urban average utility estimates of labor in demographic group *z* relative to the base year level yields:

$$\Delta \delta_{jt}^{z}=\left(\Delta w_{jt}^{edu}-\zeta \Delta r_{jt}\right)\beta^{w}z+\Delta a_{jt}\beta^{a}z+\Delta x_{jt}^{A}\beta^{x}z$$ (62)

Define $\Delta \xi_{jt}^{z}$ as the change in unobservable exogenous amenities in the form of utility values in demographic group z of city *j*:

$$\Delta \xi_{jt}^{z}=\beta^{A}z\Delta x_{jt}^{A}$$ (63)

Substituting this into Eq (62), I got:

$$\Delta \delta_{jt}^{z}=\left(\Delta w_{jt}^{edu}-\zeta \Delta r_{jt}\right)\beta^{w}z+\beta^{a}z\Delta a_{jt}+\Delta \xi_{jt}^{z}$$ (64)

### 5.6. Amenity supply

Differentiating the amenity supply relative to its 2005 level yields:

$$\Delta a_{jt}=\gamma^{a}\Delta ln\left(\frac{H_{jt}}{L_{jt}}\right)+\Delta \varepsilon_{jt}^{a}$$ (65)

I estimated all parameters jointly using a two-step GMM method, and standard errors were clustered by city in all estimation equations. All equations contained five-year fixed effects to incorporate national changes over time.

### 5.7. Migration cost

Researchers have shown that migrants generally need to face two challenges in terms of access to urban amenities when choosing a target city to settle in: On the one hand, there is limited access to urban resources brought about by the threshold of household registration (Zhang et al., 2020; Lu, 2016) [74, 75]. On the other hand, due to the limited urban resources previously planned, as population migrates into a city, increasingly more permanent residents compete for the use of urban resources; this causes a decrease in the level of urban per capita resource ownership, which leads to a shortage of urban infrastructure and public services as well as congestion and other “urban diseases” (Lu, 2016) [75]. Therefore, instead of emphasizing the relocation cost directly related to distance or the cost of living directly related to the prices of local goods, the setting of migration costs in this paper focuses on describing the situation where migrants do not have full access to and enjoyment of all urban amenities due to barriers of household registration threshold and the fact that the resident population exceeds the resource carrying capacity of urban infrastructure and public services; therefore, migrants suffer some loss of utility. Following Tombe and Zhu (2019) [76] who constructed the migration costs arising from interprovincial and intersectoral mobility of labor as utility costs, this paper also sets the measured inter-city migration cost as utility costs.

Now, I extend the base model. If migrants choose to live in city *j*, workers can only enjoy some of the urban amenities due to limited access to them. I set this urban amenity distortion with the migration cost *τ*_*z*,*jt*_ as the core variable, that is, as a local tax rate levied on type-z migrants living in city *j*, which will affect the utility of the migrants:

$$1-\frac{1}{1-\tau_{z,jt}}{\left(\delta_{jt}^{Z}Z_{jt}\right)}^{-\tau_{z,jt}}$$ (66)

where *z* ∈ {*H*, *L*} and *τ*_*z*,*jt*_ ≥ 0. The utility component $\delta_{jt}^{z}$ that is available for living in city *j* and is common to all laborers of type-z becomes:

$$\delta_{jt}^{z}{}^{'}=\left(\left(w_{jt}^{edu}-\zeta r_{jt}\right)\beta^{w}z+a_{jt}\beta^{a}z+x_{jt}^{A}\beta^{x}z\right)\frac{1}{\eta_{jt}^{z}}$$ (67)

for high-skilled labor:

$$\eta_{jt}^{H}={\left(\frac{1}{1-\tau_{H,jt}}{\left(\delta_{jt}^{H}H_{jt}\right)}^{-\tau_{H,jt}}\right)}^{-1}$$ (68)

and for low-skilled labor:

$$\eta_{jt}^{L}={\left(\frac{1}{1-\tau_{L,jt}}{\left(\delta_{jt}^{L}L_{jt}\right)}^{-\tau_{L,jt}}\right)}^{-1}$$ (69)

The indirect utility equation that takes migration costs into account is:

$$V_{ijt}=\delta_{jt}^{z}{}^{'}+x_{j}^{prov}prov_{i}\beta^{prov}z_{i}+x_{j}^{region}reg_{i}\beta^{region}z_{i}+\varepsilon_{ijt}$$ (70)

With such a model setting, the higher the migration cost *τ*_*z*, *jt*_, the lower the utility of migrants from urban amenities and vice versa. When *τ*_*z*,*jt*_ = 0, there is no status difference between migrants and the registered population in city *j*, and the planned urban amenities can satisfy the needs of all residents, so access to urban amenities is not restricted, and the local tax rate on migrants’ utility is zero.

## 6. Parameter estimation

### 6.1. Local good expenditure share

In this paper, I used the CMDS to calibrate the local good expenditure share with a target year of 2015 and selected a sample of working household heads aged 15–65 to match the labor in the model. First, I followed the spirit of Davis and Ortalo-Magné (2011) and Wang and Li (2015) [40, 41] and considered housing as the only local good. Then, I followed the spirit of Albouy (2008), Moretti (2013), Lewbel and Pendakur (2009), and Diamond (2016) [1, 23, 43, 77] and considered the additional impact of housing prices on non-housing goods. Thus, I calculated the price of local goods in two cases. One is to treat housing as a local good only, and the other is to treat non-housing goods together with housing as a local good. The only indicator in the CMDS that belongs to the category of non-housing goods and provides an average monthly expenditure is local food. Therefore, in the second case, local goods expenditures consist of both local housing expenditures and local food expenditures.

I also selected the databases used in the calculation of local good expenditure share. The target year for calculating the local good expenditure share in this paper is 2015, and the database should be able to provide data on non-housing commodity expenditures. In addition to the CMDS, other possible options include the Chinese Household Income Project Survey (CHIP), the China Family Panel Studies (CFPS), and the China Household Finance Survey (CHFS). First, the sample sizes of these datasets are much smaller than the size of the CMDS; thus, the number of migrants in each of these datasets is too small for this paper. Second, each of the three datasets had other shortcomings that could not meet the needs of this study. CHIP was excluded because no survey was conducted in 2015, and the adjacent available year is 2013, but using 2013 data as a proxy would cause large errors that would affect the accuracy of the results. The CHFS was excluded because in 2015, it provided expenditure data on consumption expenditure, property expenditure, business expenditure, social security expenditure, and transfer expenditure; however, according to the definitions of each expenditure provided, its statistical scope differs significantly from the needs of this paper. The reason for excluding the CFPS is that no survey was conducted in 2015, and the number of available samples after screening was less than 2000 in 2014 and 2016. In contrast, the CMDS has the following advantages: A survey was conducted in 2015, the sample size was sufficient (about 190000 available samples), and the required data were provided (i.e., food expenditure, housing expenditure, and total expenditure were provided). After a comprehensive comparison, I used the CMDS to calculate the local good expenditure share.

To avoid outliers in the calculation that might be affected by economic fluctuations in a given year, I calculated the local good expenditure share from 2013 to 2015. To assess whether these expenditure shares were due to different average prices faced by laborers with different skills, I further controlled for labor-skill levels as well as the size of the city in which these households were located. City size was divided into five classes according to the Notice on Adjusting the Criteria for Classification of City Size issued by the State Council. Table 4(A) reports the local good expenditure share made up of housing expenditure only: The average housing expenditure for high- and low-skilled workers in 2015 was 24.65% and 18.36%, respectively. Table 4(B) reports the local good expenditure share consisting of food and housing expenditures, in which case there is no significant difference in the local good expenditure share for different labor-skill levels. In combination with the results of the regression coefficients and statistical averages, I set the local good expenditure share at 0.63.

**Table 4:** Local good expenditure share for high- and low-skilled labor from 2013 to 2015.

	2013		2014		2015	
	Statistical mean	Regression coefficients	Statistical mean	Regression coefficients	Statistical mean	Regression coefficients
(A) Housing expenditure						
High skill	0.268	0.234***	0.280	0.250***	0.275	0.247***
Std. Dev.	[0.0041]	[0.0045]	[0.0037]	[0.0043]	[0.0145]	[0.0108]
Low skill	0.207	0.189***	0.199	0.188***	0.194	0.184***
Std. Dev.	[0.0006]	[0.0019]	[0.0007]	[0.0021]	[0.0081]	[0.0094]
(B) Housing and food expenditure						
High skill	0.678	0.661***	0.684	0.663***	0.649	0.620***
Std. Dev.	[0.0043]	[0.0049]	[0.0039]	[0.0046]	[0.0090]	[0.0107]
Low skill	0.686	0.675***	0.672	0.661***	0.646	0.628***
Std. Dev.	[0.0008]	[0.0024]	[0.0009]	[0.0025]	[0.0054]	[0.0104]

Note: The industry classification excludes agriculture, and the sample was the employed workers aged 15 to 65 across cities. Standard errors are in square brackets.

*** Significant at the 1% level.

** Significant at the 5% level.

* Significant at the 10% level.

### 6.2. Migration costs

I used the product of two parameters *λ* and *μ*_*z*,*jt*_ to represent the migration cost *τ*_*z*,*jt*_: *τ*_*z*,*jt*_ = *λ* · *μ*_*z*,*jt*_, where *μ*_*z*,*jt*_ denotes the migration costs across cities calculated in this paper with reference to methods in previous literature, and *λ* denotes the coefficient that adjusts the calculation results according to the lower bound of utility that migrants can afford to make urban location choices.

In this paper, I used the urban household registration threshold index multiplied by the migrant-ratio index to measure the migration cost *μ*_*z*,*jt*_ incurred by migrants due to a lack of local household registration and limited access to urban resources when they live in a different city. I used the household registration threshold index constructed by Zhang and Lu (2019) [78] to measure migrants’ limited access to urban resources due to the household registration threshold. The original index included only 120 cities, so I calculated the average household registration threshold for each type of city according to city size and filled in the cities with missing data.

I used the migrant-ratio index constructed by Han and Lu (2018) [79] to measure the gap between the planned and actual number of users of urban infrastructure, social security, public services, and other resources: migrant ratio = (resident population—registered population) / registered population. To eliminate the interference of a negative migrant ratio in the calculation of migration costs, the calculation results of the migrant ratio were normalized.

Previous literature has reached slightly different conclusions on how much productivity gains can be achieved by eliminating labor market distortions, but they are around 20%. Pan et al. (2018) [80] calculated the result as 19.78%, Gai et al. (2019) [81] calculated the result as 20.51%, Huang and Wang (2021) [82] calculated the result as 18.05%, and Zhang et al. (2021) [83] calculated the result as 21.6%. In this paper, I set this value to 20%, which means that eliminating labor market distortions can increase productivity by 20%. According to Tombe and Zhu (2019) [76], when land is not used as an input factor in the production process, the proportional relationship between the labor productivity improvement and the welfare improvement driven by the reduction in domestic migration costs is 1:1.58; that is, eliminating labor market distortions, a 20% increase in productivity can increase the level of labor utility by up to 31.6%. That is to say, labor market distortions reduce not only the level of productivity but also the level of labor utility, which is only 75.76% of what it would be in the ideal case without distortions. Based on this finding, I set the lower bound on the utility that migrants can accept in their city of residence to 75% of the utility level in the undistorted case. If the migration cost *τ*_*z*,*jt*_ is too high, the local tax rate on the utility received by a migrant living in city *j* will be significantly higher. Once the level of utility available to the migrant is below the lower bound of utility, he or she will make a new choice: whether to apply for local household registration and become a registered population or choose to live in another city. According to the above setting, the value of *λ* was set to 0.012 by combining the migration cost *μ*_*z*,*jt*_ across cities.

### 6.3. Labor supply

This paper presents parameter estimates for four specific forms of the model to highlight the importance of endogenous amenities and productivity in influencing migration, wages, and rents from 2005 to 2015.

I refer to Model (1) as the “standard model”, assuming that local amenities and firms’ local productivity levels are exogenous and that the elasticity of local demand is determined only by the labor substitution elasticity *ρ* between high and low skills, that is, estimate labor demand Eqs (10) and (11), where *f*_*H*_ (*H*_*jt*_, *L*_*jt*_) = 0, *f*_*L*_ (*H*_*jt*_, *L*_*jt*_) = 0. Households’ local good expenditure share is not calibrated to highlight how labor trades off between wages and local prices when amenities are assumed to be exogenous. The estimation results of Model (1) are shown in Column 1 of Table 5. The results show that in Model (1), high-skilled workers prefer high wages and low rents, while low-skilled workers have a positive demand elasticity for rents, so under the same conditions, the real wages of low-skilled workers are lower than those of high-skilled workers. High- and low-skilled workers do not have the same trade-off between wages and rents, which implies a difference in their local good expenditure share. If only housing expenditures are considered when calculating the local good expenditure share, then according to the setup of Model (1), high- and low-skilled workers are willing to spend about 12.6% and about 27.8% of their expenditures on local goods, respectively. The estimation results suggest that the difference in skill mix across cities is because the local good expenditure share of low-skilled labor is more than twice as large as the share of high-skilled labor. However, the parameter estimate of the 12.6% expenditure share is rejected by the CMDS. Table 4 shows that when only housing expenditures are considered, the local good expenditure share of high-skilled workers, which is also the lower bound of all local goods consumption, is about 24.7%; the large gap between the high-skill local good expenditure share and low-skill local good expenditure share estimated by Model (1) is also rejected by the CMDS. The main difference between the two calculations is that the high-skill local good expenditure share estimated by the CMDS is higher than the share of low-skilled labor, while the high-skill local good expenditure share estimated by Model (1) is lower than the share of low-skilled labor.

**Table 5:** GMM estimates of model parameters.

	Low skill	High skill	Low skill	High skill	Low skill	High skill	Low skill	High skill
	Model (1)		Model (2)		Model (3)		Model (4)	
(A) Labor’s preference for cities								
Wage	13.545**	10.404***	1.617***	5.011***	0.306	1.891	3.680	4.018
	[6.809]	[3.799]	[0.364]	[1.510]	[0.349]	[1.236]	[2.374]	[2.698]
Rent	3.759	-1.307	-1.019***	-3.157***	-0.193	-1.192	0.635	-0.754
	[2.833]	[1.248]	[0.229]	[0.951]	[0.220]	[0.779]	[0.756]	[0.659]
Local good share	0.278	0.126	0.630	0.630	0.630	0.630	0.173	0.188
Amenity	-	-	-	-	0.064***	0.092**	0.041	0.090**
					[0.015]	[0.038]	[0.029]	[0.046]
Differential effects: Interprovincial migration								
Wage	-1.932*	-0.267	-0.551***	-0.378	-0.482***	-4.862**	-2.524*	-16.778**
	[1.045]	[2.684]	[0.158]	[1.016]	[0.165]	[2.437]	[1.533]	[7.507]
Rent	-0.236	0.817	0.347***	0.238	0.303***	3.063**	-0.562	-0.131
	[0.427]	[0.905]	[0.100]	[0.640]	[0.104]	[1.536]	[0.576]	[2.129]
Amenity	-	-	-	-	-0.005	0.127**	0.007	0.219**
					[0.007]	[0.057]	[0.020]	[0.107]

Model (2) adjusts the local expenditure share to 0.63 based on Model (1) and estimates only the elasticity of labor migration with respect to wages. I call it the “restricted standard model.” The estimation results are presented in Column 2 of Table 5. The estimation results show that the wage elasticity of high-skilled labor decreases to 48.2% of the wage elasticity in Model (1), while the rent elasticity increases to 2.42 times that in Model (1). That is, when the high-skill local good expenditure share is calibrated from 25% to 63%, the utility gain from higher wages will be nearly halved, while the utility reduction from higher rents will be 1.4 times greater. Large cities can provide higher wages, but they also have higher living costs. Obviously, for high-skilled labor, the utility reduction from choosing a large city with a higher cost of living is greater than the utility increase from higher wages. This echoes the inference of the stylized facts that there is a factor other than wages driving the strengthening of the spatial sorting trend. This factor should be positively correlated with local prices affected by the Bartik shock and housing supply. Changes in amenities could explain this puzzle. I tested the over-identification constraint, and the test results for Model (1) and Model (2) (p-values of 0.3233 and 0.2059, respectively) accepted the null hypothesis of the over-identification test that all instrumental variables are exogenous, there is no over-identification problem. This further supports my inclusion of the endogenous amenity variable in the model.

The third column of Table 5 presents the estimation results of Model (3). The local good expenditure share in Model (3) remained at 0.63, adding urban endogenous amenities and relaxing the constant substitution elasticity function form of land demand, allowing for a more flexible model of labor demand. I refer to this as the “full model.” According to the estimates, both high- and low-skilled workers prefer higher wages, lower rents, and higher levels of amenities. However, there is also heterogeneity in preferences between high- and low-skilled workers, with the key difference being how they value wages, amenities, and the relative value of real wages versus the level of amenities. The migration elasticity with respect to wages for high skills (1.891) is larger than that for low skills (0.306), indicating that high skills are more sensitive to changes in wages. Similarly, high-skilled workers are also more sensitive to the level of amenities (0.092 > 0.064), possibly because high-skilled workers are more capable of breaking through migration barriers and choosing a new city to settle in than low-skilled workers, and therefore they are also more attentive to the key characteristics of the new city. Model (3) also passes the test of the over-identification constraint, and the instrumental variables do not have an over-identification problem. The endogenous amenity index added to the model captures previously overlooked variables.

The fourth column of Table 5 presents the estimation results of Model (4), which removes the assumption of 0.63 for the local good expenditure share and attempts to identify this parameter from the census data. The estimation results of Model (4) are noisier due to the correlation between housing rent and amenities, but the main conclusion that a high-skilled worker prefers higher wages, lower rents, and higher levels of amenities still holds. Since only monthly housing expenditures are available in the census data, using it as the local good expenditure yields a share of about 18.8% for high-skilled labor and 17.3% for low-skilled labor. This result is essentially the same as the estimate of 23% in the literature (Wang and Li, 2015) [41] and in my calculations based on CMDS data.

The bottom half of Table 5 reports the heterogeneity of preferences for labor migration across provinces. Overall, compared to the base regression results, interprovincial migrants face lower real wages, while high-skill interprovincial migrants have higher amenity elasticity than intra-provincial migrants; that is, high-skill interprovincial migrants are more concerned with the level of amenities in their city of residence.

### 6.4. Housing supply

Table 6(B) presents the estimates of inverse housing supply elasticity. The overall level of my estimates was determined by the base inverse housing supply elasticity term *γ*. The mean value of the inverse elasticity of the base housing supply is 0.548, with a standard deviation of 0.019. The estimates of the inverse elasticity of the base housing supply do not differ significantly across the four model specifications, which is not surprising since they all share the same housing supply model. Consistent with the work of Fan et al. (2015) [84] and Lu et al. (2015) [85], the results in Table 6(B) show that rent increases are higher in cities with higher land regulation and higher in cities with higher land unavailability. In other words, housing supply elasticity is lower in areas with higher levels of land use regulation and in areas with a lower share of land available for real estate development.

**Table 6:** GMM estimates of model parameters (continued).

	Model (1)	Model (2)	Model (3)	Model (4)
(B) Housing Supply				
EXP (land use regulation)	0.002	0.009	0.009	0.012
	[0.059]	[0.019]	[0.016]	[0.050]
EXP (Land unavailability)	0.037	0.032*	0.024*	0.028
	[0.056]	[0.019]	[0.017]	[0.042]
Base housing supply elasticity	0.534	0.576**	0.537**	0.545
	[0.784]	[0.303]	[0.265]	[0.571]
(C) Labor demand				
ρ	0.947***	0.914***		
	[0.044]	[0.047]		
The elasticity of high-skill wage w.r.t high-skill emp.			-0.066	-0.022
			[0.060]	[0.054]
High-skill wage w.r.t low-skill emp.			0.067	0.052
			[0.080]	[0.064]
Low-skill wage w.r.t low-skill emp.			-0.187**	-0.160*
			[0.088]	[0.092]
Low-skill wage w.r.t high-skill emp.			0.212***	0.201***
			[0.073]	[0.075]
(D) Amenity supply				
High-skill emp. ratio			1.080	0.589
			[1.521]	[1.818]
Hansen’s J (p-value)	0.323	0.206	0.285	0.712
Endogenous amenities Index			-	-
Calibrated local good expenditure share		-	-	
CES labor demand	-	-		
Reduced-form labor demand			-	-

Note: Standard errors are in square brackets. The data include 861 samples from 287 cities over three years. The base year is 2005. Standard errors are clustered by city.

*** Significant at the 1% level.

** Significant at the 5% level.

* Significant at the 10% level.

### 6.5. Labor demand

The parameter estimates of the local labor demand curve are presented in Table 6(C). The estimated ρ for Model (1) is 0.947, which implies that the labor elasticity of substitution is 18.87. The estimated ρ for Model (2) is 0.914. The parameter estimates are consistent with those of Zhao and Yuan (2017) [86]. A higher elasticity of labor substitution indicates that there is an imperfect substitution relationship between high- and low-skilled labor (Card, 2009) [28], and that technological progress favors high-skilled labor.

Models (3) and (4) estimate more flexible labor demand curves [56] and [57]. Based on the results in Column 3 of Table 6(C), I can first reject the assumption that the elasticity of high-skilled labor demand with respect to high-skill wages is equal to the elasticity of low-skilled labor demand with respect to low-skill wages. In the standard CES production function commonly used in the literature, these two elasticities are often assumed to be the same. Second, the sign of the elasticity of high-skill wages with respect to high-skill employment is negative but insignificant, suggesting a powerful knowledge spillover between high skills in addition to the competitive relationship. This force contributes to the productivity of all workers, thus increasing their wages in areas with high concentrations of high skills. The sign of the elasticity of low-skill wages with respect to low-skill employment is negative and significant at the 5% level, indicating that there is no strong knowledge spillover between low skills but rather a competitive relationship between them. The elasticity of high-skill wages with respect to low-skill employment is positive but insignificant, and the elasticity of low-skill wages with respect to high-skill employment is positive and significant at the 1% level, indicating that there is skill complementarity between high- and low-skilled labor and that the gains from skill complementarity are more significant for low-skilled labor. This is intuitive because high skills may be subject to low-skill shocks, resulting in a slight decline in high-skill productivity; this would affect high-skill earnings. In general, competitive relationships dominate same-skilled labor, while complementary relationships dominate high- and low-skilled labor. The elasticity estimates in Column 4 also support these findings.

### 6.6. Amenity supply

The elasticity of the amenity supply with respect to the high-skill employment ratio is presented in Table 6(D). A positive elasticity implies that an increase in the ratio of high-skill employment endogenously improves local amenities in cities. However, the relationship is not significant, indicating that growth in the level of amenities in large cities has not been able to match the spatial sorting trend of migrants in China, which suggests that there are differences in the endogenous mechanism of amenity supply between Chinese and American cities. Since high-skilled workers earn higher wages than low-skilled workers, they have greater abilities to choose locations with high levels of amenities, and their strong demand for amenities will also lead to an increase in the level of amenities in areas with a high concentration of high-skilled workers. This mechanism has been validated in a large amount of literature using U.S. data (Bayer et al., 2007; Guerrieri et al., 2013; Handbury, 2021) [15, 18, 19]. However, in China, this story is slightly different. First, one of the objectives of the Chinese government’s poverty alleviation policies and transfer payment policies is to ensure basic living conditions of residents in less-developed areas and small cities; the implementation of such policies and measures has significantly improved the level of amenities in less-developed areas and small cities. Second, the construction of some amenities, such as urban infrastructure, is based on historical projections of population growth, and the low mobility of population in the early years made historical projections greatly underestimate the actual population growth in large cities. The supply of some amenities in large cities was not designed with a sufficient margin for an increasing population in the future. Consequently, the development of large cities is often accompanied by the problem of “urban diseases.” Third, some amenities, such as social insurance and public services, rely mainly on local financial support, and limited fiscal revenues will also reduce the growth rate of the amenity level in large cities. Finally, China’s household registration policy restricts migration, and the trend of migrants’ spatial sorting is suppressed, which directly weakens the increase in amenity supply led by high-skilled workers’ demand in large cities. This also results in many high-skilled workers failing to obtain household registration in large cities, so they will save a portion of their income earned in large cities to pay for their future living expenses in small cities, which indirectly reduces the demand for amenities among high-skilled workers in large cities. For these reasons, an increase in the amenity supply in China is not fully dominated by high-skilled workers’ demand for amenities, and this resulted in a positive but insignificant regression coefficient.

## 7. Urban amenity and productivity

The exogenous productivity of local firms and the attractiveness of local amenities in each city can be inferred using the estimated results of the model parameters. Much of the literature has used hedonic techniques to estimate which cities provide the most desirable amenities. In this paper, I used a different method to infer the level of amenities in each city. Recalling Eq (64), the utility value of a city’s amenities to the labor of a given demographic group is measured as a component of the common utility level of labor in each city, which is not controlled by local wages and rents. Therefore, $Amen_{jt}^{z}$, the utility from amenities of type-z labor in city *j* in year *t* can be written as follows:

$$Amen_{jt}^{z}=\left(a_{jt}\beta^{a}z+\xi_{jt}^{z}\right)\frac{1}{\eta_{jt}^{z}}=\delta_{jt}^{z}{}^{'}-\frac{1}{\eta_{jt}^{z}}\left(w_{jt}^{edu}-\zeta r_{jt}\right)\beta^{w}z$$ (71)

where $\eta_{jt}^{z}={\left(\frac{1}{1-\tau_{z,jt}}{\left(\delta_{jt}^{Z}Z_{jt}\right)}^{-\tau_{z,jt}}\right)}^{-1}$. Given the wage and rent levels in cities and the labor’s preferences for wages and rents, it can be intuitively inferred that cities with higher-than-expected population levels for specific demographic groups have higher levels of amenities. Similarly, it is possible to analyze which cities have the highest and lowest productivity levels.

Through the regression of the model-predicted change in urban high-skill productivity and the model-predicted change in low-skill productivity, I found that the regression coefficient of 0.025 for the per capita level of local high-skill productivity change and local low-skill productivity change was not significant, and the *R*^2^ was low, indicating that there is only a weak positive relationship between the two, that is, there is a huge difference between local high-skill productivity and low-skill productivity changes. Table 7 shows that the regression coefficient of 0.604 for the change in high-skill wages and the change in low-skill wages was significant at the 1% level, indicating a strong positive correlation between the two with an *R*^2^ of 0.343, which means that the change in low-skill utility due to the change in wages explains about 34% of the change in high-skill utility due to the change in wages in the same city.

**Table 7:** The relationship between amenity and productivity changes.

	(1)	(2)	(3)	(4)
	Δ Endogenous high-skill amenity	Δ Exogenous high-skill amenity	Δ High-skill wage	Δ High-skill productivity
Δ Endogenous low-skill amenity	1.698***			
	[0.1499]			
Δ Exogenous low-skill amenity		0.210***		
		[0.0185]		
Δ Low-skill wage			0.604***	
			[0.0498]	
Δ Low-skill productivity				0.0252
				[0.0423]
Constant	1.670***	-1.057***	0.321***	1.038***
	[0.2204]	[0.0517]	[0.0537]	[0.0327]
Observations	234	234	284	284
*R* ^2^	0.353	0.356	0.343	0.001

Note: Standard errors are in square brackets. Productivity was measured at the per capita level. Amenity changes and productivity changes were measured from 2005 to 2015. Urban amenity and productivity levels were inferred from the model estimates.

*** Significant at the 1% level.

** Significant at the 5% level.

* Significant at the 10% level.

Note that simply comparing the relationship between changes in local high-skill wages and changes in local low-skill wages is unlikely to reveal a weak positive relationship between local high-skill productivity changes and low-skill productivity changes, with the movement along the local labor demand curve driven by migration masking the large differences in local productivity changes between different skills.

The preferences of high- and low-skilled workers for urban amenities are relatively close. In general, the overall utility valuation of urban amenities by high-skilled labor is positively correlated with the utility valuation of the same urban amenities by low-skilled labor. According to the results presented in Table 7, the change in the utility value of high-skill amenities and the change in the utility value of low-skill amenities across cities are strongly positively correlated, regardless of whether the amenities are endogenous or exogenous. The difference mainly lies in the magnitude of the coefficients. For every 1% increase in the utility of endogenous amenities for low-skilled labor, the utility of endogenous amenities for high-skilled labor increased by 1.698%; for every 1% increase in the utility of exogenous amenities for low-skilled labor, the utility of exogenous amenities for high-skilled labor increased by 0.210%. This implies that high-skilled labor will gain more utility from changes in endogenous amenities than low-skilled labor, while high-skilled labor will gain less utility from changes in exogenous amenities than low-skilled labor. This result confirms, from a utility perspective, that endogenous amenities are an important force driving the spatial sorting of high-skilled labor.

The *R*^2^ results show that the change in low-skill utility due to endogenous amenity changes in the cities explains 35.3% of the change in high-skill utility for the same cities’ amenities; the change in low-skill utility due to exogenous amenity changes explains 35.6% of the change in high-skill utility for the same cities’ amenities.

Migration costs weaken labor mobility and reduce the spatial sorting trend of migrants. If migration costs increase, migration will be more difficult and the number of migrants will decrease, but the problem of “urban diseases” in large cities due to excessive resident populations will be alleviated to some extent. If migration costs decrease, according to Eqs (66) and (67), a decrease in *τ*_*z*,*jt*_ leads to a decrease in $\eta_{jt}^{z}$ and an increase in $\delta_{jt}^{z}{}^{'}$. After substituting specific values into Eq (71), it was found that a decrease in *τ*_*z*,*jt*_ leads to an increase in $Amen_{jt}^{z}$, and simultaneously, the number of migrants will increase; that is to say, more high- and low-skilled workers will be able to obtain household registration in large cities and, thus, have higher levels of amenities. However, if the population exceeds a city’s carrying capacity, the resident population will compete more fiercely for urban resources. Like high-skilled workers, low-skilled workers also prefer higher levels of amenities, but cities with higher levels of amenities tend to have higher migration costs, and high-skilled workers have a greater ability to break through migration barriers than low-skilled workers, so migration costs have a relatively greater impact on the migration of low-skilled workers. A fraction of workers who forgo being registered in a large city due to high migration costs tend to make a new location choice and choose to live in a small city, while others tend to choose to work in a large city when they are young and relocate to a small city in the future.

## 8. Impact of registered population on location choice

Distribution of the registered population affects workers’ location choice from three perspectives. First, the proportion of migrants to the resident population is relatively small in the majority of cities in China, and the registered population is the main component of the resident population. The larger the registered population, the larger the city size tends to be. According to Eqs (66) and (67), the larger the *Z*_*jt*_, the larger the $\eta_{jt}^{z}$, and the smaller the $\delta_{jt}^{z}{}^{'}$, the fewer indirect utilities migrants can have, which means that the registered population affects migrants’ indirect utilities through the city size channel and, thus, has an impact on workers’ location choices. Second, the distribution of the registered population has an impact on mobility costs: According to the migrant-ratio formula in Section 6.2, the higher the proportion of the registered population to the resident population, the lower the proportion of migrants to the resident population in the city, and the lower the migrant ratio, the lower the migration costs *μ*_*z*,*jt*_. This means that the registered population influences workers’ location choices via the channel of competition intensity among migrants. Third, the household registration threshold index Zhang and Lu (2019) [78] used in this paper was quantified by city size hierarchy. Since the registered population is the main component of the resident population in the majority of cities in China, generally speaking, the larger the registered population, the larger the city size tends to be, and the larger the value of the household threshold index, the higher the difficulty of moving in and settling down. That is, the registered population has an impact on workers’ location choices via the urban settlement threshold channel.

## 9. Determinants of urban high-skill employment ratio changes

I used reduced-form regression between exogenous productivity changes estimated from the model and high-skill employment ratios to assess the role of local productivity changes in driving local migration patterns. The regression equation is as follows:

$$ln\left(\frac{H_{j2015}}{L_{j2015}}\right)-ln\left(\frac{H_{j2005}}{L_{j2005}}\right)=\beta_{1}\left(\varepsilon_{j2015}^{H}-\varepsilon_{j2005}^{H}\right)+\beta_{2}\left(\varepsilon_{j2015}^{L}-\varepsilon_{j2005}^{L}\right)+\epsilon_{j}$$ (72)

According to the figures in Column 1 of Table 8, changes in high-skill exogenous productivity strongly predict increases in high-skill employment ratios, while changes in low-skill exogenous productivity strongly predict decreases in high-skill employment ratios. Furthermore, the *R*^2^ of this regression suggests that 42% of the changes in the urban high-skill employment ratio can be explained by changes in local productivity.

**Table 8:** Reduced-form relationship between high-skill employment ratio, local real wages, and local employment shocks.

	(1)	(2)	(3)	(4)	(5)	(6)
	Δ High-skill employment ratio	Δ High-skill employment ratio	Δ High-skill employment ratio	Δ High-skill employment ratio	Δ High-skill local real wage	Δ Low-skill local real wage
Δ High-skill local real wage				0.0239		
				[0.284]		
Δ Low-skill local real wage				0.303		
				[0.311]		
Δ High-skill productivity	1.723***		1.648***		0.794***	0.470***
	[0.188]		[0.189]		[0.0828]	[0.0795]
Δ Low-skill productivity	-2.032***		-1.895***		-0.137*	0.234***
	[0.172]		[0.183]		[0.0748]	[0.0718]
Δ High-skill amenity		-0.158***	-0.0816*			
		[0.0600]	[0.0483]			
Δ Low-skill amenity		-0.0392*	-0.00782			
		[0.0207]	[0.0170]			
Constant	0.957***	1.066***	0.826***	1.140***	-0.128	-0.0127
	[0.177]	[0.0759]	[0.184]	[0.104]	[0.0811]	[0.0779]
Observations	234	234	234	234	282	282
*R* ^2^	0.423	0.102	0.437	0.017	0.249	0.179

Note: Standard errors are in square brackets. The period of change was from 2005 to 2015, weighted by the 2005 urban population. The high-skill employment ratio was defined as the number of workers between the ages of 15 and 65, with a bachelor’s degree or higher, and who were employed living in a city, divided by the number of workers between the ages of 15 and 65 with a high school degree or less who were employed. Δ Real wage = Δ ln(wage)-0.63*Δ ln(rent).

*** Significant at the 1% level.

** Significant at the 5% level.

* Significant at the 10% level.

I evaluated the predictive effect of model-inferred exogenous amenity changes $\Delta \xi_{jt}^{z}$ on high-skill employment ratio changes for comparison. According to the figures in Column 2 of Table 8, exogenous amenity changes negatively predict high-skill employment ratio changes, but the explanatory power is not very high, with an *R*^2^ of only 0.102. I combined exogenous amenity changes and exogenous productivity changes in the same regression, the results are presented in Column 3 of Table 8. Similarly, exogenous productivity changes strongly predict high-skill employment ratio changes. Compared to the regression using only productivity changes, the *R*^2^ of the regression including exogenous amenity changes increased by only 0.014. Thus, it can be seen that local productivity changes are the main driver of urban high-skill employment ratio changes.

The next question is whether endogenous amenity changes are a key channel through which local productivity changes lead to changes in high-skill employment ratio. The relationship between local real wage changes and the high-skill employment ratio should be examined first. Since changes in local productivity exert a significant influence on changes in the high-skill employment ratio, changes in local real wages should be a main independent variable explaining changes in the high-skill employment ratio. Local real wages are defined as the wages net of local good prices:

$$rw_{jt}^{edu}=w_{jt}^{edu}-0.63\times r_{jt}$$ (73)

The figures in Column 4 of Table 8 shows a weak positive correlation between the increases in high-skill real wages and the changes in high-skill employment ratio. The results show that real wages can still explain the changes in high-skill employment ratio to some extent, but real wages are not the main driver of the increase in the spatial sorting trend. Those high-skilled workers who increasingly choose to live in cities with lower real wages even have to compensate for the lower real wages through urban amenities. Therefore, these reduced-form regression results are remarkably consistent with the stylized facts as well as the structural model estimates discussed earlier.

The results of estimating the impact of local productivity changes on real wages are presented in Column 5 of Table 8, and it is easy to see that an increase in high-skill productivity leads to an increase in high-skill real wages. High-skilled labor, who are paid high wages due to their high productivity, migrates to target cities and housing prices are thus pushed up. If such migration is accompanied by an increase in urban amenity levels, the migration trend stops when higher rent prices offset the benefits of high wages and high levels of urban amenities. The still-increasing spatial sorting trend implies that for high-skilled labor, the current increase in local good prices has not fully offset the benefits of higher incomes from migration and increased amenities.

The results of a similar regression for low-skill real wages are presented in Column 6 of Table 8. An increase in low-skill productivity leads to an increase in low-skill real wages, while an increase in high-skill productivity also leads to an increase in low-skill real wages. Column 5 of Table 8 shows that an increase in low-skill productivity leads to a decrease in real wages for high-skilled workers. This suggests that complementarity between high and low skills has a positive effect on the increase in low-skill real wages. Although this has some negative effects on high-skilled labor, with a slight decrease in the high-skill real wages, the absolute value of the elasticity of low-skill real wages with respect to the high-skill productivity changes is larger than the absolute value of the elasticity of high-skill real wages with respect to the low-skill productivity changes. These results imply that the net effect of skill complementarity is positive, and that skill complementarity is more beneficial to low-skilled labor.

Through the structural equation regression in Table 6(B) and the reduced-form regression in Table 8, this paper provides a detailed description of the relationship between skill complementarity and knowledge spillover among workers with different skills. Summarizing the results, this paper finds that there is strong competition within same-skilled labor and that there is a strong knowledge spillover between high- and low-skilled labor.

The agglomeration of high-skilled labor raises the productivity of high-skilled labor as well as low-skilled labor, which is reflected in incomes—that is, higher wages for high- and low-skilled labor. Competitive relationships dominate within low-skilled labor, and there are few knowledge spillovers within them. The main spillover they receive comes from high-skilled labor; the net benefit of skill complementarity between high- and low-skilled labor is positive, but skill complementarity with low-skilled labor will negatively affect high-skill productivity to some extent, which will eventually be reflected in lower wages for high-skilled labor. Complementarity between high- and low-skilled labor has a greater positive impact on low-skilled labor, significantly raising the wages of low-skilled labor.

## 10. Welfare implications and welfare inequality

From 2005 to 2017, the nominal wage gap and the real wage gap between high- and low-skilled labor gradually narrowed. However, changes in wage inequality do not necessarily coincide with changes in welfare inequality in the same direction. The additional welfare effects of local rents and amenities may increase or offset the welfare effects of wage changes. To measure how changes in urban wages, rents, and amenities affect welfare inequality, this paper performs a welfare decomposition. First, I hold local rents and amenities constant, assume that only urban wages change, and calculate the expected utility change for each labor force from 2005 to 2015. The expected utility of labor *i* from the city where he or she prefers to live can be written as follows:

$$E\left(U_{i2005}\right)=ln(\underset{j}{\sum }exp((\beta^{w}z_{i}w_{j2005}^{edu}-\beta^{r}z_{i}r_{j2005}+\xi_{j2005}^{z}+\beta^{a}z_{i}(\gamma^{a}ln\left(\frac{H_{j2005}}{L_{j2005}}\right)+\varepsilon_{j2005}^{a}))\frac{1}{\eta_{j2005}^{z}}+\beta^{prov}z_{i}prov_{i}x_{j}^{prov}+\beta^{region}z_{i}region_{i}x_{j}^{region}))$$ (74)

If wages are adjusted to the level actually observed in 2015, then the expected utility of labor *i*, $E\left({\hat{U}}_{i2015}^{w}\right)$, can be written as follows:

$$E\left({\hat{U}}_{i2015}^{w}\right)=ln(\underset{j}{\sum }exp((\beta^{w}z_{i}w_{\text{j}2015}^{\text{edu}}-\beta^{r}z_{i}r_{\text{j}2005}+\xi_{\text{j}2005}^{z}+\beta^{a}z_{i}(\gamma^{a}\text{ln}\left(\frac{H_{\text{j}2005}}{L_{\text{j}2005}}\right)+\varepsilon_{j2005}^{a}))\frac{1}{\eta_{j2005}^{z}}+\beta^{prov}z_{i}prov_{i}x_{j}^{prov}+\beta^{region}z_{i}region_{i}x_{j}^{region}))$$ (75)

If wages and rents are adjusted to the levels actually observed in 2015, the expected utility of labor *i* can be expressed as follows:

$$E\left({\hat{U}}_{i2015}^{wr}\right)=ln(\underset{j}{\sum }exp((\beta^{w}z_{i}w_{j2015}^{edu}-\beta^{r}z_{i}r_{j2015}+\xi_{j2005}^{z}+\beta^{a}z_{i}(\gamma^{a}ln\left(\frac{H_{j2005}}{L_{j2005}}\right)+\varepsilon_{j2005}^{a}))\frac{1}{\eta_{j2005}^{z}}+\beta^{prov}z_{i}prov_{i}x_{j}^{prov}+\beta^{region}z_{i}region_{i}x_{j}^{region}))$$ (76)

If wages, rents, and endogenous amenities due to resorting of workers are adjusted to the levels actually observed in 2015, the expected utility of labor *i* can be expressed as follows:

$$E\left({\hat{U}}_{i2015}^{wr}\right)=ln(\underset{j}{\sum }exp((\beta^{w}z_{i}w_{j2015}^{edu}-\beta^{r}z_{i}r_{j2015}+\xi_{j2005}^{z}+\beta^{a}z_{i}(\gamma^{a}ln\left(\frac{{\hat{H}}_{j2015}}{{\hat{L}}_{j2015}}\right)+\varepsilon_{j2005}^{a}))\frac{1}{\eta_{j2005}^{z}}+\beta^{prov}z_{i}prov_{i}x_{j}^{prov}+\beta^{region}z_{i}region_{i}x_{j}^{region}))$$ (77)

where ${\hat{H}}_{j2015}=\frac{H_{j2015}}{H_{2015}}H_{2005},\;{\hat{L}}_{j2015}=\frac{L_{j2015}}{L_{2015}}L_{2005}$.

The change in expected utility measures the willingness to pay of each labor force living in his preferred counterfactual city in terms of logarithmic wages. I calculated the expected utility change for each labor force driven only by wage changes from 2005 to 2015, and compared the average utility impact on high-skilled labor with the average utility impact on low-skilled labor.

Column 1 of Table 9 shows that from 2005 to 2015, the increase in the welfare gap between high- and low-skilled labor due to wage changes was equivalent to an increase of 0.039 log points in the wage gap between high- and low-skilled labor in the country, which was contrary to the ten-year trend of a 0.141 log-point decrease in the wage gap between high- and low-skilled labor. Even if local amenities and rents do not change, the welfare inequality between high- and low-skilled labor still increases due to local wage changes. Column 2 takes into account the additional effect of changes in local rents, showing that the change in welfare inequality between high- and low-skilled labor due to changes in wages and rents over ten years was equivalent to a 0.095 log-point increase in the wage gap between high- and low-skilled labor. The effect of wages and rents on welfare results in a large increase in welfare inequality. This is because while the rent gap between high- and low-skilled labor widens, and rents are higher in cities that offer desirable wages for high-skilled labor, the ratio of rents to wages for high-skilled labor is lower than that for low-skilled labor, so it is less stressful for high-skilled labor to pay the rents.

**Table 9:** Welfare inequality decomposition from 2005 to 2015: wages, rents, and amenities.

Year	(1)	(2)	(3)
2005	0.611	0.611	0.611
	-	-	-
2010	0.714	0.761	0.762
	[0.0051]	[0.0133]	[0.0705]
2015	0.650	0.706	0.698
	[0.0131]	[0.0225]	[0.0835]
2005–2015 change	0.039	0.095	0.087
Wages	-	-	-
Rents		-	-
Endogenous amenities from resorting of workers			-

Note: The welfare gap was converted to the 2005 wage gap between high- and low-skilled labor.

Column 3 adds to the changes in endogenous amenities caused by changes in the high-skilled employment ratio based on changes in wages and rents. I measured the impact of amenity changes on welfare inequality driven solely by labor resorting, fixing the national high-skilled labor share at 2005 levels. The ten-year change in welfare inequality between high- and low-skilled labor caused by changes in wages, rents, and endogenous amenities driven by labor resorting was equivalent to a 0.087 log-point increase in the wage gap between high- and low-skilled labor. Welfare inequality increased by 123% compared to the case in which only the impact of wages on welfare was considered, and welfare inequality decreased by 8.4% compared to the case in which the impacts of wages and rents on welfare were considered. The results suggest that improvements in endogenous amenities contribute to reducing the welfare inequality gap between high- and low-skilled labor.

Assuming that the migration costs that distort the labor market are eliminated, migrants have full access to all resources (including urban amenities in their resident cities), and the utility loss of the labor leaving their places of residence to move across cities is reduced to zero. Under such an assumption, I once again measured how changes in urban wages, rents, and amenities from 2005 to 2015 impacted social welfare inequality.

Table 10 shows that when migration costs are removed, the expected utility changes driven by wages and the expected utility changes driven by wages and rents over ten years were basically the same as when migration costs were present; however, the changes in welfare inequality caused by changes in wages, rents, and endogenous amenities have changed significantly. When migration costs were eliminated, the change in welfare inequality between high- and low-skilled labor caused by changes in wages, rents, and endogenous amenities driven by labor resorting over a decade was equivalent to a reduction of 0.145 log points in the wage gap between high- and low-skilled labor. The magnitude of this change is 25% larger than the ten-year reduction in the real wage gap between high- and low-skilled labor observed in the data, and 3% larger than the nominal wage gap in the ten-year reduction in the data. Welfare inequality decreased by 453% compared to the case where only the impact of wages on welfare was taken into account and migration costs were eliminated. Welfare inequality decreased by 251% compared to the case where the impact of wages and rents on welfare was considered and migration costs were eliminated. Welfare inequality decreased by 267% compared to the case where the impact of wages, rents, and endogenous amenities on welfare was considered, and migration costs were taken into account. The counterfactual results suggest that, on the one hand, an increase in the level of amenities facilitates the reduction of the welfare inequality gap between high- and low-skilled labor. On the other hand, if migration costs across cities are eliminated, migrants’ access to urban amenities is no longer restricted, low-skilled labor can enjoy the more desirable amenities and gain additional utility compared to high-skilled labor. The welfare increases more for low-skilled labor, the effect of urban amenities in reducing the welfare inequality gap between high- and low-skilled labor will be further enhanced, and the welfare inequality gap will be reduced even more than the reduction in the nominal wage gap.

**Table 10:** Welfare inequality decomposition from 2005 to 2015, with migration costs eliminated: Wages, rents, and amenities.

Year	(1)	(2)	(3)
2005	0.611	0.611	0.611
	-	-	-
2010	0.715	0.764	0.633
	[0.0044]	[0.0111]	[1.6124]
2015	0.652	0.707	0.467
	[0.0118]	[0.0198]	[3.0243]
2005–2015 change	0.041	0.096	-0.145
Wages	-	-	-
Rents		-	-
Endogenous amenities from resorting of workers			-

Note: The welfare gap was converted to the 2005 wage gap between high- and low-skilled labor.

## 11. Conclusion

From 2005 to 2015, differences in high- and low-skilled migrant labor’s location choice were caused by differences in the spatial distribution of the productivity of such labor. By using a structural spatial equilibrium model that estimated local labor demand, housing supply, labor supply, and amenity supply, I found that local productivity changes led to labor resorting across cities through several channels, and I quantified these effects. Estimates suggest that cities that are disproportionately productive for high-skilled labor attract a larger proportion of high-skilled labor. The rising share of high-skilled labor in these cities leads to higher local productivity, which, in turn, drives up wages for all workers and improves the level of local amenities. A combination of desirable wages and improved amenities has led to a large influx of migrants, pushing up local rents. During this process, the wages of low-skilled labor grew faster, and the real wage gap between differently skilled workers gradually narrowed; simultaneously, the welfare gap between differently skilled workers expanded. Although improvements in the level of amenities can reduce the welfare inequality gap to a certain extent and make it more conducive for low-skilled workers to live in their target cities, the attractiveness of amenities to low-skilled workers is offset by higher rents. High-skilled workers are more capable of paying higher rents; thus, they are more sensitive to the level of urban amenities. Also, migration costs limit access to local amenities for low-skilled labor, allowing high-skilled labor to derive additional utility from more desirable amenities. If migration costs were eliminated, the reduction in welfare inequality between high- and low-skilled labor due to changes in wages, rents, and endogenous amenities would be 25% greater than the reduction in the real wage gap between high- and low-skilled labor, increasing the benefits of low-skilled labor relative to high-skilled labor.

## Supporting information

S1 Appendix (ZIP)

S1 File (ZIP)
