# Supplementary material for: The role amenities play in spatial sorting of migrants and their impact on welfare: Evidence from China
Source: PLoS One. 2023 Feb 16;18(2):e0281669. doi: 10.1371/journal.pone.0281669 (PMC9934390; doi:10.1371/journal.pone.0281669)
Supplement: S1 Appendix — (ZIP) [file pone.0281669.s001.zip › S1_appendix/S1_Appendix.docx]

*This document was created by Word 2016. Other versions of Word may have formatting issues.

**Appendix 1 Comparison of linear and nonlinear dimensionality reduction**

In most real-world problems, data are not uniformly distributed across all dimensions, all data are actually in or near a low-dimensional subspace of a high-dimensional space. A common method of dimensionality reduction is to reduce the dimensionality of the data set by projecting all data vertically onto this subspace. However, linear projection is not always the best way to reduce dimensionality. There may be distortions and flips in the subspace. In the case of the "Swiss Roll" dataset, for example, linear dimensionality reduction simply projects the data onto a plane, and eliminating x3 dimensionality will stack the different layers of the "Swiss rolls" data over each other, as shown in the left panel of Fig 8 (B). However, the actual dimensionality reduction effect we want is to expand the dataset to obtain the dimensionality reduction result in the right panel of Fig 8 (B), so we need to use a non-linear dimensionality reduction method.

| 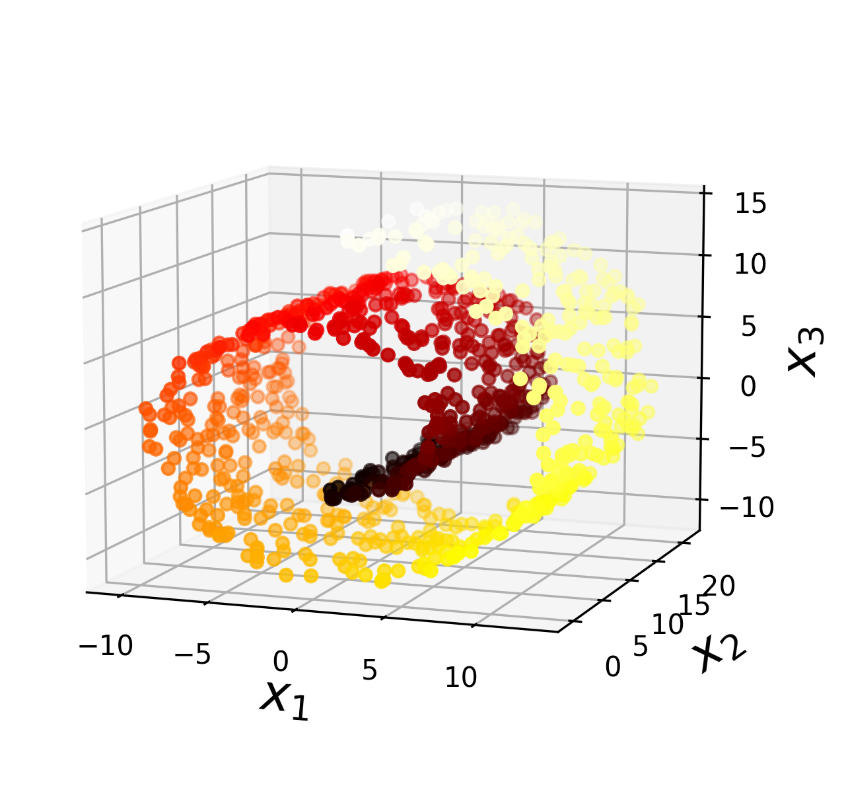  (A) |
| --- |
| 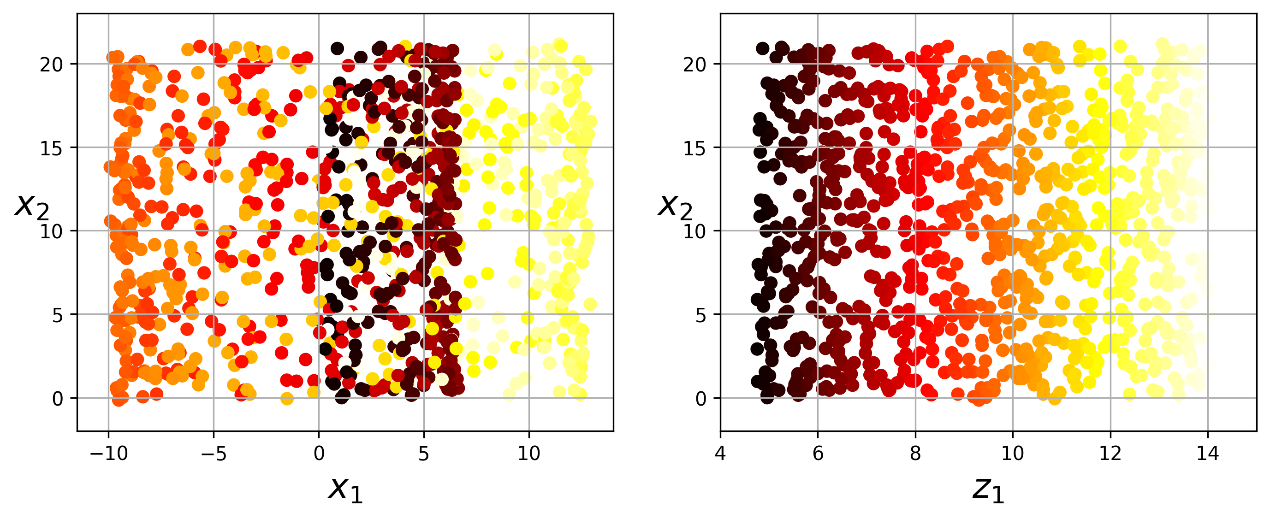  (B) |

**Fig 8 Results of dimensionality reduction of "Swiss Roll" data in different ways**

**Appendix 2 Comparison of the dimensionality reduction effect of AE and PCA**

I use the remaining 6 categories of data in urban amenities except the financial institution category to compare the dimensionality reduction effect of AE and PCA by showing the data reconstructed after dimensionality reduction of AE and PCA. The financial institution data has only one dimension and does not require dimensionality reduction, so it is not involved in the comparison. The closer the reconstructed data is to the real data, the more effective information is retained in the dimensionality reduction process, and the noise in the data is better removed. In Fig 9, the blue curve represents the real data, and the red curve represents the reconstructed data. The more overlapping parts of the two, the closer the data reconstructed from the dimensionality reduction result is to the real data. In other words, the dimensionality reduction effect is better.

| Traffic Index, AE |
| --- |
| 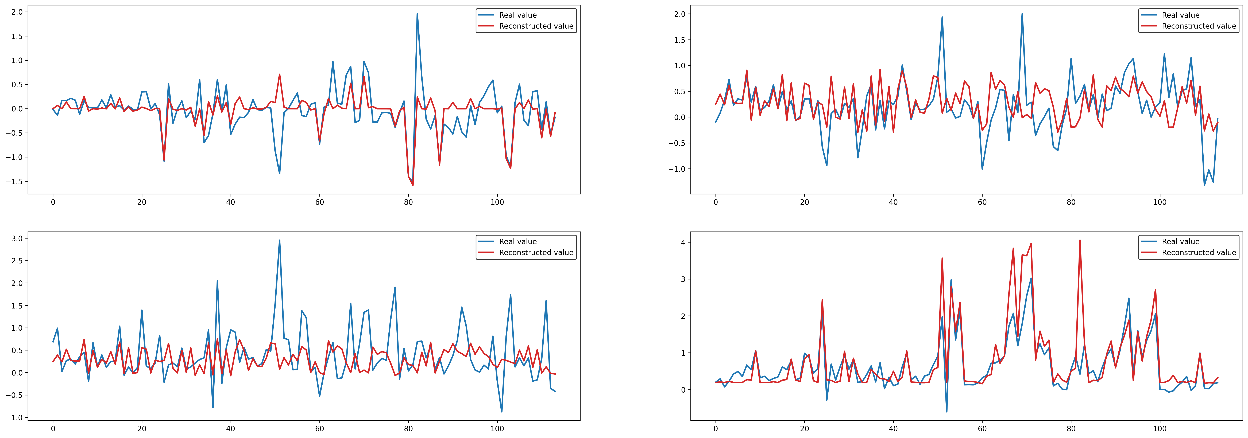 |
| Traffic Index, PCA |
| 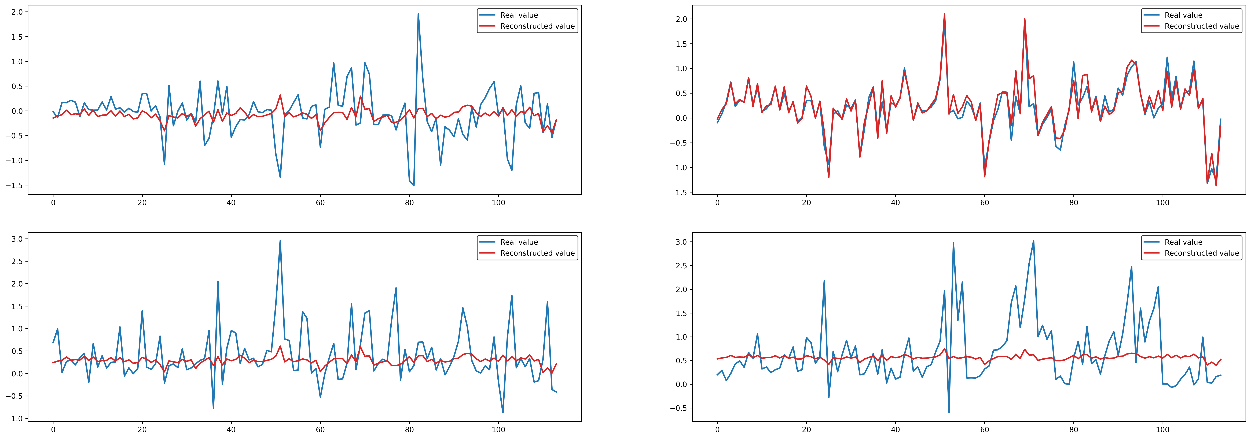 |
| Education Index, AE |
| 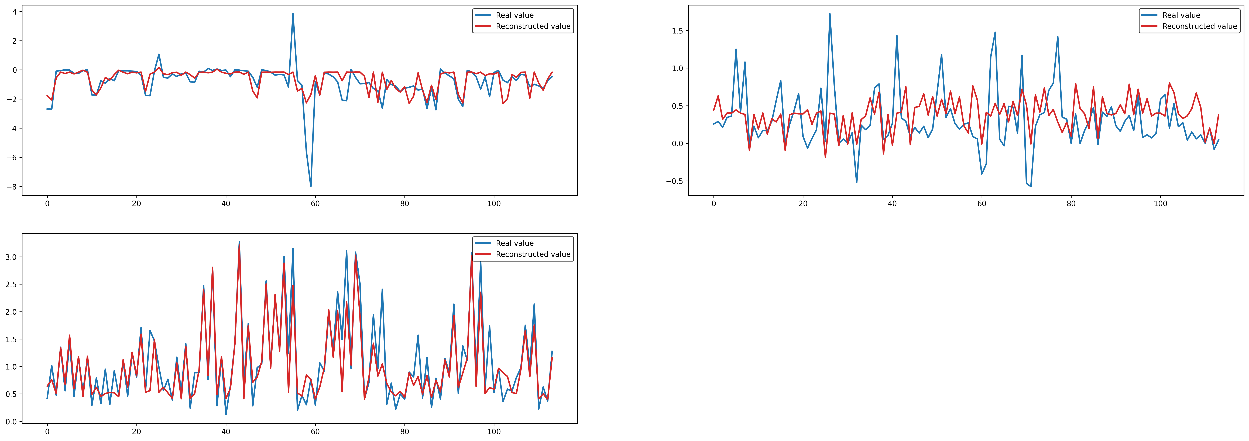 |
| Education Index, PCA |
| 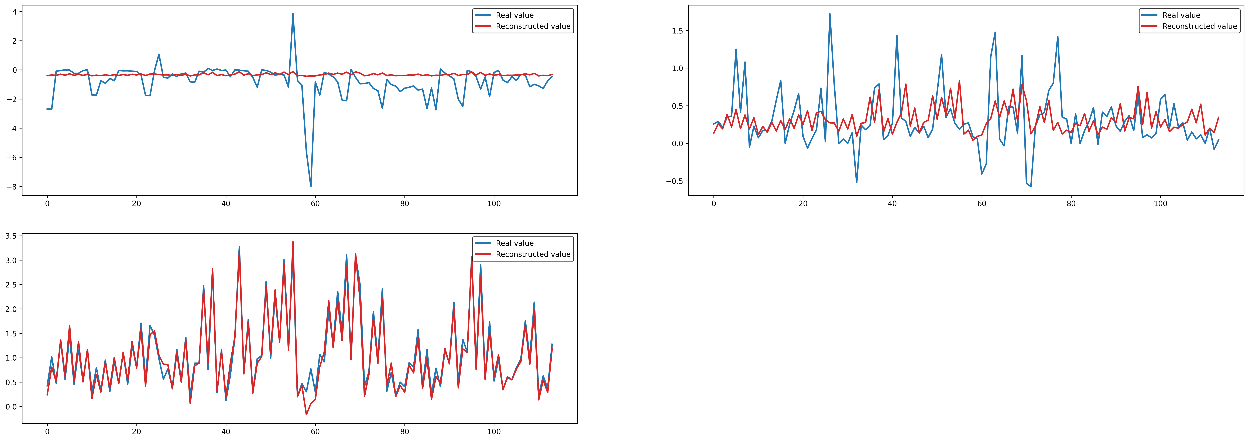 |
| Employment Index, AE |
| 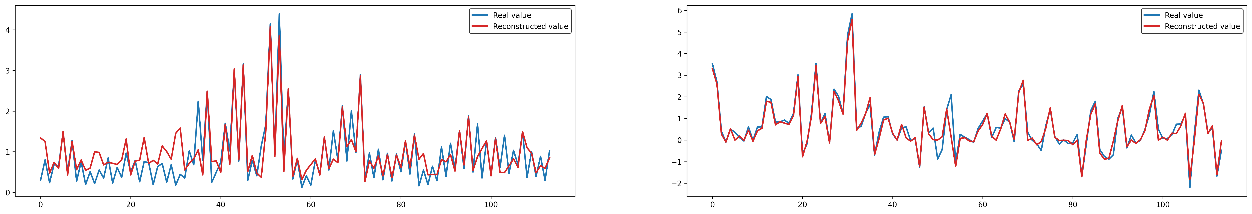 |
| Employment Index, PCA |
| 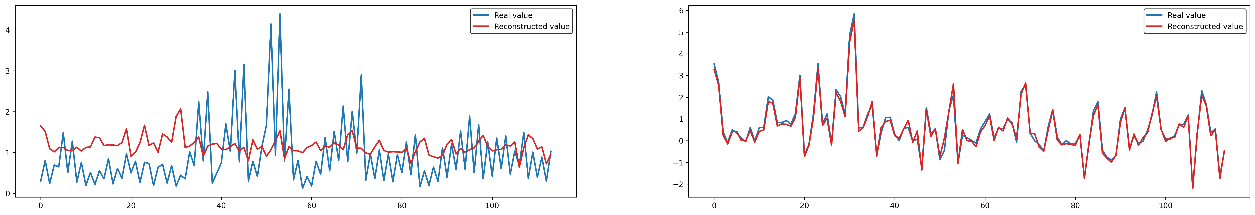 |
| Cultural Index, AE |
| 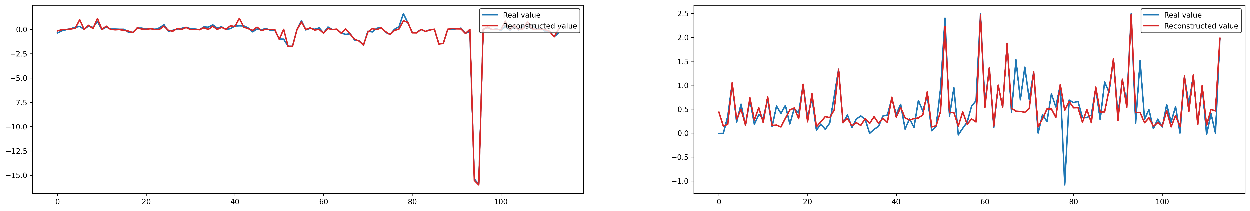 |
| Cultural Index, PCA |
| 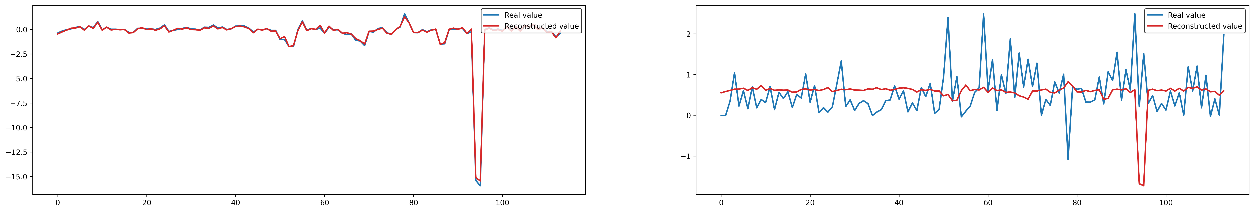 |
| Environmental Index, AE |
| 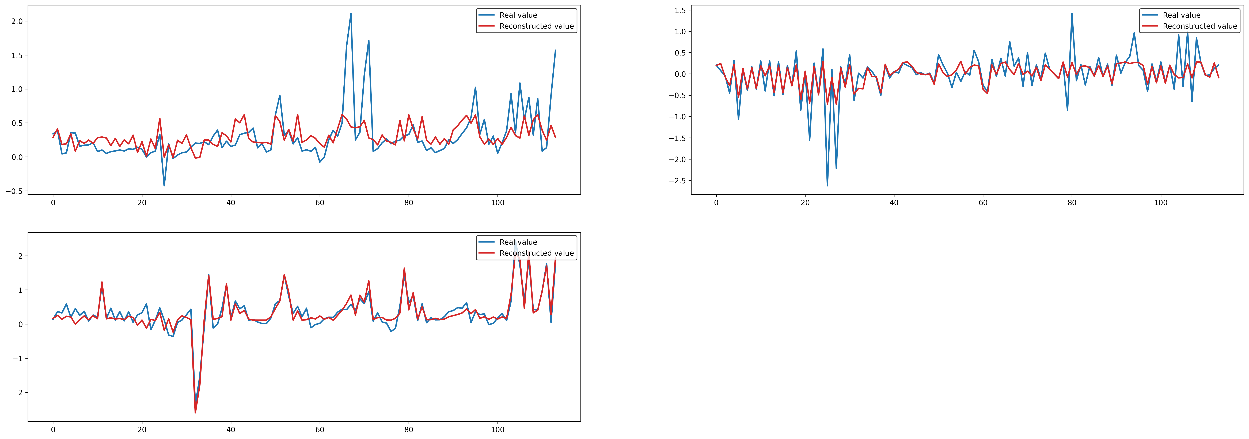 |
| Environmental Index, PCA |
| 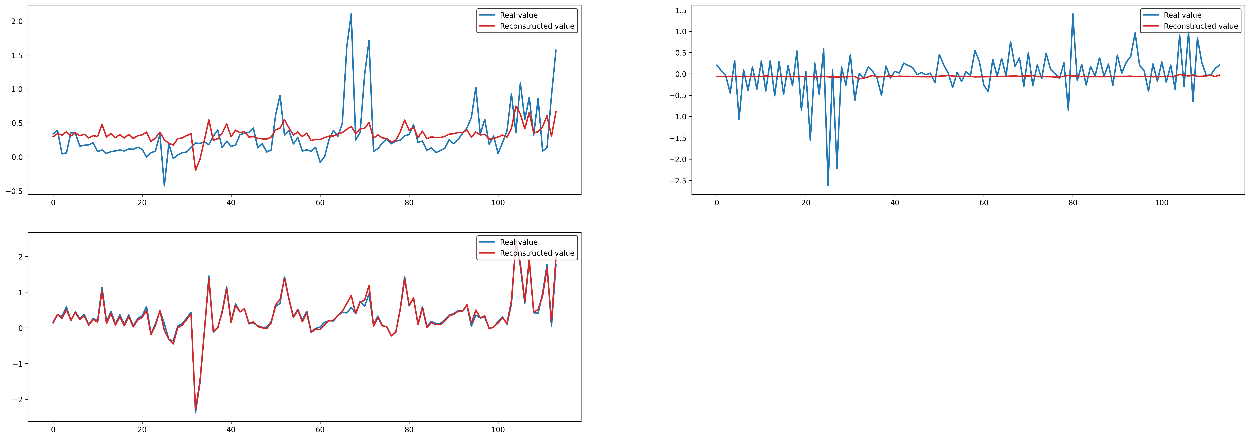 |
| Medical Index, AE |
| 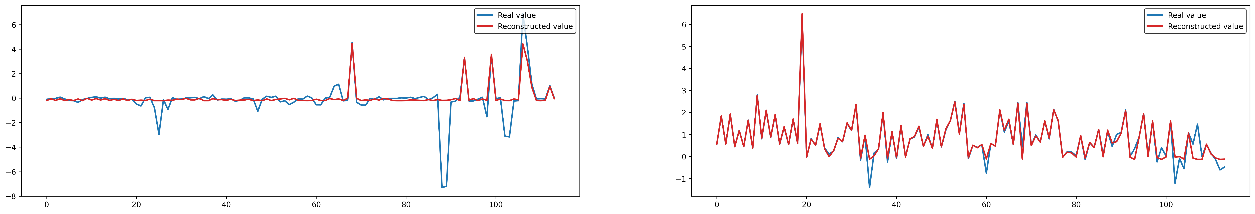 |
| Medical Index, PCA |
| 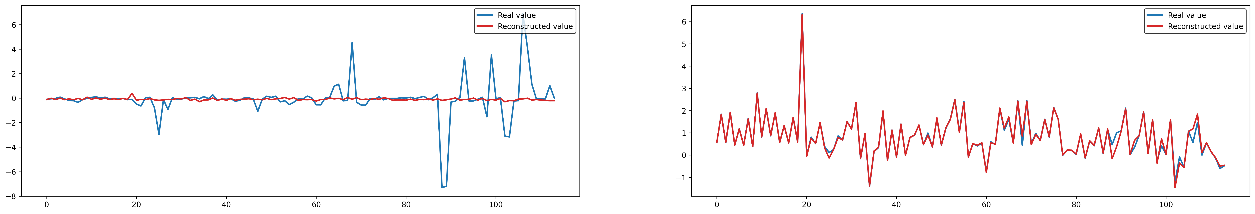 |

**Fig 9 Comparison of dimensionality reduction effect of AE and PCA**

**Appendix 3 Test of the mapping relationship between the hidden layer and the output layer**

This section tests whether the two standard Gaussian distributions normalized by the output and hidden layer in the decoder are uniform monotonic mappings on both sides of the mean. Take the 2015 data as an example (the results of 2005 and 2010 are the same as those of 2015, and the graphs are very similar). The normalized hidden layer obeys a standard Gaussian distribution with values essentially in the (-2,4) interval. On the normalized hidden layer, I chose data points in the interval with small distance from the minimum value point as data generation points. Here I choose to control the precision to three decimal places (the distance between data generation points can be adjusted to other values such as 0.01 or 0.0001, and the conclusion is still robust), that is, data generation points are selected at 0.001 distance intervals. These data generating points are then sequentially numbered and put into the VAE decoder to generate reconstructed data. The value of the reconstructed data is used as the X-axis and the sequential numbering of the reconstructed data points is used as the Y-axis to draw a graph. If the two standard Gaussian distributions of the output layer and the hidden layer are uniformly monotonically mapped on both sides of the mean, then the graphs to the left and right of point 0 on the X-axis (the mean of the standard Gaussian distribution is 0) should be two straight lines, respectively. If the two standard Gaussian distributions of the output and hidden layer are not uniformly monotonically mapped on both sides of the mean, then the graphs to the left and right of point 0 on the X-axis will be curves or jump discontinuities instead of two straight lines. Fig 10 shows the results of testing the mapping relationship between the hidden and output layer for high skill labor (A) and low skill labor (B) in the VAE in 2015. The results show that the two standard Gaussian distributions in the decoder are uniformly monotonic mappings on both sides of the mean, which means that the mapping relationship between the hidden layer and the output layer is consistent with Fig 11 (C).

| 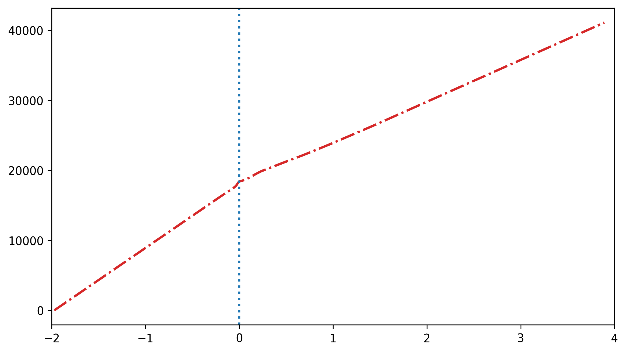 | 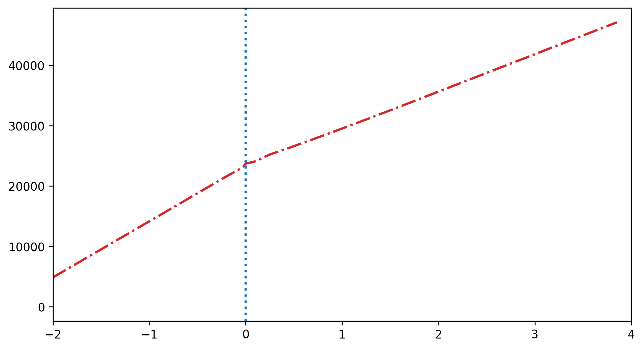 |
| --- | --- |
| (A) High skill labor | (B) Low skill labor |

**Fig 10 The results of examining the mapping relationship between the hidden layer and the output layer**

| 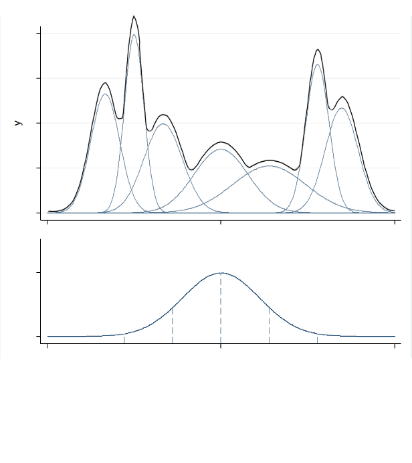 | 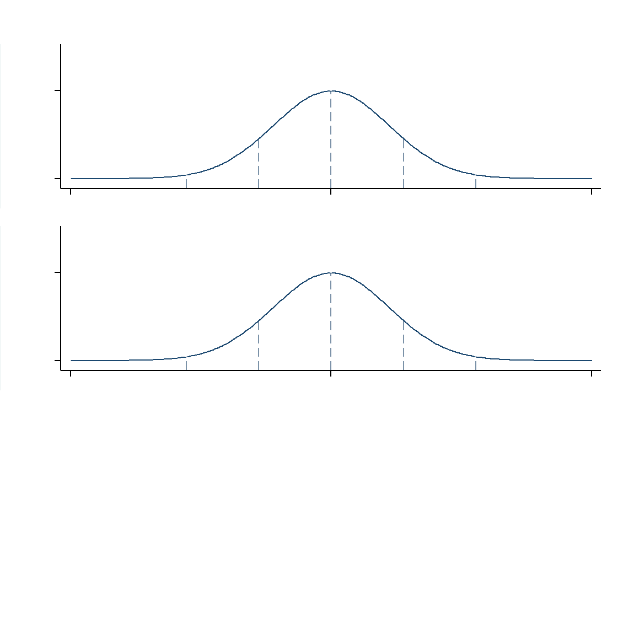 | 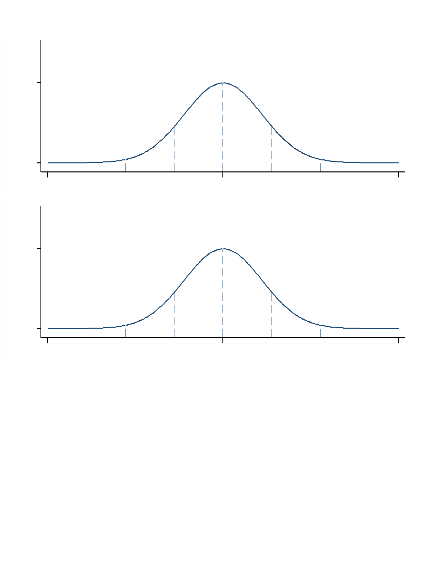 |
| --- | --- | --- |
| (A) Possible mapping relationships between hidden layer and output layer in Gaussian Mixture Model | (B) Imperfect mapping between the generated data and the latent space when both the output and hidden layer are one-dimensional and obey the standard Gaussian distribution | (C) The ideal mapping relationship between the generated data and the latent space when both the output layer and the hidden layer are one-dimensional and obey the standard Gaussian distribution |

**Fig 11 Mapping relationships of Gaussian Mixture Model**
