# Supplementary figures and images for: The role amenities play in spatial sorting of migrants and their impact on welfare: Evidence from China
Source: PLoS One. 2023 Feb 16;18(2):e0281669. doi: 10.1371/journal.pone.0281669 (PMC9934390; doi:10.1371/journal.pone.0281669)

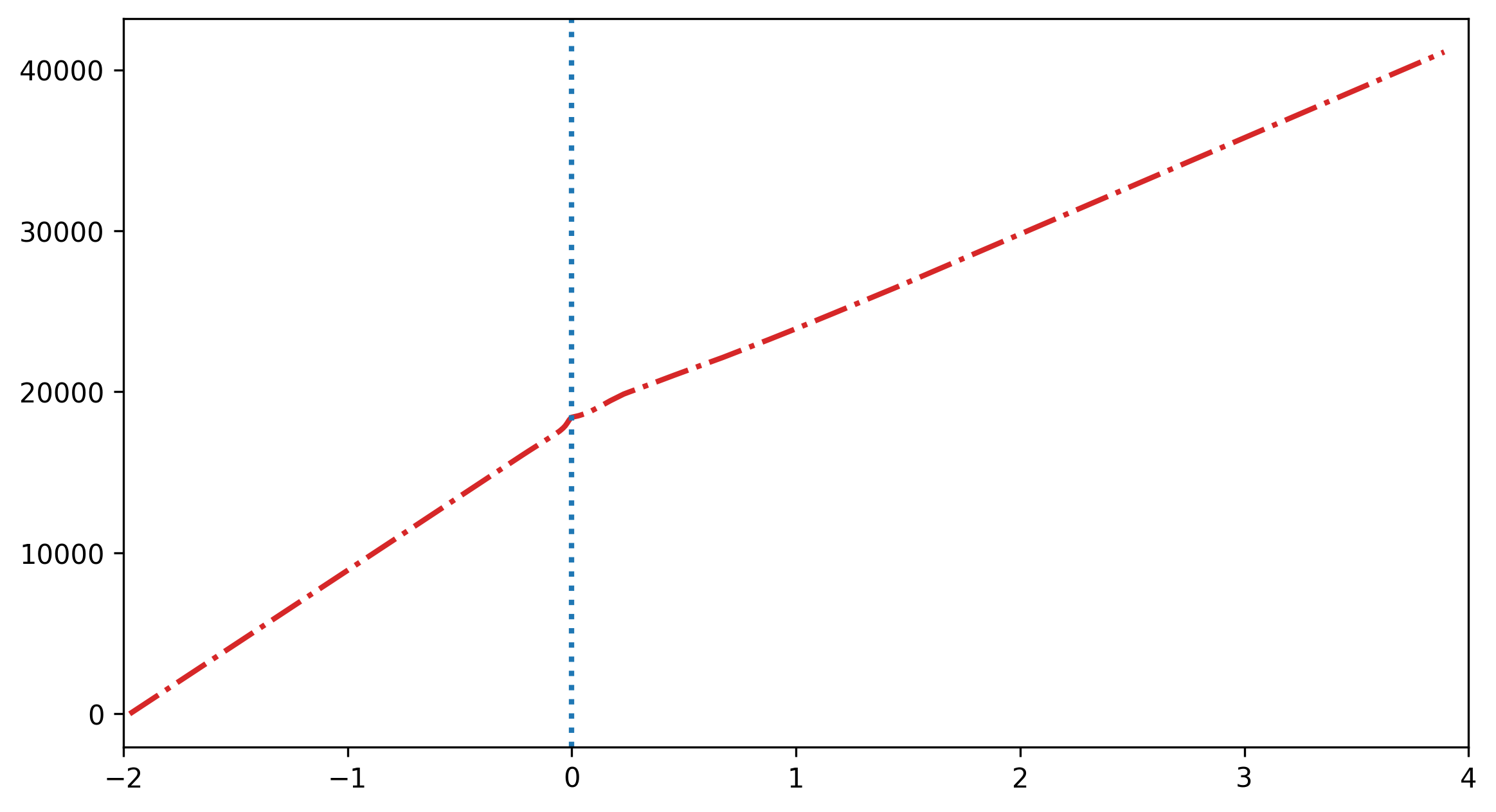

Supplement: S1 Appendix — (ZIP) [file pone.0281669.s001.zip › S1_appendix/S1_Figs_of_appendix/Fig 10A.tif]

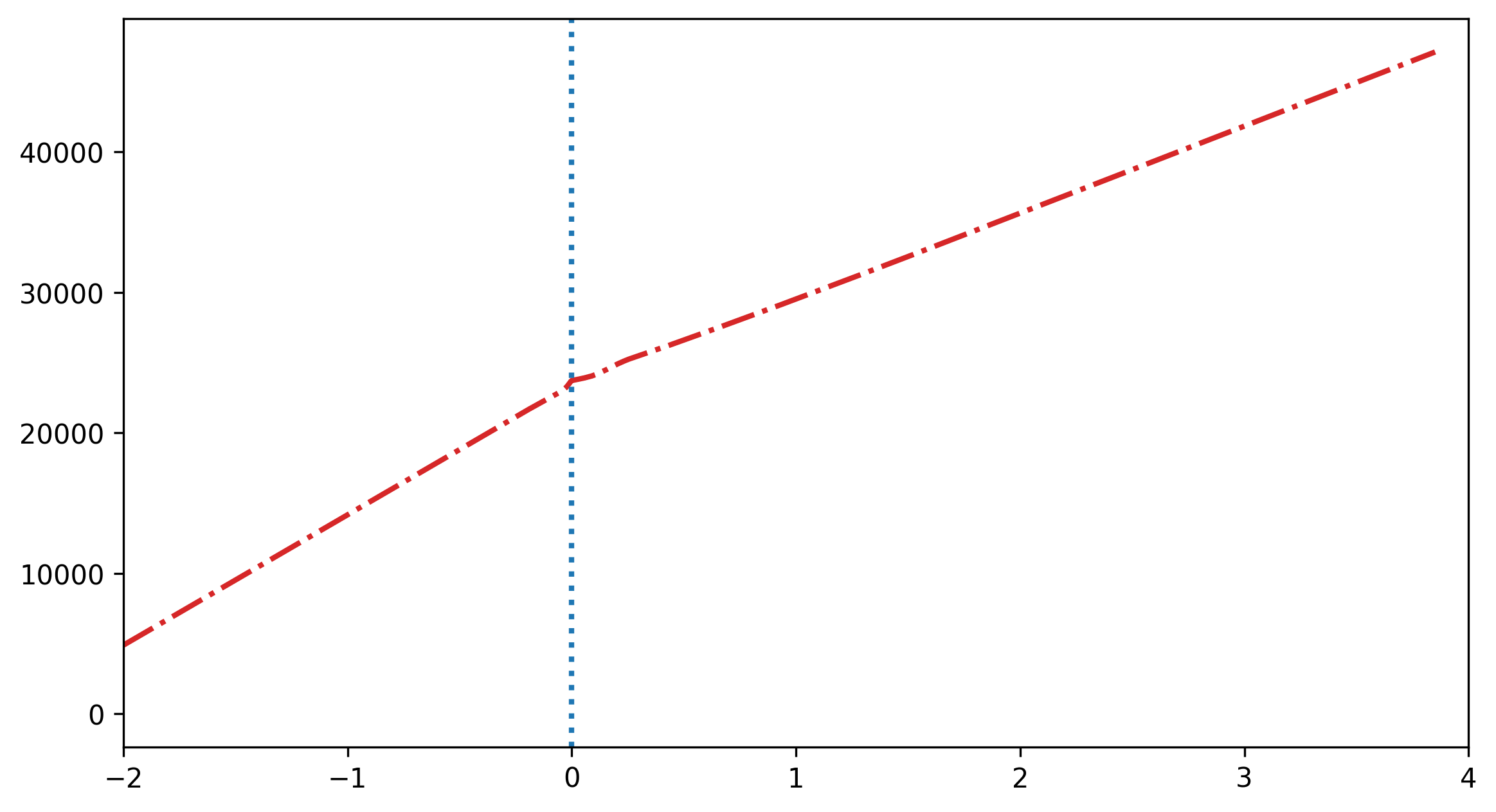

Supplement: S1 Appendix — (ZIP) [file pone.0281669.s001.zip › S1_appendix/S1_Figs_of_appendix/Fig 10B.tif]

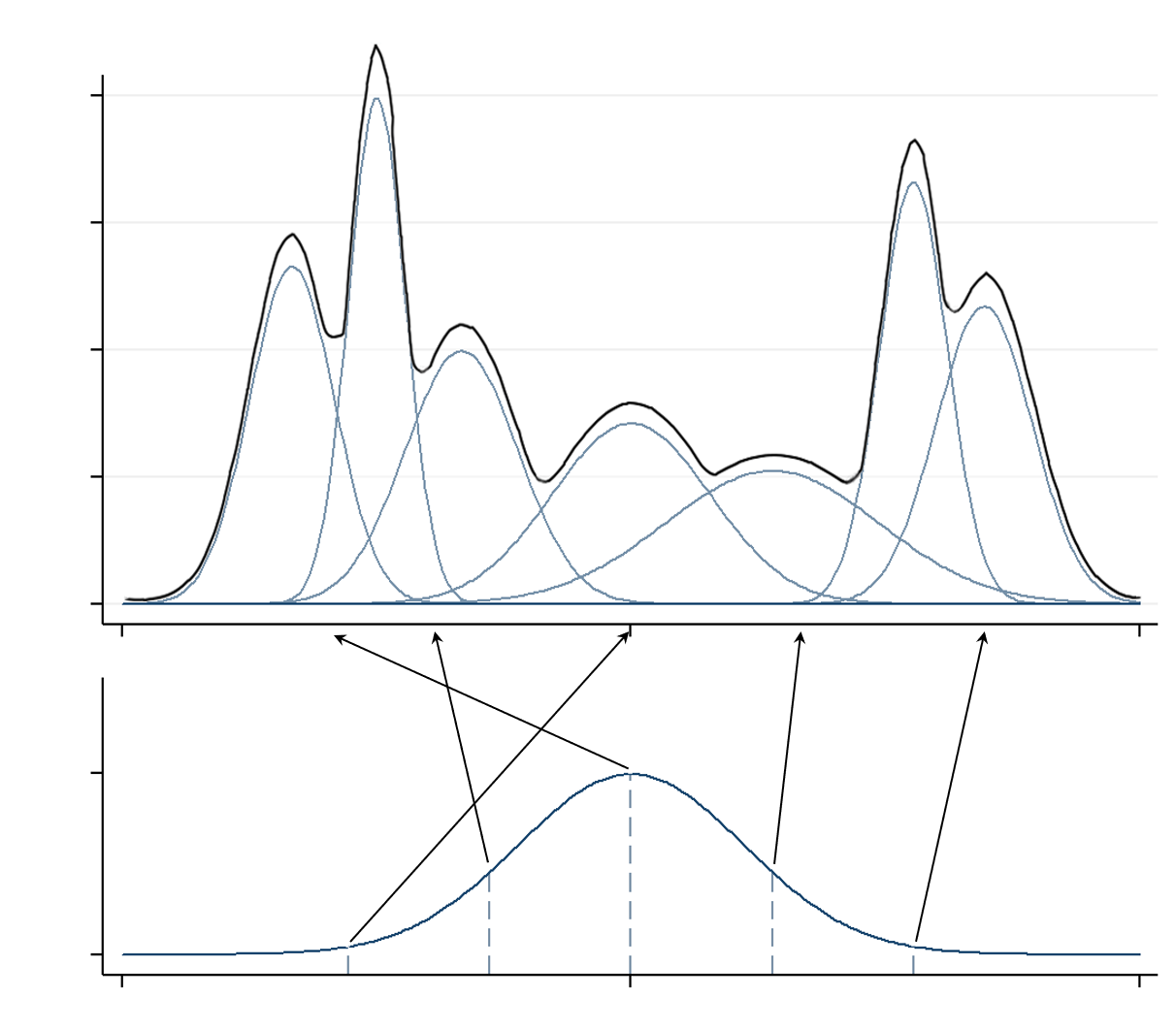

Supplement: S1 Appendix — (ZIP) [file pone.0281669.s001.zip › S1_appendix/S1_Figs_of_appendix/Fig 11A.tif]

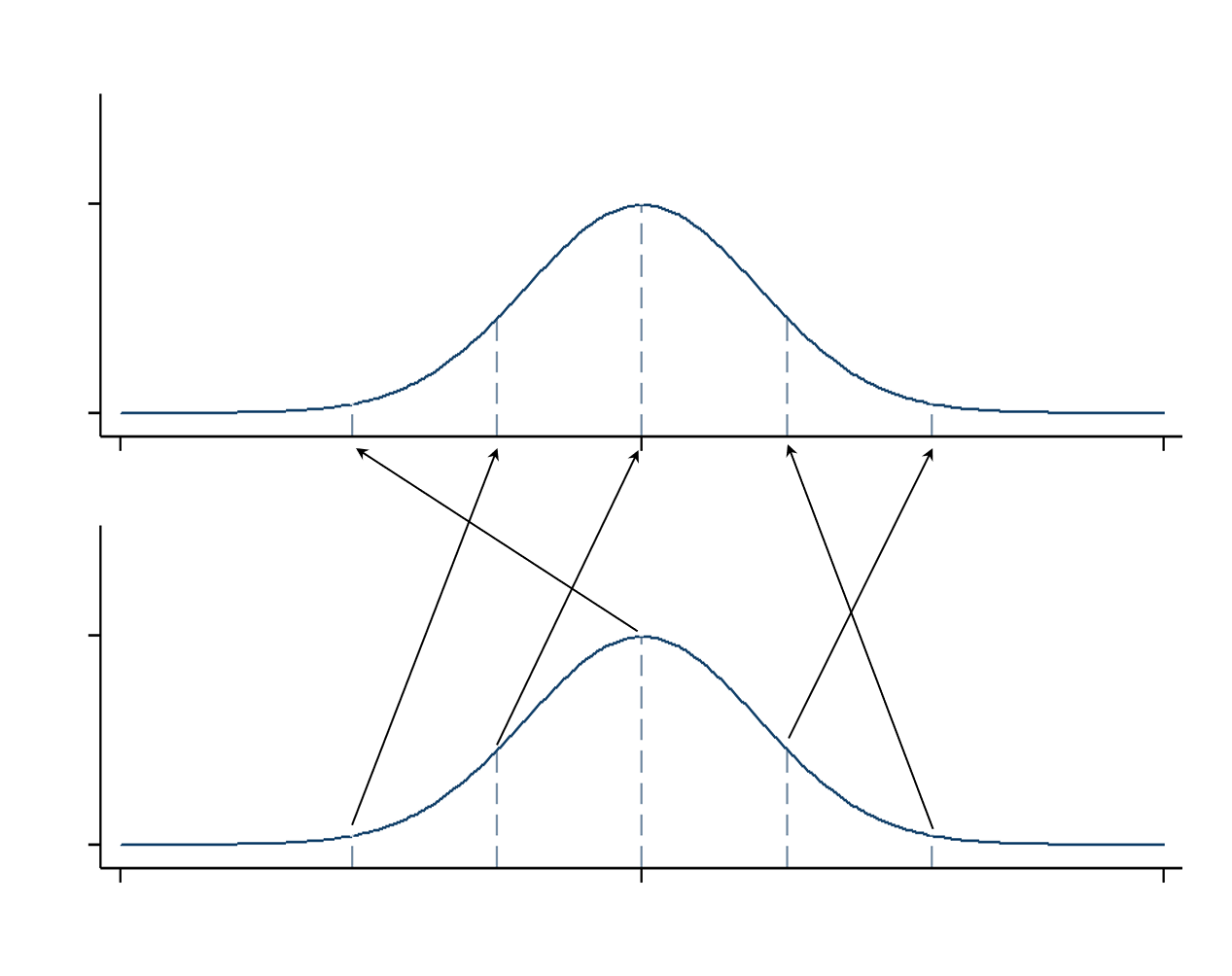

Supplement: S1 Appendix — (ZIP) [file pone.0281669.s001.zip › S1_appendix/S1_Figs_of_appendix/Fig 11B.tif]

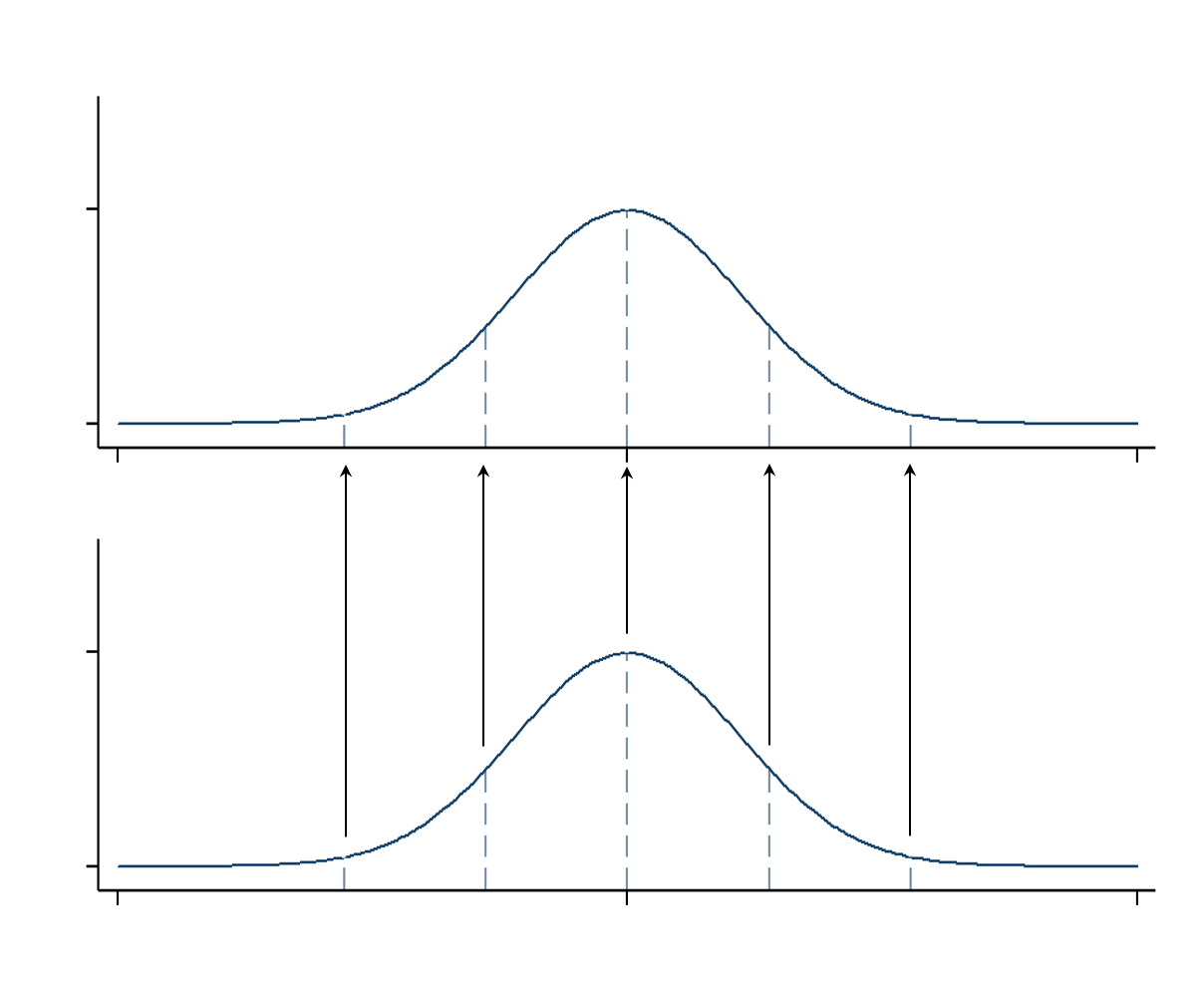

Supplement: S1 Appendix — (ZIP) [file pone.0281669.s001.zip › S1_appendix/S1_Figs_of_appendix/Fig 11C.tif]

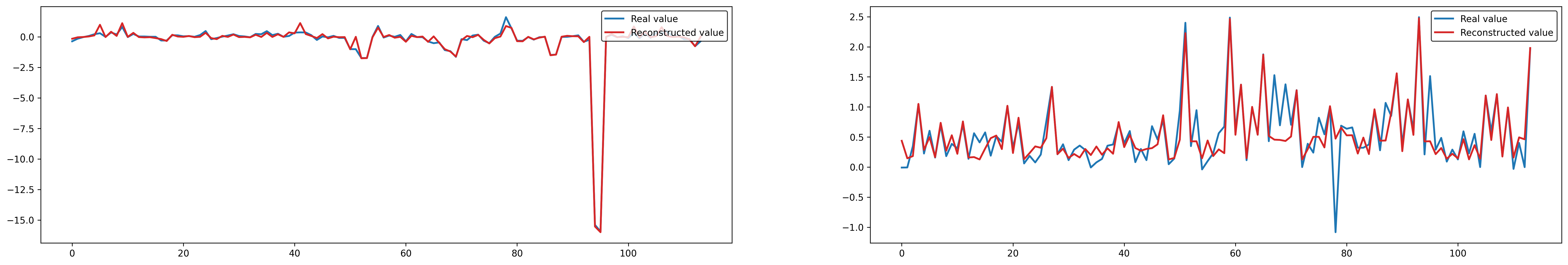

Supplement: S1 Appendix — (ZIP) [file pone.0281669.s001.zip › S1_appendix/S1_Figs_of_appendix/Fig 9_Reconstructed_culture_AE.tif]

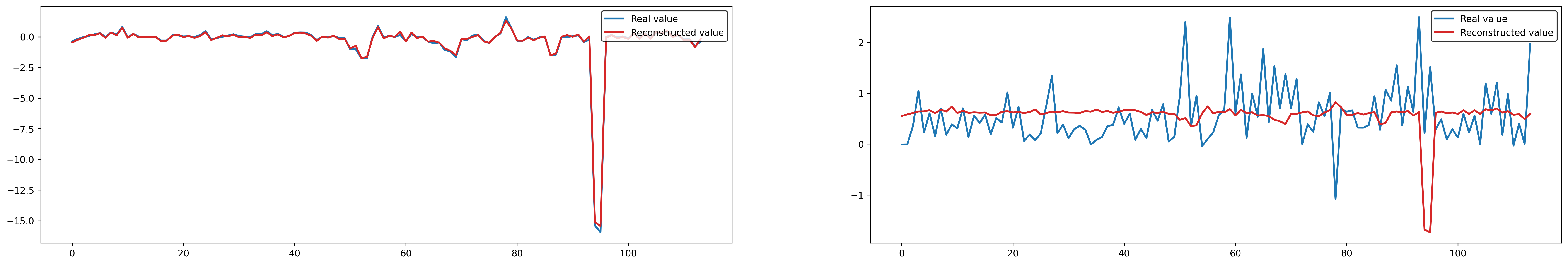

Supplement: S1 Appendix — (ZIP) [file pone.0281669.s001.zip › S1_appendix/S1_Figs_of_appendix/Fig 9_Reconstructed_culture_PCA.tif]

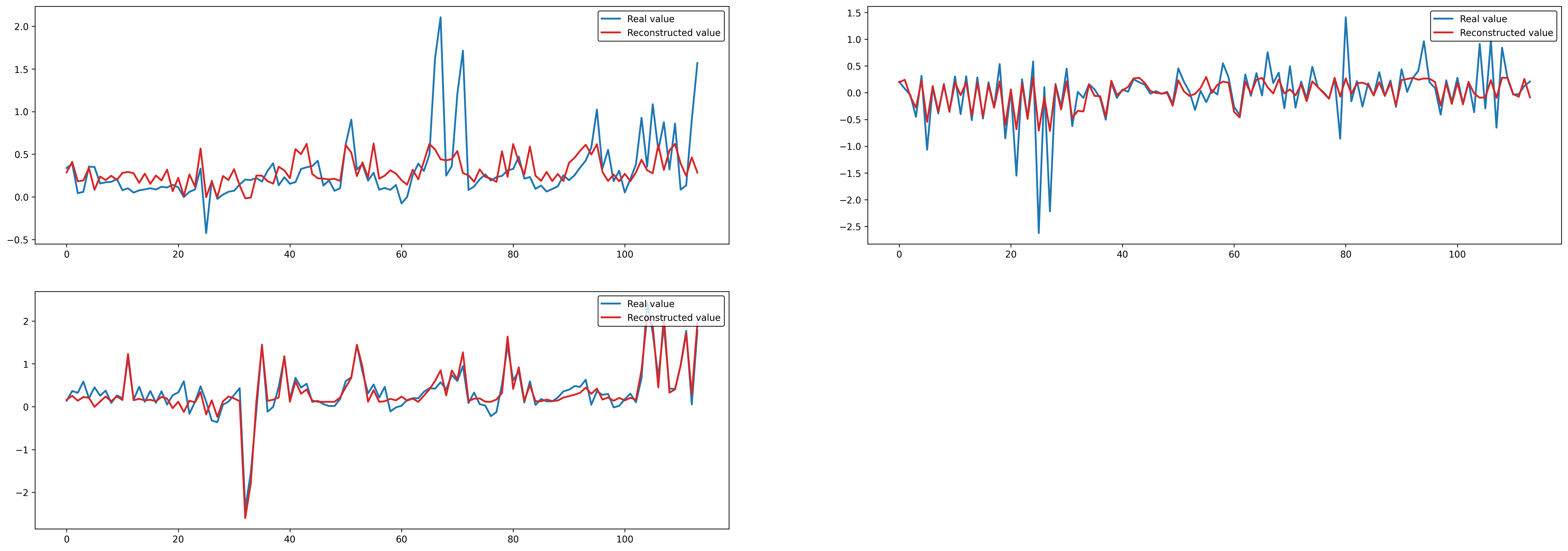

Supplement: S1 Appendix — (ZIP) [file pone.0281669.s001.zip › S1_appendix/S1_Figs_of_appendix/Fig 9_Reconstructed_environment_AE.tif]

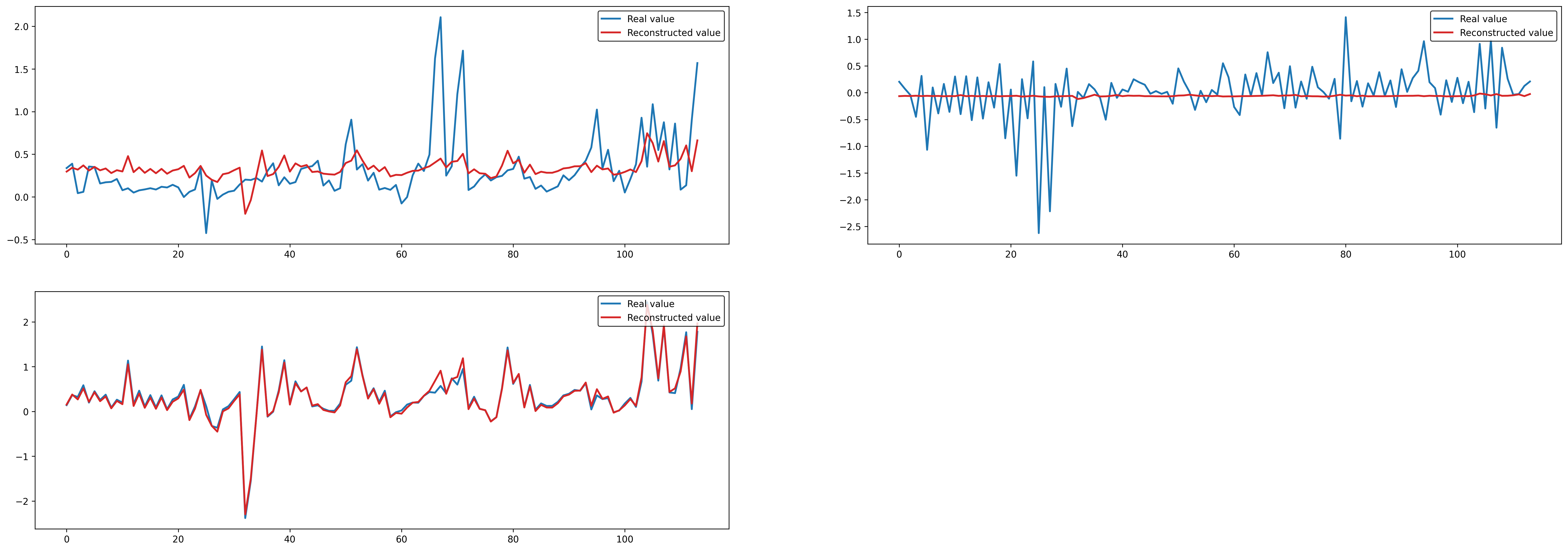

Supplement: S1 Appendix — (ZIP) [file pone.0281669.s001.zip › S1_appendix/S1_Figs_of_appendix/Fig 9_Reconstructed_environment_PCA.tif]

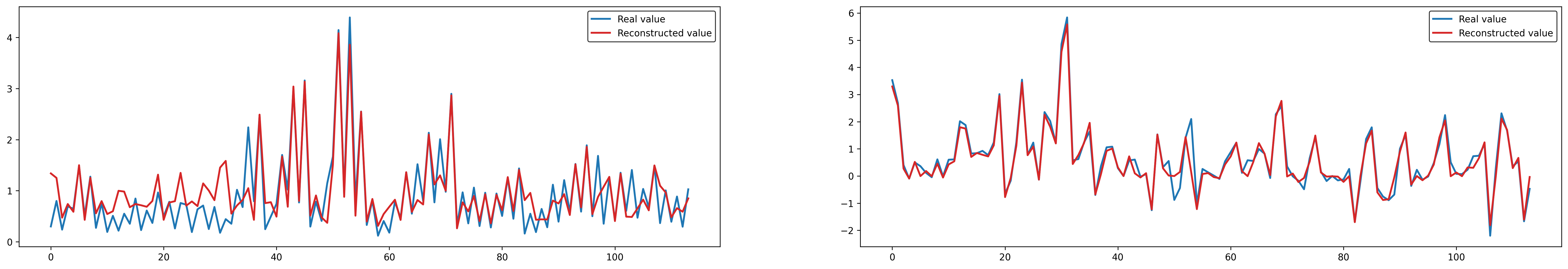

Supplement: S1 Appendix — (ZIP) [file pone.0281669.s001.zip › S1_appendix/S1_Figs_of_appendix/Fig 9_Reconstructed_jobs_AE.tif]

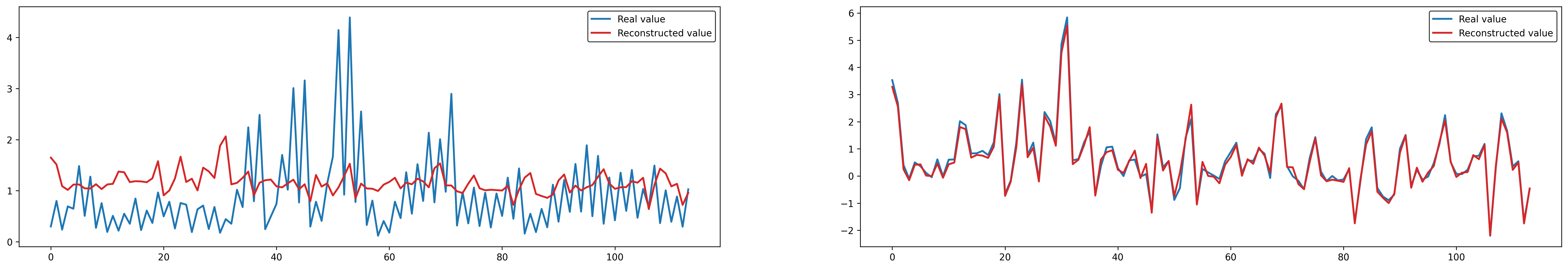

Supplement: S1 Appendix — (ZIP) [file pone.0281669.s001.zip › S1_appendix/S1_Figs_of_appendix/Fig 9_Reconstructed_jobs_PCA.tif]

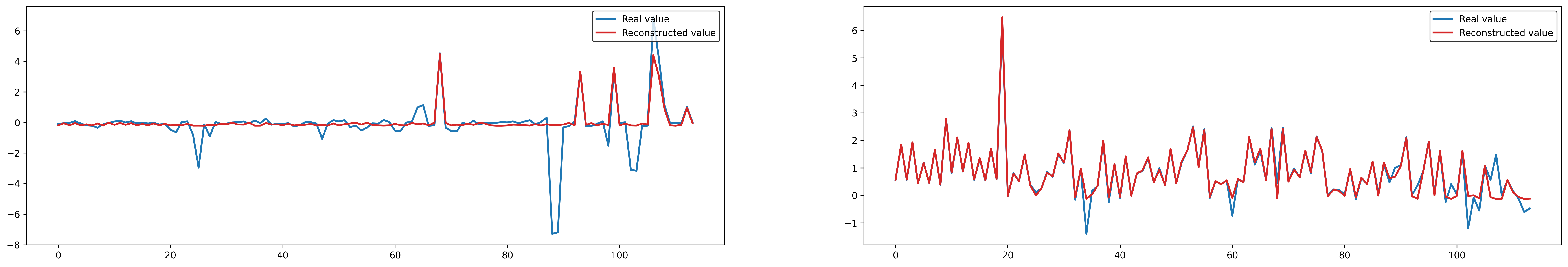

Supplement: S1 Appendix — (ZIP) [file pone.0281669.s001.zip › S1_appendix/S1_Figs_of_appendix/Fig 9_Reconstructed_medical_AE.tif]

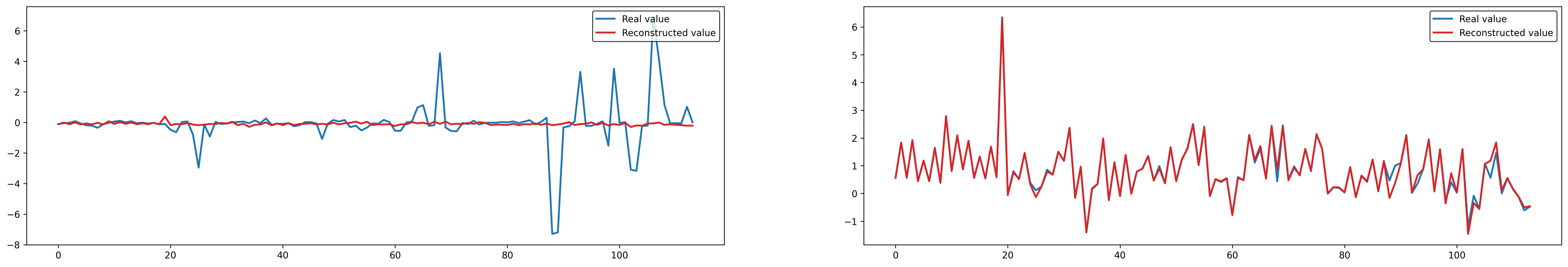

Supplement: S1 Appendix — (ZIP) [file pone.0281669.s001.zip › S1_appendix/S1_Figs_of_appendix/Fig 9_Reconstructed_medical_PCA.tif]

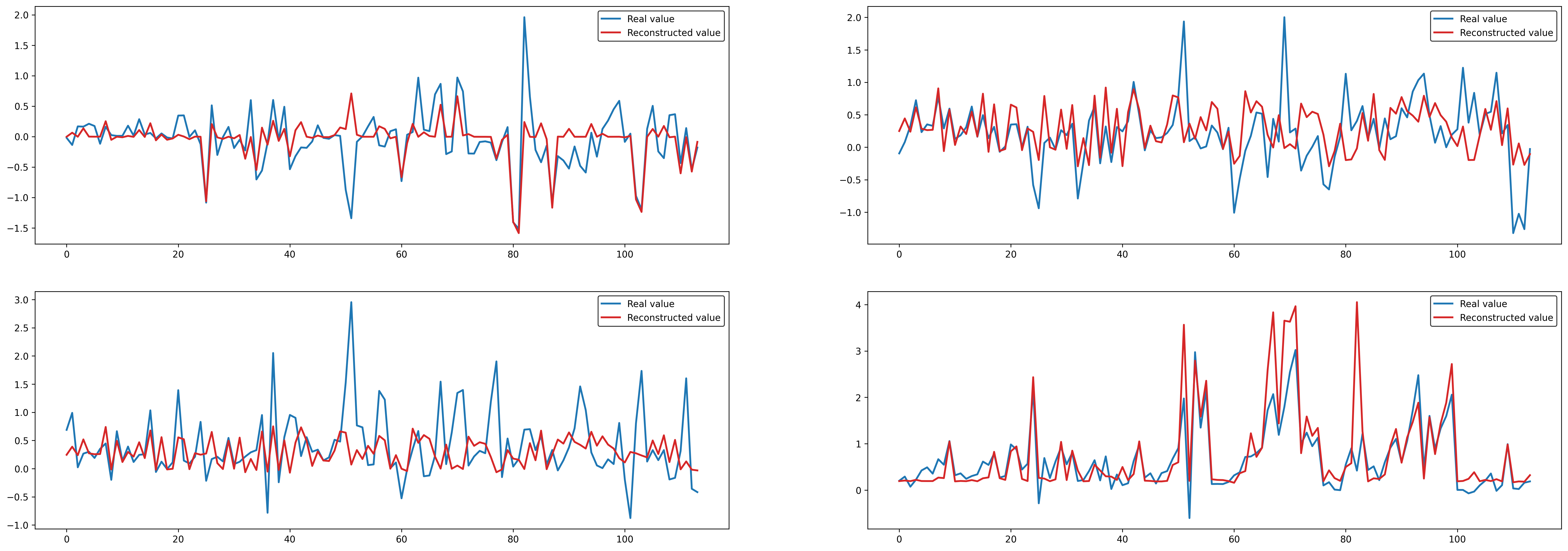

Supplement: S1 Appendix — (ZIP) [file pone.0281669.s001.zip › S1_appendix/S1_Figs_of_appendix/Fig 9_Reconstructed_road_AE.tif]

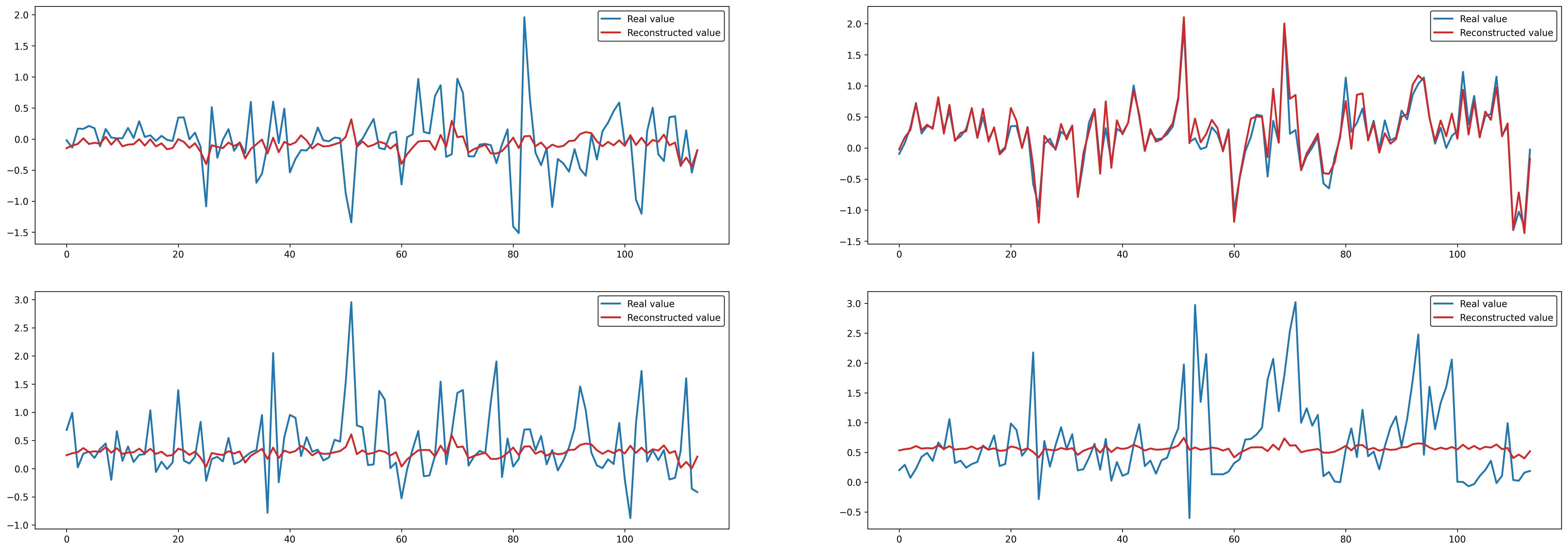

Supplement: S1 Appendix — (ZIP) [file pone.0281669.s001.zip › S1_appendix/S1_Figs_of_appendix/Fig 9_Reconstructed_road_PCA.tif]

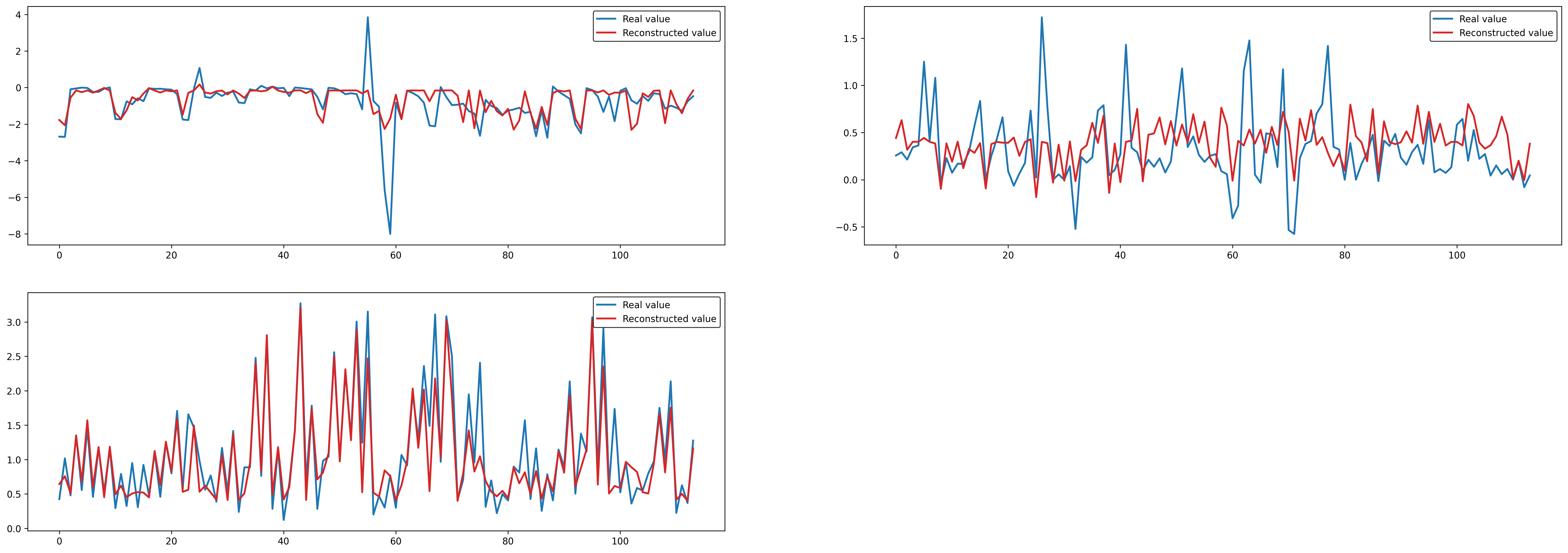

Supplement: S1 Appendix — (ZIP) [file pone.0281669.s001.zip › S1_appendix/S1_Figs_of_appendix/Fig 9_Reconstructed_school_AE.tif]

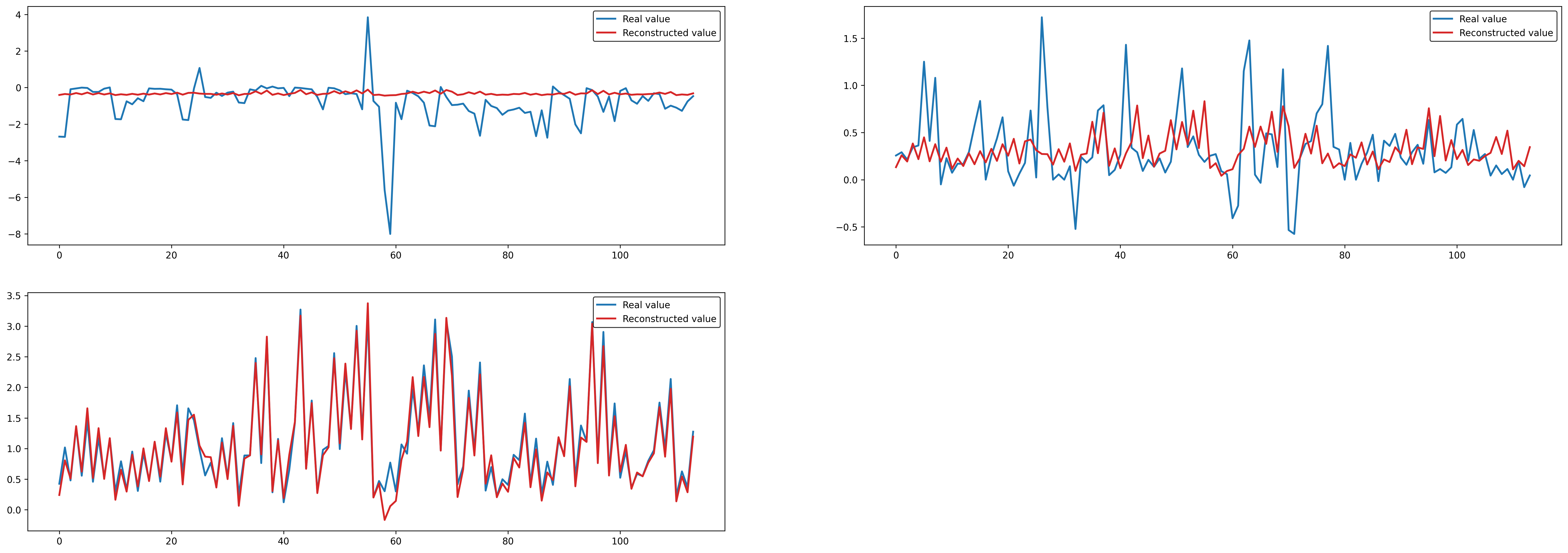

Supplement: S1 Appendix — (ZIP) [file pone.0281669.s001.zip › S1_appendix/S1_Figs_of_appendix/Fig 9_Reconstructed_school_PCA.tif]

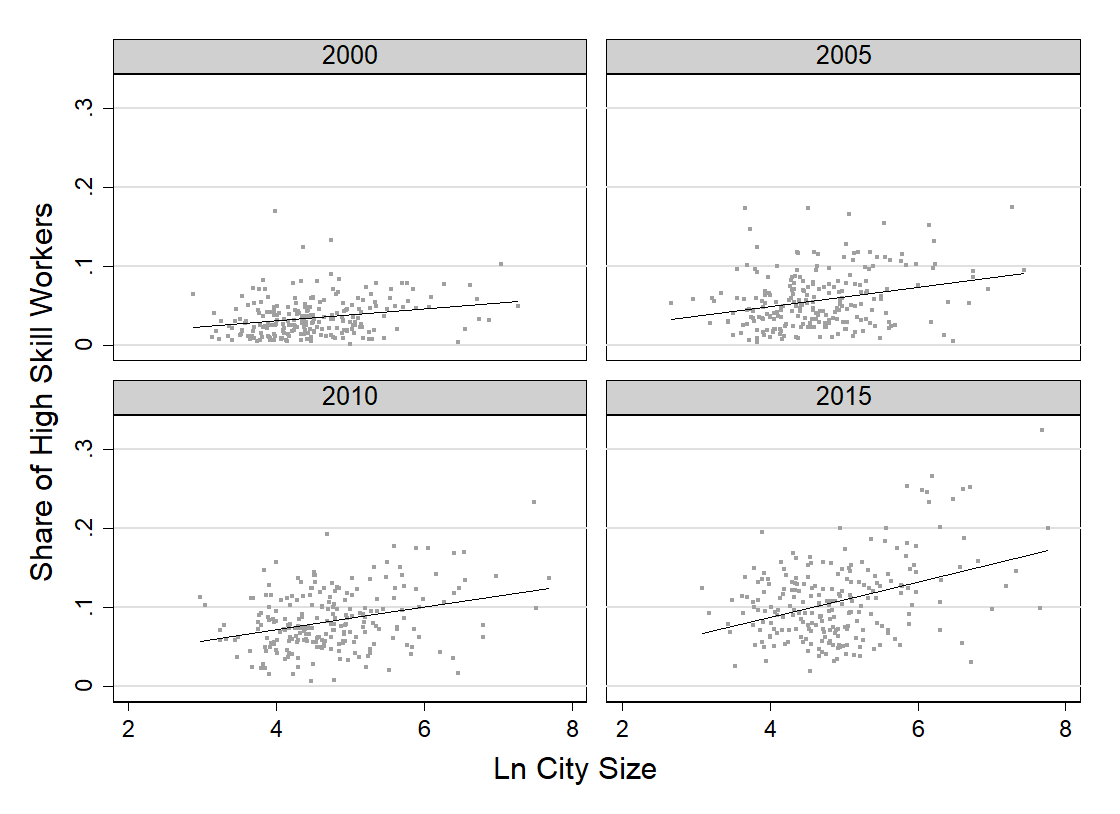

Supplement: S1 File — (ZIP) [file pone.0281669.s002.zip › S2_replication/S2_replication_data_and_program/Fig2.tif]

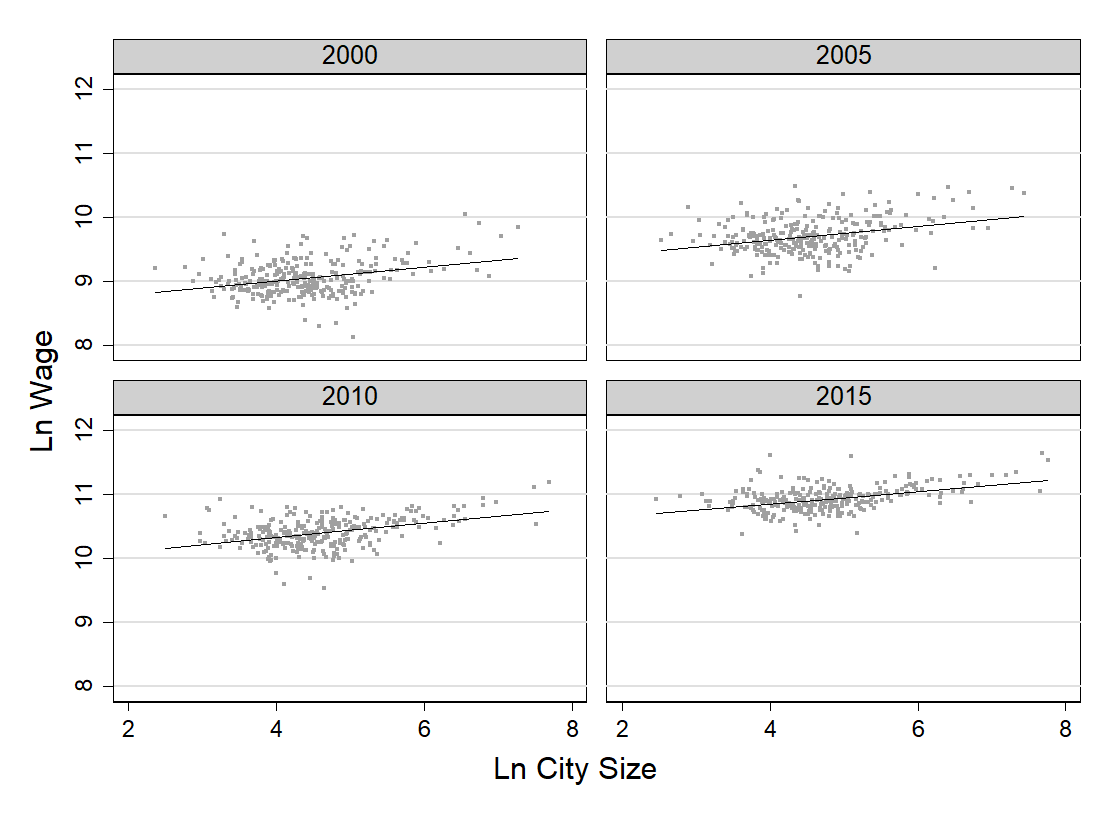

Supplement: S1 File — (ZIP) [file pone.0281669.s002.zip › S2_replication/S2_replication_data_and_program/Fig3.tif]

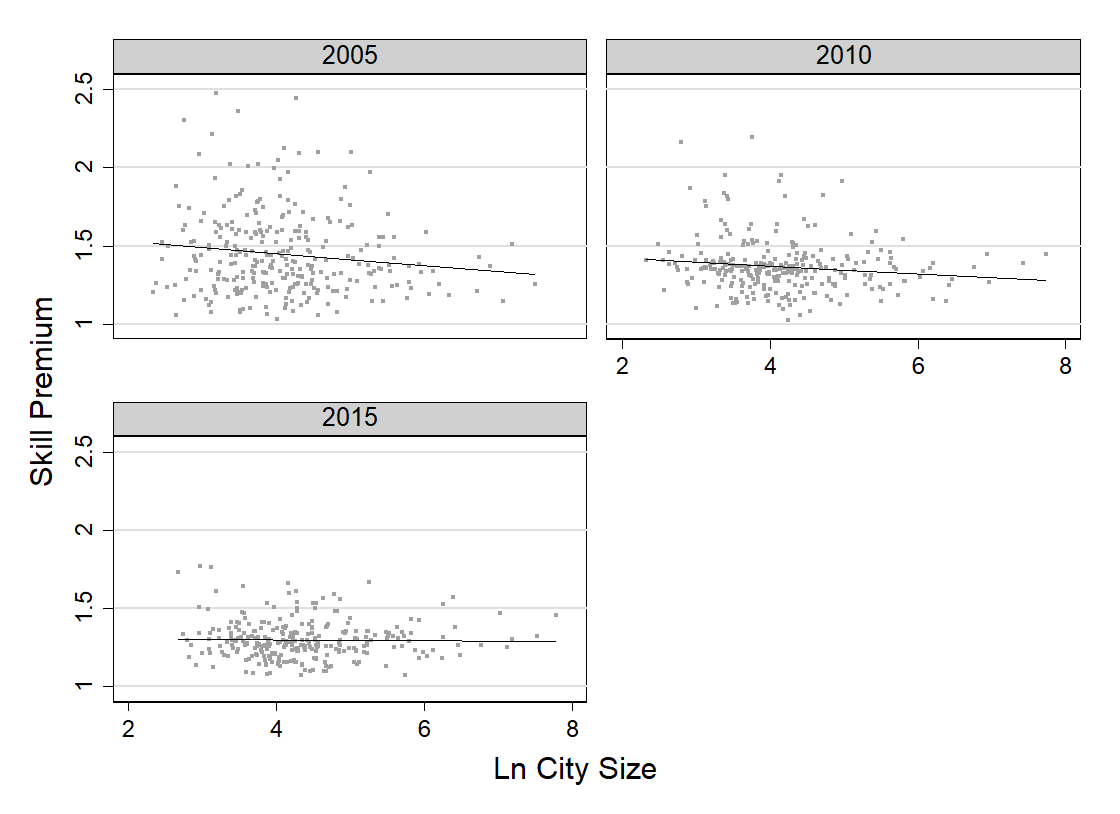

Supplement: S1 File — (ZIP) [file pone.0281669.s002.zip › S2_replication/S2_replication_data_and_program/Fig4.tif]

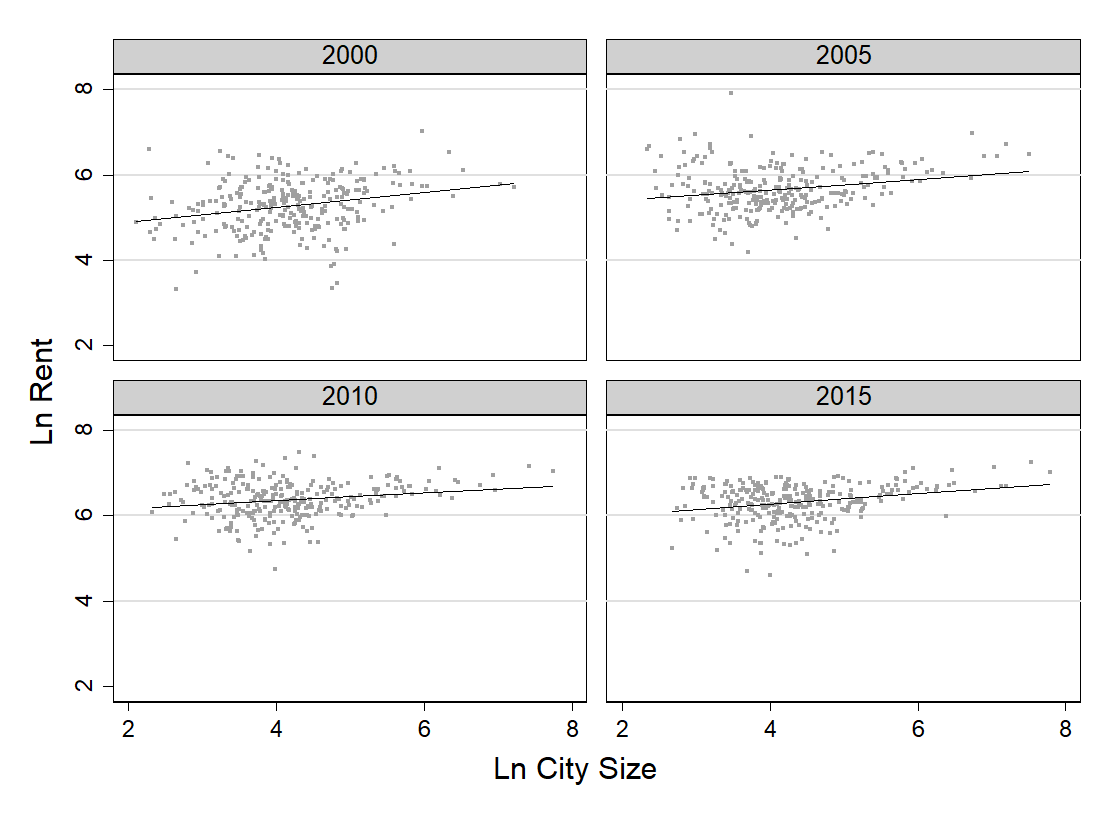

Supplement: S1 File — (ZIP) [file pone.0281669.s002.zip › S2_replication/S2_replication_data_and_program/Fig5.tif]

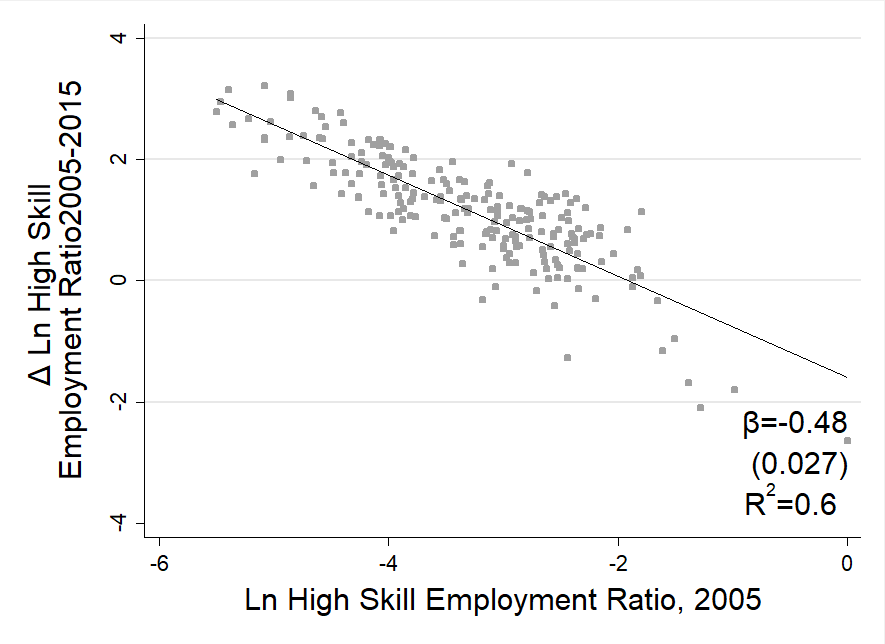

Supplement: S1 File — (ZIP) [file pone.0281669.s002.zip › S2_replication/S2_replication_data_and_program/Fig6a.tif]

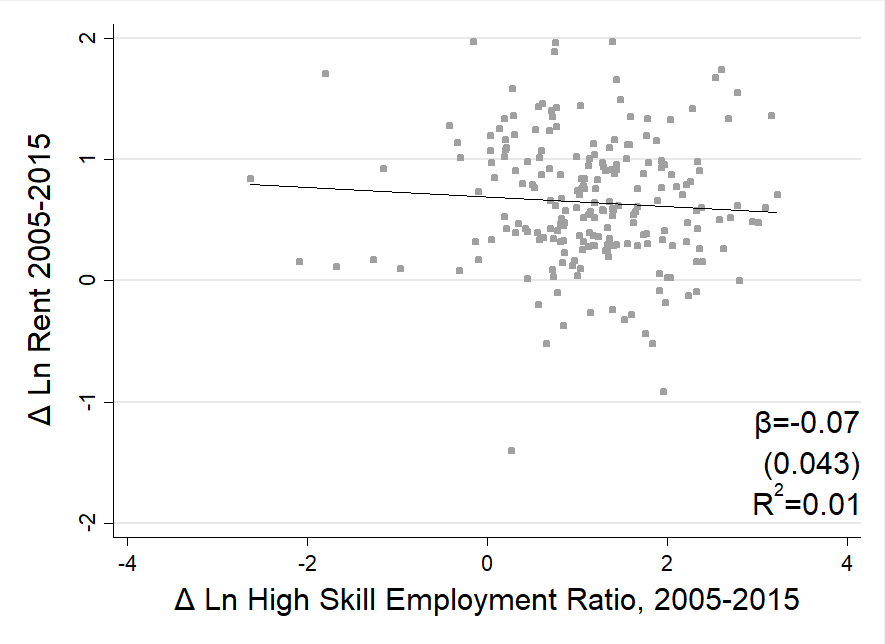

Supplement: S1 File — (ZIP) [file pone.0281669.s002.zip › S2_replication/S2_replication_data_and_program/Fig6b.tif]

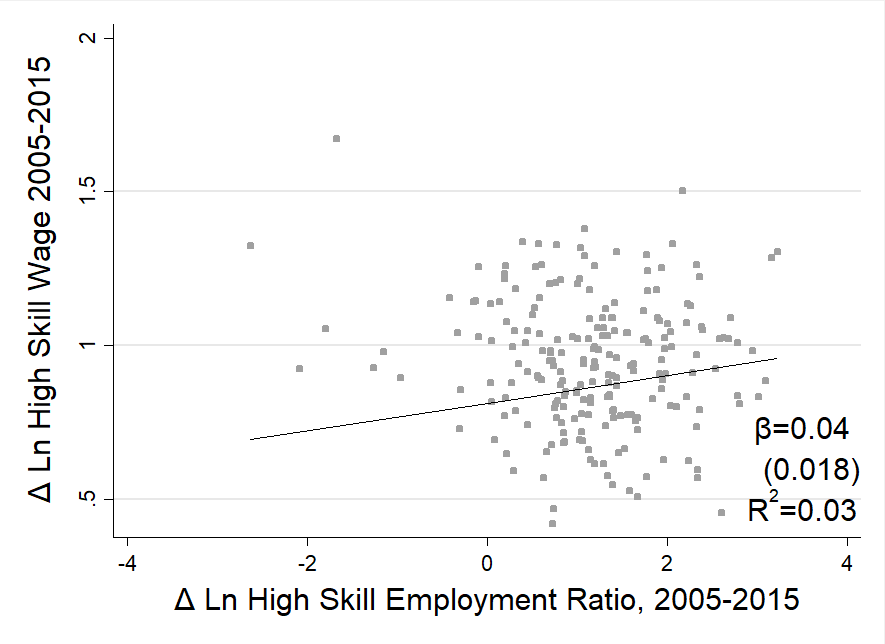

Supplement: S1 File — (ZIP) [file pone.0281669.s002.zip › S2_replication/S2_replication_data_and_program/Fig6c.tif]

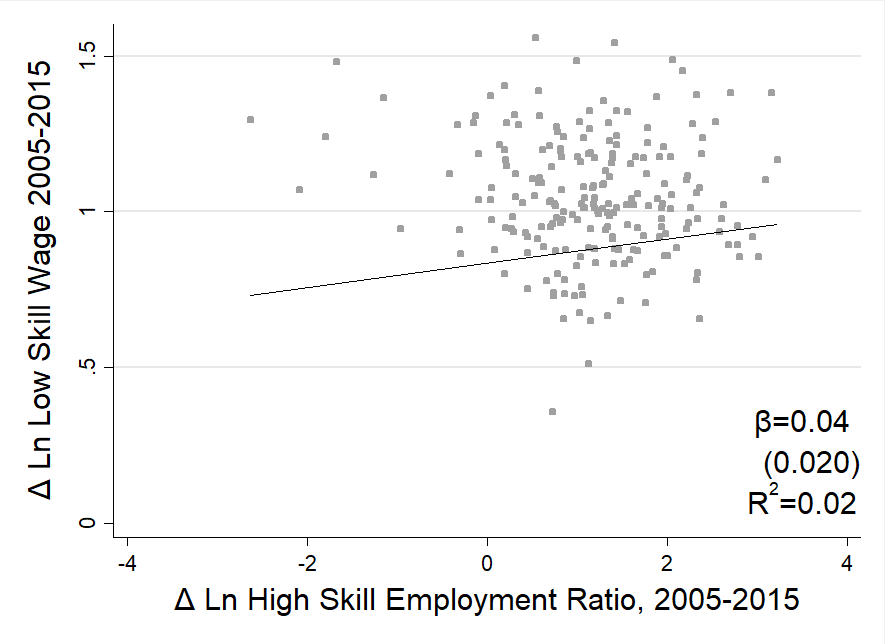

Supplement: S1 File — (ZIP) [file pone.0281669.s002.zip › S2_replication/S2_replication_data_and_program/Fig6d.tif]
